# Therapeutic Potential of Pharmacological Targeting NLRP3 Inflammasome Complex in Cancer

**DOI:** 10.3389/fimmu.2020.607881

**Published:** 2021-02-03

**Authors:** Gulcin Tezcan, Ekaterina E. Garanina, Mohammad Alsaadi, Zarema E. Gilazieva, Ekaterina V. Martinova, Maria I. Markelova, Svetlana S. Arkhipova, Shaimaa Hamza, Alan McIntyre, Albert A. Rizvanov, Svetlana F. Khaiboullina

**Affiliations:** ^1^ Institute of Fundamental Medicine and Biology, Kazan Federal University, Kazan, Russia; ^2^ Department of Fundamental Sciences, Faculty of Dentistry, Bursa Uludag University, Bursa, Turkey; ^3^ Centre for Cancer Sciences, Faculty of Medicine and Health Sciences, University of Nottingham, Nottingham, United Kingdom; ^4^ Department of Microbiology and Immunology, University of Nevada, Reno, NV, United States

**Keywords:** NLRP3, inflammasome, cancer, IL-1β, nigericin

## Abstract

**Introduction:**

Dysregulation of NLRP3 inflammasome complex formation can promote chronic inflammation by increased release of IL-1β. However, the effect of NLRP3 complex formation on tumor progression remains controversial. Therefore, we sought to determine the effect of NLRP3 modulation on the growth of the different types of cancer cells, derived from lung, breast, and prostate cancers as well as neuroblastoma and glioblastoma *in-vitro*.

**Method:**

The effect of Caspase 1 inhibitor (VX765) and combination of LPS/Nigericin on NLRP3 inflammasome activity was analyzed in A549 (lung cancer), MCF-7 (breast cancer), PC3 (prostate cancer), SH-SY5Y (neuroblastoma), and U138MG (glioblastoma) cells. Human fibroblasts were used as control cells. The effect of VX765 and LPS/Nigericin on NLRP3 expression was analyzed using western blot, while IL-1β and IL-18 secretion was detected by ELISA. Tumor cell viability and progression were determined using Annexin V, cell proliferation assay, LDH assay, sphere formation assay, transmission electron microscopy, and a multiplex cytokine assay. Also, angiogenesis was investigated by a tube formation assay. VEGF and MMPs secretion were detected by ELISA and a multiplex assay, respectively. Statistical analysis was done using one-way ANOVA with Tukey’s analyses and Kruskal–Wallis one-way analysis of variance.

**Results:**

LPS/Nigericin increased NRLP3 protein expression as well as IL-1β and IL-18 secretion in PC3 and U138MG cells compared to A549, MCF7, SH-SY5Y cells, and fibroblasts. In contrast, MIF expression was commonly found upregulated in A549, PC3, SH-SY5Y, and U138MG cells and fibroblasts after Nigericin treatment. Nigericin and a combination of LPS/Nigericin decreased the cell viability and proliferation. Also, LPS/Nigericin significantly increased tumorsphere size in PC3 and U138MG cells. In contrast, the sphere size was reduced in MCF7 and SH-SY5Y cells treated with LPS/Nigericin, while no effect was detected in A549 cells. VX765 increased secretion of CCL24 in A549, MCF7, PC3, and fibroblasts as well as CCL11 and CCL26 in SH-SY5Y cells. Also, VX765 significantly increased the production of VEGF and MMPs and stimulated angiogenesis in all tumor cell lines.

**Discussion:**

Our data suggest that NLRP3 activation using Nigericin could be a novel therapeutic approach to control the growth of tumors producing a low level of IL-1β and IL-18.

## Introductıon

A chronic inflammatory microenvironment is one of the predisposing factors that can stimulate malignant transformation (1). The tumor microenvironment contains diverse inflammatory constituents, leukocytes, pro-inflammation cytokines and tumor cells (2), which can promote angiogenesis, tumor growth and metastasis (2). The inflammasome, an intracellular oligomeric protein complex, plays a key role in the regulation of inflammation (3). Inflammasomes are activated in response to pathogen-associated molecular patterns (PAMPs) and damage-associated molecular patterns (DAMPs) released from infected cells, damaged tissues and tumors (4). PAMPs and DAMPs bind to Toll-like receptors (TLR) and trigger the expression of pro-inflammatory IL-1β and IL-18 cytokines (5). The most characterized inflammasome, Nucleotide-binding domain Leucine-rich Repeat (NLR) and Pyrin domain containing receptor 3 (NLRP3), is tightly regulated in resting cells (6). However, altered expression of NLRP3 was found in several pathological conditions, including cancer (6).

NLRP3 requires two signals: priming and activation (7). Binding of PAMPs and DAMPs to TLRs prime the cells and activate NLRP3 and Pro-Caspase 1 transcription (6, 8). Danger associated ligands such as pore formation and potassium (K+) efflux (9, 10), lysosomal destabilization/rupture (11, 12) and mitochondrial reactive oxygen species release (ROS) (13) provide the second signal promoting the assembly of an adaptor, apoptosis-associated speck-like protein containing a CARD (ASC) and Pro-Caspase 1 to form a functional inflammasome complex (14, 15). The inflammasome then cleaves the Caspase 1 which, subsequently releases active IL-1β and IL-18 (16).

The role of inflammasomes was extensively investigated in macrophages and dendritic cells (17). However, recently it was demonstrated that NLRP3 activation is not an exclusive feature of immune cells, and it was also detected in tumor cells (18–21), while its role in tumorigenesis remains controversial. Some reports indicate that aberrant activation of inflammasomes promotes carcinogenesis and maintains the malignant microenvironment in breast cancer, fibrosarcoma, gastric carcinoma, and lung metastasis (22–24). In contrast, the anti-cancer effect of inflammasomes *via* induction of pyroptosis and activation of the immune response was shown to protect against colorectal cancer (25).

Recently, multiple inhibitors and activators of the NLRP3 inflammasome pathway were investigated as potential therapeutics for inflammasome-linked diseases (26, 27). However, the efficacy of inflammasome targeting treatment in cancers remains unclear, due to the conflicting results obtained using tumor cells. The anti-tumor activity of several compounds targeting NLRP3 inflammasomes was studied, including Nigericin and VX-765. Nigericin, a microbial toxin, activates NLRP3 by inducing K^+^ efflux leading to the caspase-1 release and IL-1β secretion (28, 29). The anti-tumor effect of Nigericin was demonstrated in several carcinomas (30–34), however, it remains unknown whether the anti-tumor effect of Nigericin is linked to NLPR3. VX765 can block the caspase-1 ability to proteolytically cleave pro-IL-1β and pro-IL-1 (26, 35). Although recent studies demonstrated the potential therapeutic efficacy of VX765, its effect on tumor cells is largely unknown (26, 35, 36).

Therefore, in this study, we sought to investigate *in vitro* effects of Nigericin and VX765 on multiple tumor cell types derived from a variety of cancers, including lung, breast, prostate, as well as neuroblastoma and glioblastoma. We found that tumors demonstrate different levels of NLRP3 inflammasome activation and IL-1β and IL-18 secretion. In tumor cell lines where NLRP3 activation and, IL-1β and IL-18 secretion are low, Nigericin demonstrated an anti-tumor effect. In contrast, in tumor cell lines where NLRP3 activation and, IL-1β and IL-18 secretion are high, although Nigericin triggers initial tumor cell death, cells recover and tumors remain active.

## Methods

### Cell Lines

Cell lines, A549 (non-small-cell lung cancer cell line), MCF7 (breast adenocarcinoma cell line), PC3 (prostatic small-cell-carcinoma cell line), and SH-SY5Y (neuroblastoma cell line) were purchased from the American Type Culture Collection (ATCC; Rockville, USA). U138MG, a glioblastoma cell line was kindly provided by Prof. Dr. Berrin Tunca (Uludag University of Turkey). Cells were maintained in Dulbecco’s modified Eagle medium (DMEM) supplemented with 10% fetal bovine serum (FBS, Atlanta Biologicals), 2 mM L-glutamine, 25 U/ml penicillin, and 25 μg/ml streptomycin. All cell lines were grown at 37°C in a humidified chamber supplemented with 5% CO_2_.

### Primary Cell Isolation and Maintenance

Primary fibroblast cells were isolated from human healthy skin biopsies which were collected after the cosmetic surgery. Skin fragments were crushed into small pieces (1–2 mm) and incubated in DMEM Low Glucose Medium (PanEco, Moscow, Russia), supplemented with 10% FBS (HYCLONE, Utah, USA), 50 U/ml of penicillin, 50 µg/ml of streptomycin (PanEco, Moscow, Russia), and 2 mM L-glutamine for seven days in a 5% CO_2_ humidified incubator at 37°C. On day 7, the culture medium was replaced, and cells were cultured for 16–21 days. Cells in their 4^th^ passage were used in the experiments.

HUVECs were isolated from umbilical cord vein as described previously [45–46]. Briefly, the umbilical vein was washed with Dulbecco’s phosphate-buffered saline (DPBS) (PanEco, Moscow, Russia). The umbilical vein was subjected to enzymatic dissociation using 0.25% trypsin–EDTA (PanEco, Moscow, Russia) for 20 min and detached endothelial cells were collected. Collected cells were stained with anti-CD31 (PECAM-1) (SC-13537), anti-CD105 (Endoglin) (sc-18838), and anti-CD146 (MelCam) (sc-18837 PE) (all from Santa Cruz Biotechnology, CA) and analyzed using flow cytometry on FACS Aria III (Becton, Dickinson and Company, Becton Drive Franklin Lakes, NJ) to investigate their identity. Primary HUVEC cells were cultured in the standard medium supplemented with, 1% of nonessential amino acids (Gibco, Life Technologies, MA, USA), 5 U/ml of heparin and 10 mg/L Endothelial cell growth supplement (Sigma, St. Louis, USA), 10 ng/ml FGF2, 10 ng/ml vascular endothelial growth factor (GenScript, NJ, USA), 10 ng/ml epithelial growth factor (GenScript, NJ, USA), 10 ng/ml insulin-like growth factor (GenScript, NJ, USA). Cells were maintained in a 5% CO_2_ humidified incubator at 37°C and used up to the 4^th^ passage.

### Ethics Statement

This study was done in accordance with the recommendations of the Biomedicine Ethics Expert Committee of Kazan (Volga region) Federal University, the Republic of Tatarstan, Russian Federation with written informed consent from all subjects. All subjects gave written informed consent in accordance with the Declaration of Helsinki. Human tissue sample collection was approved by the local Ethical Committee of Kazan (Volga region) Federal University based on article 20 of the Federal Legislation on “Health Protection of Citizens of the Russian Federation” № 323-FL, 21.11.2011. Signed informed consent was obtained from each donor.

### Western Blot

Total protein extracts were prepared using Sodium dodecyl sulfate (SDS) reducing buffer (Biorad, CA, USA), separated on 8–12% gradient SDS polyacrylamide gels and transferred on Polyvinylidene difluoride (PVDF) membranes (Biorad, CA, USA). Membranes were blocked [Tris-buffered saline (TBS), 0.1% Tween 20, 5% BSA] for 1 h followed by overnight incubation with the monoclonal rabbit anti-human NLRP3 (1:300, Invitrogen, IL, USA) antibody at 4°C. Membranes were washed with TBS and 0.1% Tween 20 and incubated for 1 h at room temperature with anti-rabbit IgG (1:1,000, Santa Cruz Biotechnology, Germany) and mouse anti-human Actin Beta-HRP conjugated (1:1,000, Sigma) antibodies. Western blot results were visualized using Clarity Western ECL reagents (Biorad, CA, USA) and a ChemiDoc XRS + (Biorad, CA, USA). Protein levels were quantified using NIH ImageJ software version 1.52a.

### Enzyme-Linked Immunosorbent Assay

Levels of IL-1β, IL-18, and VEGF were measured using commercially available ELISAs (VECTOR-BEST, Novosibirsk, Russia). Each ELISA analysis was done in triplicate. To quantify IL-1β, IL-18, and VEGF levels, the absorbance of samples at 490 and 680 nm were measured using TECAN Infinite 200 Pro fluorimeter (Grödig, Austria). The 680 nm absorbance (background signal from the instrument) value was subtracted from the 490 nm absorbance value.

### Annexin V Analysis

Cell viability was assessed using APC Annexin V Apoptosis Detection Kit with Propidium Iodide (Sony Biotechnology, USA) according to manufacturer’s protocol. Stained cells were immediately analyzed by flow cytometry using BD FACSAria III (BD Biosciences, USA) and data processed with FlowJo software package (FlowJo LLC, USA).

### Real-Time Cell Proliferation Assay

The xCELLigence biosensor cell analysis system (ACEA Biosciences, USA) was used for the real-time monitoring of cell proliferation. Cells (5 × 10^3^) were seeded in each well of E-plate 16 (ACEA Biosciences, USA) for 24 h and used to determine the cell index every 15 min.

### Lactate Dehydrogenase Assay

Cells (2 × 10^4^ cells/well) were plated in 96‐well plates and incubated overnight (37°C, CO_2_). The medium was removed, and cells were incubated with fresh medium containing VX765, lipopolysaccharide (LPS) and Nigericin for 24 h. Medium (50 μl) was collected and used for the LDH assays. Untreated cells were treated with the lysis solution for 45 min and used as a positive control to determine the maximum LDH release. The culture medium was used as a negative control. LDH released by cells into the medium was determined using the Pierce™ LDH cytotoxicity assay kit (Thermo Scientific, Pierce Biotechnology, Rockford, IL, USA) according to the manufacturer’s instructions. To quantify LDH activity, the absorbance of samples at 490 and 680 nm was measured using TECAN Infinite 200 Pro fluorometer (Grödig, Austria). The 680 nm absorbance (background signal from the instrument) value was subtracted from the 490 nm absorbance value. The cytotoxicity (%) was calculated by (test sample − negative control)/(positive control − negative control) × 100.

### Sphere Formation Assay

Plates (24 well) were pre-coated with 250 μl of Matrigel (BD Biosciences, Franklin Lakes, NJ, USA) and incubated at 37°C for 30 min. Tumor cells (1 × 10^4^) were seeded onto the Matrigel-coated plate and maintained in the culture medium (5% CO_2_, 37°C). After 5 days, tumorspheres were analyzed using a Zeiss Observer Z1 inverted microscope (Göttingen, Germany). The size of spheres was measured using Axiovision Rel 4.5 software (Göttingen, Germany).

### JC-1 Staining to Detect Mitochondrial Membrane Potential (Δψm)

Cell monolayers were washed with PBS and incubated with 8 µM of JC-1 solution at 37°C for 20 min. The supernatant was removed; cell monolayers were washed twice with PBS and incubated in medium for 24 h in 5% CO_2_ before analyzing by flow cytometry using a BD FACSAria III (BD Biosciences, USA). Data were processed using FlowJo software package (FlowJo LLC, USA). The excitation wavelength was 490 nm at which the JC-1 monomer was detected, and the emitting wavelength was set to 530 nm, at which the JC-1 polymer was detected.

### Cytokine Assay

The Bio-Plex Pro™ Human Chemokine Panel, 40-Plex and Bio-Plex Pro™ Human MMP Panel, 9-Plex were used to analyze samples according to the manufacturer’s recommendations. Fifty microliters of sample were used for determining cytokine concentration and data was analyzed using a Luminex 200 analyzer with MasterPlex CT control software and MasterPlex QT analysis software (MiraiBio division of Hitachi Software San Francisco, CA, USA).

### Transmission Electron Microscopy

Transmission electron microscopy (TEM) was done using 0.3x10^6^ cells following a standard protocol. Briefly, cells were fixed in a 2.5% phosphate-buffered glutaraldehyde solution (Merck, Kenilworth, NJ, USA) at 4˚C for 24 h. Cells were then washed (2x PBS) and fixed with 1% OsO_4_ (Electron Microscopy Sciences, Hatfield, USA) at room temperature for 1 h, dehydrated using an ethanol gradient, and embedded in EPON (Electron Microscopy Sciences, Hatfield, USA). Ultrathin sections (0,1 µm) were contrasted with uranyl acetate and lead citrate and examined using a transmission electron microscope (Hitachi HT7700, Tokyo, Japan).

### Endothelial Cell Tube Formation Assay

96-well plates were pre-coated with 50 μl of growth factor-reduced Matrigel (BD Biosciences, Franklin Lakes, NJ, USA) and incubated at 37°C for 30 min. HUVECs (2 × 10^4^) were seeded in the Matrigel-coated wells and incubated with cell-free supernatants derived from VX765, LPS and Nigericin treated cells. VEGF (10 ng/ml) was used as a positive control. Tube morphology was analyzed at 24 h and two wells per group were counted. Tube numbers and branch length were measured and analyzed using NIH ImageJ software version 1.52a.

### Statistical Analyses

Statistical analysis was done using the IDE RStudio for the R (version 3.6.0) software (RStudio, Boston, MA, USA). One-way ANOVA with Tukey’s *post hoc* analysis was utilized to evaluate the findings of ELISA, Annexin V, JC1, and sphere formation assays, where the data were parametric. The Kruskal–Wallis one-way analysis of variance for comparisons between individual experimental groups was utilized for statistical analysis of the LDH, Multiplex analyses and Tube formation assays, where the data were nonparametric. Data are presented as mean ± SE. Significance was established at a value of p <0.05.

## Results

### Analysis of NLRP3 Activation and Inhibition After Nigericin and VX765 Treatment

**Figure 1:**
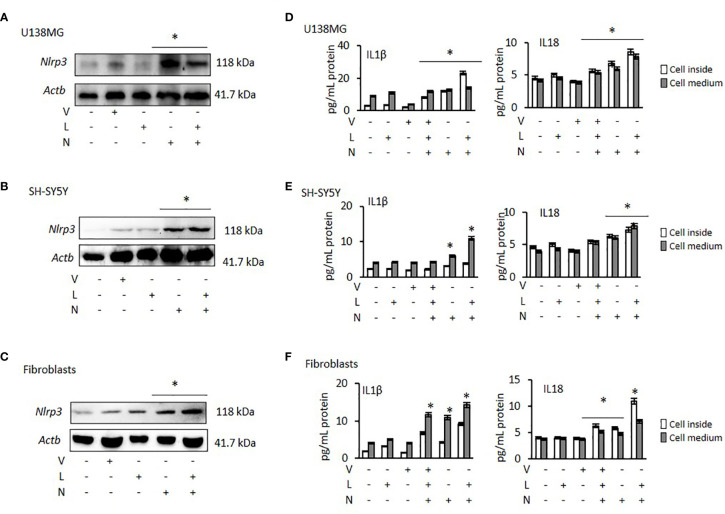
Effect of NLRP3 modulation in U138MG and SH-SY5Y cells, where one of the highest and one of the lowest NLRP3 expression was observed and in Fibroblasts: Nigericin (20 µM, Invivogen) treatment for 24 h with and without 3 h pre-incubation with LPS (1 µg/ml, Sigma, St. Louis, USA) was used to activate the NLRP3 inflammasome. To inhibit Caspase 1, cells were treated with VX765 (20 µM, Invivogen). **(A–C)** NLRP3 protein expression was demonstrated by western blot. **(D–F)** IL-1β and IL-18 levels were quantified, from inside the cells and in the medium representing levels secreted from the cells, by ELISA. the cells by ELISA. U, Untreated; V, VX765; L, LPS; N, Nigericin; LN, LPS/Nigericin. *p < 0.05, n = 3.

NLRP3 activation requires two signals: priming with lipopolysaccharide (LPS) followed by activation, using Nigericin or ATP (37). Therefore, to activate NLRP3 we treated all cancer cells and fibroblasts with LPS, followed by Nigericin. Nigericin and combination of LPS with Nigericin (LPS/Nigericin) increased NLRP3 protein expression in all tumor cell lines as compared to untreated tumor cells and in Nigericin and LPS/Nigericin treated fibroblasts as compared to untreated fibroblasts (**Figures 1A–C** and **Supplementary Figures 1A–C**). Additionally, production IL-1β and IL-18 and their levels in culture medium were increased in cells treated with the combination of LPS/Nigericin (**Figures 1D–F**, **Supplementary Figures 1D–F**). These data confirm that LPS/Nigericin induces the formation of NLRP3 inflammasome complex in all tumor cell lines and the fibroblasts. After LPS/Nigericin treatment, the IL-1β production was higher in U138MG (23.10 pg/ml cytosol vs 14.02 pg/ml medium) (**Figure 1D**) and PC3 (42.34 pg/ml cytosol vs 9.95 pg/ml medium) (**Supplementary Figure 1D**) cells compared to untreated controls. In addition, after LPS/Nigericin treatment, IL-1β production was higher in U138MG and PC3 cells compared to LPS/Nigericin treated Fibroblasts (p <0.001 and p <0.001). In fibroblasts, the IL-1β production was 9.16 pg/ml cytosol vs 14.26 pg/ml medium after LPS/Nigericin treatment (**Figure 1F**, **Table 1**). In contrast, IL-1β production and secretion were the lowest in SH-SY5Y cells incubated with LPS/Nigericin (IL-1β: 3.77 pg/ml cytosol vs 10.93 pg/ml medium) (**Figure 1E**). IL-1β production in SH-SY5Y cells was significantly lower than in fibroblasts (IL-1β: 9.16 pg/ml cytosol vs 14.26 pg/ml medium) (p <0.001 and p <0.001). Additionally, although, IL-1β secretion was induced in LPS/Nigericin treated MCF7 (11.89 pg/ml cytosol vs 7.85 pg/ml medium) (**Supplementary Figure 1E**) and A549 cells (9.36 pg/ml cytosol vs 7.67 pg/ml medium) (**Supplementary Figure 1F**), when compared to untreated MCF7 and A549 cells, the production of IL-1β was lower than in LPS/Nigericin treated U138MG and PC3 cells.

**Table 1:** Summary of the significantly altered cytokines in tumor cells and fibroblasts following NLRP3 modulation (compared to untreated controls) (p < 0.05, full data available in **Supplementary Table 4**).

	VX765	LPS	Nigericin	LPS& Nigericin
A549	↓CXCL1			↓CXCL1, IL6, CXCL8, CCL13, CCL22
		↑GMCSF	↑MIF	↑GMCSF, MIF
MCF	–	↑CCL23	↑CXCL8, CCL13	↑CCL21, CCL27, CXCL5, CCL11, CCL26, CXCL6, GMCSF, CXCL1, CXCL2, CCL1, IFNg, IL4, CXCL8, IL10, CCL8, CCL7, CCL13, CXCL9, CCL20, CCL23, CCL25
PC3	–	–	↑MIF	↑CCL21, CCL11, GMCSF, CCL1, IL4, CCL8, CCL22, CXCL9, CCL3, CCL23, CCL17, TNFa
SH-SY5Y	↑CCL11, CCL26	↑CCL21, CCL11, CCL24, CCL22, TNFa	↑MIF	↑CXCL5, CCL24, IL2, CCL7, CCL22, CCL3, TNFa
U138MG	–	–	↑MIF	↑MIF
Fibroblasts	–	↑CCL21, CXCL13, CCL27, CXCL5, CCL11, CCL24, CCL26, GMCSF, CXCL1, CXCL2, CCL1, IL2, IL4, IL6, CXCL8, IL10, IL16, CXCL10, CXCL11, CCL2, CCL8, CCL7, CCL22, CXCL9, CCL5, CCL20, CCL19, CCL23, CXCL16, CXCL12, CCL17, CCL25	↑MIF	–

Similar to IL-1β, IL-18 production and secretion were increased in all tumor cell lines treated with LPS/Nigericin compared to untreated control (**Table 1**). IL-18 production was higher in PC3 cells (13.83 pg/ml cytosol vs 11.40 pg/ml medium) (**Supplementary Figure 1D**) compared to LPS/Nigericin treated fibroblasts (10.96 pg/ml cytosol vs 7.10 pg/ml medium) (p <0.001 and p <0.001). In contrast, LPS/Nigericin leaded lower IL-18 production and release in SH-SY5Y (7.26 pg/ml cytosol vs 7.81 pg/ml medium) (**Figure 1E**), MCF7 (7.29 pg/ml cytosol vs 6.88 pg/ml medium) (**Supplementary Figure 1E**) and A549 cells (10.39 pg/ml cytosol vs 5.52 pg/ml medium) (**Supplementary Figure 1F**) compared to similarly treated fibroblasts (p < 0.001). Interestingly, there were no statistically significant differences in IL-18 production in U138MG (8.57 pg/ml cytosol vs 7.80 pg/ml medium) (**Figure 1D**), SH-SY5Y cells (7.26 pg/ml cytosol vs 7.81 pg/ml medium), and fibroblasts (10.96 pg/ml cytosol vs 7.10 pg/ml medium) after LPS/Nigericin treatment (**Figure 1F**, **Table 1**). Collectively, our findings demonstrate that IL-1β production is a more robust indicator than IL-18, of NLRP3 activation across the panel of cells investigated here. The NLRP3 activation ability and IL-1β, IL-18 secretions were also confirmed after treatment with LPS/ATP in PC3 (one of the high NLRP3 expressed cell lines) and MCF7 (one of the low NLRP3 expressed cell lines) cells. After LPS/ATP treatment, the expression levels of NLRP3 were similar to that of cells after LPS/Nigericin treatment (**Supplementary Figure 2**).

VX765 as a single agent did not significantly affect NLRP3 protein expression (**Figures 1A–C**, **Supplementary Figures 1A–C**) in any cell lines. When cells were treated with VX765, the effect of LPS/Nigericin on IL-1β and IL-18 release was significantly reduced in most of the cell lines investigated (p < 0.05; **Figures 1D–F**, **Supplementary Figures 1D–F**, **Supplementary Table 1**). As an inflammasome inhibitor, the effect of VX765 was confirmed by another NLRP3 inhibitor, Glybenclamide in PC3 and MCF7 cells (**Supplementary Figure 2**). These data suggest that VX765 did not affect on NLRP3 expression but has an indirect mechanism of action of inflammasome inhibition, potentially *via* hindering Caspase 1 activity whereas Glybenclamide directly suppressed NLRP3 expression.

### Nigericin Inhibits, While VX765 Stimulates Tumor Cell Viability and Proliferation

**Figure 2:**
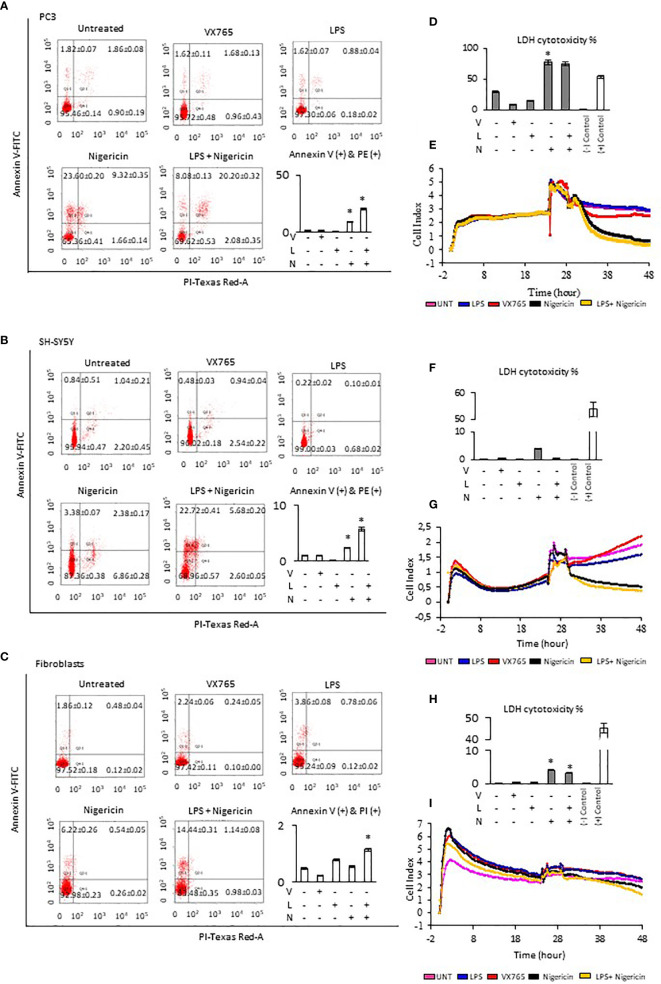
Effect of NLRP3 inhibition and stimulation on cell viability and proliferation of PC3, SH-SY5Y cells, and Fibroblasts. Nigericin (20 µM), treatment for 24 h with and without 3 h pre-incubation with LPS (1 µg/ml), was used to activate the NLRP3 inflammasome. To inhibit Caspase 1, cells were treated with VX765 (20 µM). **(A–C)** Expression of Annexin V **(D, F, H)** LDH cytotoxicity assay and **(E, G, I)** a real-time cell proliferation assay. U, Untreated; V, VX765; L, LPS; N, Nigericin; LN, LPS/Nigericin. *p < 0.05, n = 3.

The effect of Caspase 1 inhibition and NLRP3 activation on cell viability was demonstrated using Annexin-V-FITC/PI assay. Cells demonstrate both, Annexin V and PI when non-apoptotic programmed cell deaths is induced (38). Tumor cell lines and fibroblasts were treated with VX765, LPS, Nigericin and LPS/Nigericin. VX765 had a limited effect on the percentage of Annexin V and PI positive cells in all cell lines (p > 0.05, **Figure 2**, **Supplementary Figure 3**, **Supplementary Table 2**). LPS decreased the percentage of PC3 and SH-SY5Y cells positive for Annexin V and PI compared to untreated controls (PC3 and SH-SY5Y: p < 0.05, **Figures 2A, B**), while the number of Annexin V and PI positive A549, MCF7 and U138MG cells were not affected (A549, MCF7, and U138MG: p > 0.05, **Supplementary Figures 2A, B**). LPS increased the percentage of fibroblasts positive for Annexin V & PI compared to untreated controls (p = 0.013, **Figure 2C**).

Treatment with Nigericin alone significantly increased the percentage of Annexin V and PI positive cells in all cell lines compared to untreated controls except MCF7 and fibroblasts (p < 0.05). (**Figure 2**, **Supplementary Figure 2**). Similarly, LPS/Nigericin significantly increased the percentage of Annexin V and PI positive cells in all cell lines (p < 0.001, **Figure 2**, **Supplementary Figure 3**, **Supplementary Table 2**). The effect of Nigericin and LPS/Nigericin on PC3 cells, where NLRP3 expression was the highest, and SH-SY5Y, where NLRP3 activation was the lowest is shown in **Figures 2A, B**. The percentage of Annexin V & PI positive cells after Nigericin and LPS/Nigericin treatments were significantly higher in PC3 cells compared to fibroblasts (p < 0.001 and p < 0.001). In addition, although the percentage of Annexin V & PI cells in Nigericin and LPS/Nigericin treated SH-SY5Y cells was higher than in fibroblasts (p < 0.001 and p < 0.001), it was significantly lower than in equivalently treated PC3 cells (p < 0.001 and p < 0.001). In U138MG cells, where Nigericin and LPS/Nigericin induced one of the highest NLRP3 expressions, the percentage of Annexin V & PI cells was significantly higher than in equivalently treated fibroblasts (p < 0.001 and p < 0.001). Similarly, in MCF7 and in A549 cells, where Nigericin and LPS/Nigericin induced low inflammasome expression, the percentage of Annexin V & PI cells was significantly higher compared to equivalently treated fibroblasts. It should be noted that the percentage of Annexin V & PI cells in MCF7 and A549 cells was lower than in equivalently treated PC3 and U138MG cells. The effect of Nigericin and LPS/Nigericin on U138MG, MCF7, and A549 cell vitality is shown in **Supplementary Figures 3 A–C**. The effect of NLRP3 stimulation and inhibition on tumor cell viability was also confirmed by activating NLRP3 by LPS/ATP and suppressing NLRP3 using glibenclamide. Similar to LPS/Nigericin, after LPS/ATP treatment the cell death increased as compared to untreated tumors whereas glibenclamide decreased the rate of LPS/ATP mediated cell death (**Supplementary Figure 4**).

Next, LDH levels were analyzed to determine plasma membrane integrity in cells after modulation of NLRP3 activity. Nigericin triggered the highest level of LDH release in all cell lines compared to untreated controls (p < 0.001). Nigericin caused the highest release of LDH in PC3 (47.87% increase, **Figure 2D**) and U138MG (36.64% increase, **Supplementary Figure 3D**) compared to investigated cell lines. Also, the release of LDH was lower in MCF7 (25.08% increase, **Supplementary Figure 3F**) and A549 cells (13.22% increase, **Supplementary Figure 3H**) compared to investigated cell lines. Although the LDH release in SH-SY5Y cells was 3.50% greater than in untreated control (**Figure 2H**), it was still the lowest compared to all cell lines (**Figures 2D, F, H**; **Supplementary Figures 3D, F, H**, **Supplementary Table 3**). Additionally, LPS/Nigericin significantly increased LDH release in all cell lines investigated (p < 0.001). However, the impact of LPS/Nigericin on LDH release was lower than Nigericin treatment alone in all cell lines.

To determine whether NLRP3 could affect the tumor proliferation, cells were treated with VX765, LPS, Nigericin and LPS/Nigericin before real-time monitoring of cell division using the xCELLigence biosensor for 24 h. VX765 and LPS did not affect cell proliferation compared to untreated controls in all investigated cells. In contrast, Nigericin and LPS/Nigericin substantially inhibited cell proliferation in all tumor cell lines. The effect of NLRP3 modulation of cell proliferation in PC3 cells, where inflammasome expression was the highest, and in SH-SY5Y, with the lowest NLRP3 expressions upon after Nigericin and LPS/Nigericin treatment, are shown in **Figures 2E, G**. Additionally, the effect of NLRP3 modulation on cell proliferation in U138MG, MCF7 and A549 cells is shown in **Supplementary Figures 3E, G, I**. Interestingly, the proliferation pattern of fibroblasts did not change after treatment with Nigericin or LPS/Nigericin and it remained similar to that in untreated controls (**Figure 2I**). It appears that the effect of NLRP3 activation on tumor growth depends on the stimulus. While LPS did not significantly affect the cell viability and proliferation, adding the second stimulus, Nigericin, induced cell death.

**Figure 3:**
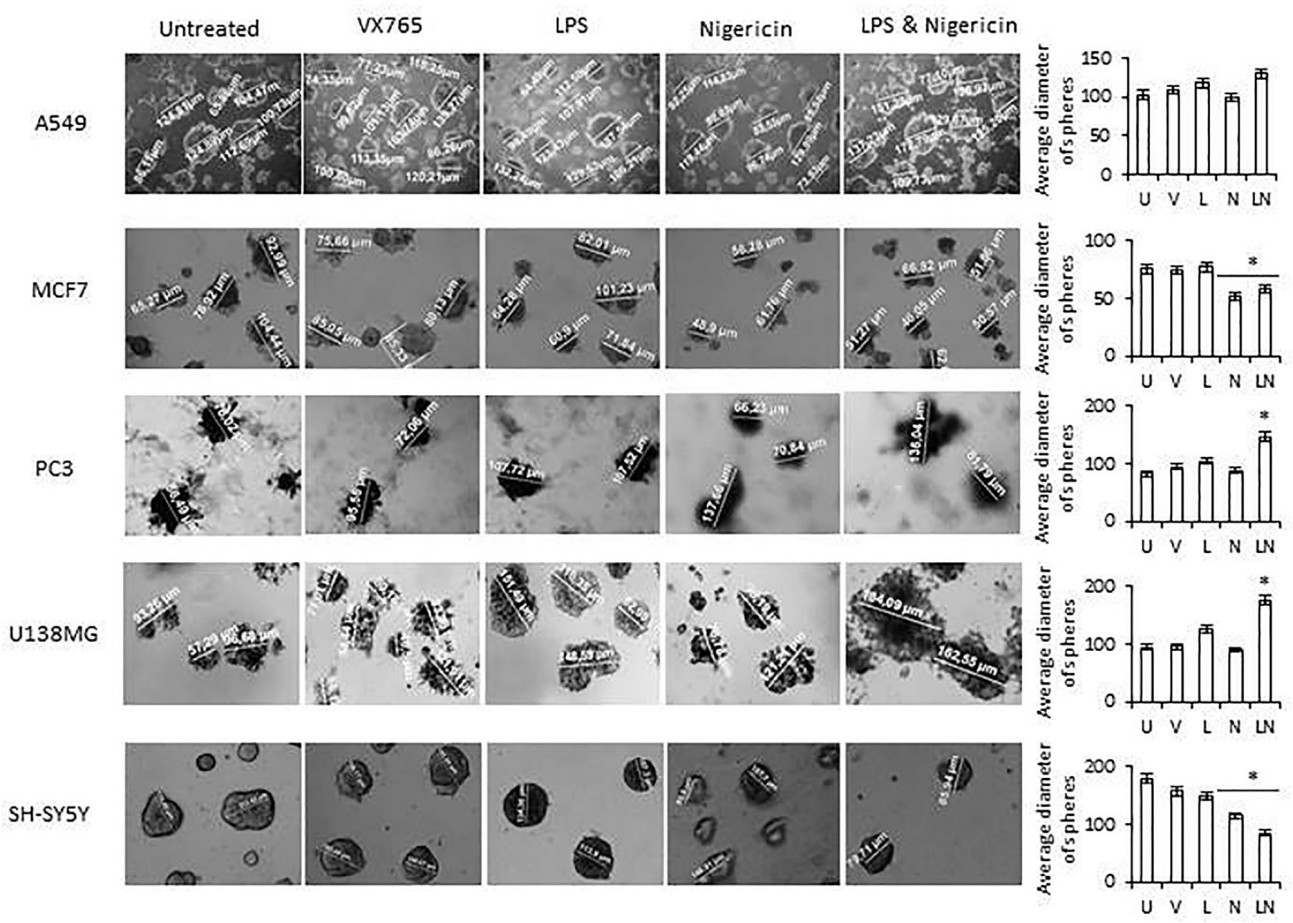
Effect of NLRP3 modulation on sphere formation of tumor cell lines. Nigericin (20 µM) treatment for 24 h with and without 3 h pre-incubation with LPS (1 µg/ml) was used to activate the NLRP3 inflammasome. To inhibit Caspase 1, cells were treated with VX765 (20 µM). U, Untreated; V, VX765; L, LPS; N, Nigericin; LN, LPS/Nigericin. *p < 0.05, n = 3.

The tumorsphere formation assay was used to assess stem cell-like characteristics of cancer cell lines after modulation of the NLRP3 inflammasome. Similar to our findings of cell viability and proliferation, VX765 and LPS did not affect the tumorsphere size (**Figure 3**). In contrast, while Nigericin and LPS/Nigericin consistently decreased cell proliferation and increased cell death in tumor cell monolayers, they had different effects on tumorsphere size across the cell lines investigated. In MCF7 and SH-SY5Y cells, Nigericin and LPS/Nigericin treatments significantly decreased sphere sizes compared to untreated controls (P < 0.001, **Figure 3**). However, in PC3 and U138MG cells, Nigericin did not affect tumor size, while LPS/Nigericin significantly increased the sphere sizes compared to untreated controls (P < 0.001, **Figure 3**). Nigericin and LPS/Nigericin did not affect the sphere size in A549 cells compared to untreated controls (**Figure 3**). Collectively, our data suggest that high activation of NLRP3 by LPS/Nigericin results in the growth of tumorsphere whereas low NLRP3 activation by LPS/Nigericin results in a reduction of tumorsphere size.

### VX765 Increases the Release of CCL24, While Nigericin Increases MIF Production

**Figure 4:**
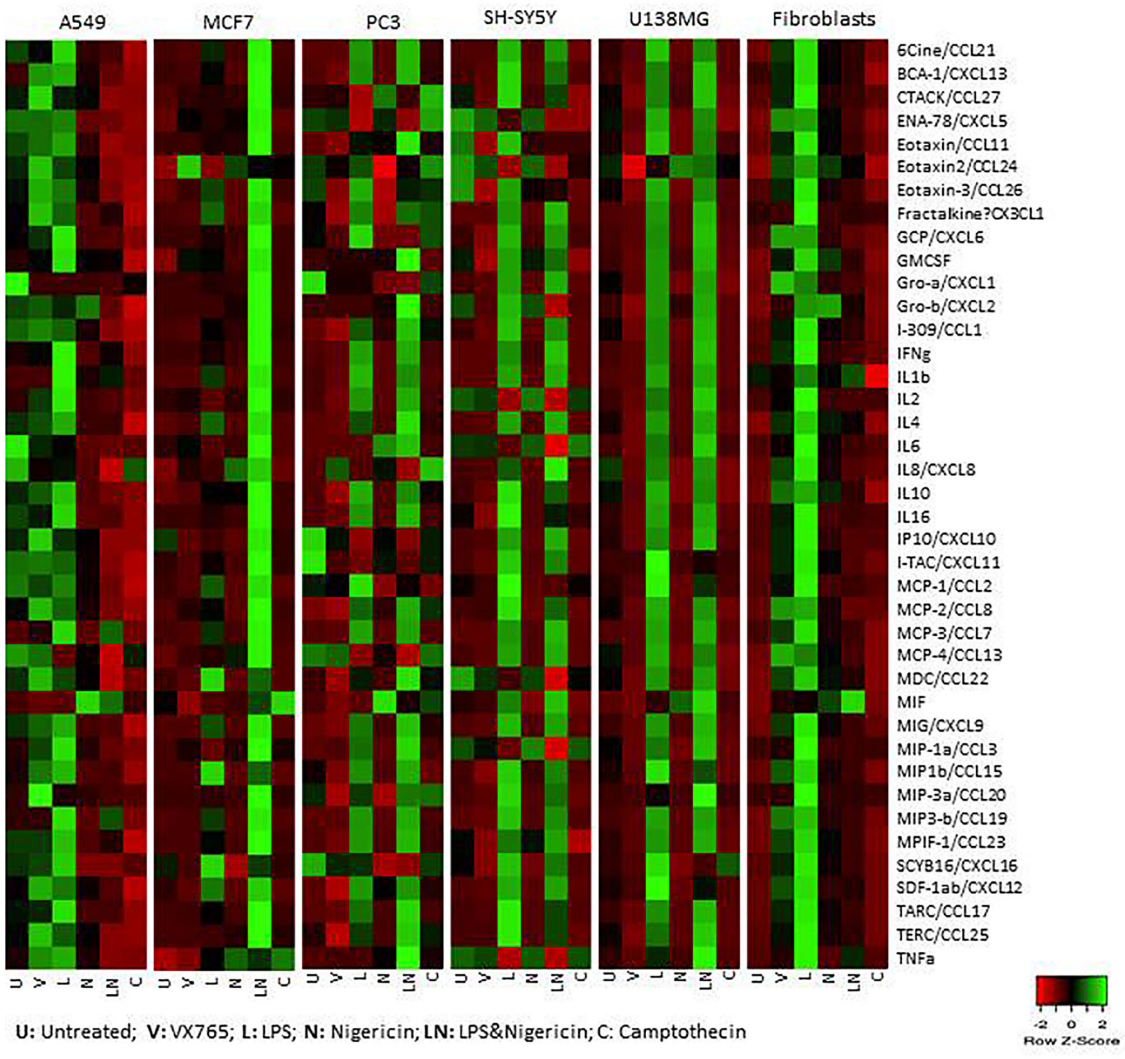
Cytokine release patterns in A549, MCF7, PC3, SH-SY5Y, U138MG cells, and fibroblasts after NLRP3 modulation. Nigericin (20 µM), treatment for 24 h with and without 3 h pre-incubation with LPS (1 µg/ml) was used to activate the NLRP3 inflammasome. To inhibit Caspase 1, cells were treated with VX765 (20 µM). Camptothecin (6 µM, Sigma) treatment of cells for 24 h was used to induce apoptosis. U, Untreated; V, VX765; L, LPS; N, Nigericin; LN, LPS/Nigericin. N = 3.

Cell culture medium was collected 24 h after treatment with VX765, LPS, Nigericin and LPS/Nigericin and used to analyze cytokine release patterns. Additionally, to determine whether these cytokine patterns differ from those induced during apoptosis, the culture medium was collected from cells treated with Camptothecin (6 µM for 24 h), an apoptosis inducing agent. VX765, LPS, Nigericin and LPS/Nigericin significantly modified cytokine production by tumor cells and fibroblasts (**Figure 4** and **Table 1**). CCL24 release was commonly identified in A549, MCF7 and fibroblasts treated with VX765 compared to untreated controls (**Supplementary Tables 4A, B, F**). Interestingly, the effect of VX765 treatment differed in neuronal tumors where elevated levels of CCL11 and CCL26 were found in SH-SY5Y compared to untreated controls (**Supplementary Table 4D**), and no changes in cytokine levels were detected in VX765 treated U138MG cells compared to controls (**Supplementary Table 4E**). When NLRP3 was activated, it appears that LPS and Nigericin upregulated different cytokines in tumor cells (**Figure 4**, **Table 1**). While LPS increased the levels of multiple cytokines, Nigericin uniquely increased MIF levels in A549, PC3, SH-SY5Y, U138MG, and fibroblasts compared to untreated controls (**Supplementary Tables 4A, C–F**). Interestingly, a statistically significant increase in MIF levels was only found in SH-SY5Y cells when treated with LPS/Nigericin compared Camptothecin treated controls (p = 0.008, **Supplementary Table 4D**). Although not statistically significant, MIF expression was higher in A549, U138MG and fibroblasts treated with LPS/Nigericin compared to Camptothecin treated controls (**Supplementary Tables 4A, E**, and **F**). The exception was MCF7 cells, where Nigericin stimulated CXCL8 and CCL13 compared to untreated and Camptothecin treated controls without affecting MIF levels (**Supplementary Table 4B**).

Although the cytokine pattern did not change in cells treated with Caspase 1 inhibitor compared to untreated controls, the concentration of cytokines secreted differed significantly between cells treated with VX765 and LPS/Nigericin. Treatment with VX765 significantly increased the level of cytokines mediating angiogenesis (**Table 2**) compared to LPS/Nigericin in A549 and SH-SY5Y cells. In PC3 cells, although VX765 did not affect the release of most cytokines, CCL24 secretion was significantly higher compared to the LPS/Nigericin treated group (p < 0.05). In contrast, the level of angiogenic cytokines (**Table 2**) was significantly lower in VX765 treated cells than LPS/Nigericin treated PC3 cells. Similarly, the level of these angiogenic cytokines (**Table 2**) in VX765 treated MCF7 and U138MG was lower than LPS/Nigericin treated cells. Interestingly, VX765 did not affect the release of cytokines in fibroblasts. Cytokines significantly altered by VX765 compare to LPS/Nigericin treated cells are summarized in **Table 2** (**Figure 4**, **Supplementary Tables 4A–F**).

**Table 2:** Cytokine secretion was significantly altered by VX765 treatment compared to LPS/Nigericin treatment (p < 0.05).

Cell line	Regulation of cytokine release in VX765 treated cells compared to LPS/Nigericin treated cells	
	Increased cytokine release	Decrease cytokine release
A549	**IL6,** CXCL10, CXCL11, **CCL1**, **CCL8, CCL11**, **CCL13**, **CCL22**,**CCL24**, **CCL26**, CCL27, **TNFa**	
MCF7	-	IL10, **IL16**, **IFNg**, **CXCL2**, **CXCL6,** CXCL9,**CCL21**, CXCL10, CXCL11, **CXCL13**,**CCL1**, **CCL7**, **CCL11**, **CCL20**, **CCL23**, **CCL25, MIF**
PC3	**CCL24**	**IL2**, **IL4**, **IL6**, IL10, **IL16**, **IFNg**, **CXCL9**, **CXCL12**, **CXCL13**, **CCL7**, **CCL8**, **CCL11**, **CCL15**, CCL19, **CCL21**, **CCL22**, **CCL23**, **CCL25**, **TNFa**
SH-SY5Y	**IL4**, **IFNg**, **CXCL1**, **CX3CL1**, **CXCL8**, CXCL11, **CXCL16, CCL5**, **CCL7**, **CCL13, GMCSF**	-
U138	-	**IL2**, **IL4**, **IL6**, **IL16**, **CXCL2**, CXCL3, **CXCL6**, CXCL9, CXCL10, **CCL1**, **CCL7**, **CCL17**, **CCL20**, **CCL25**, **CCL26**, CCL27, **GMGSF**, **IFNg**, **MIF**, **TNFa**

*Cytokines denoted in bold induce angiogenesis (39–51).

**Figure 5:**
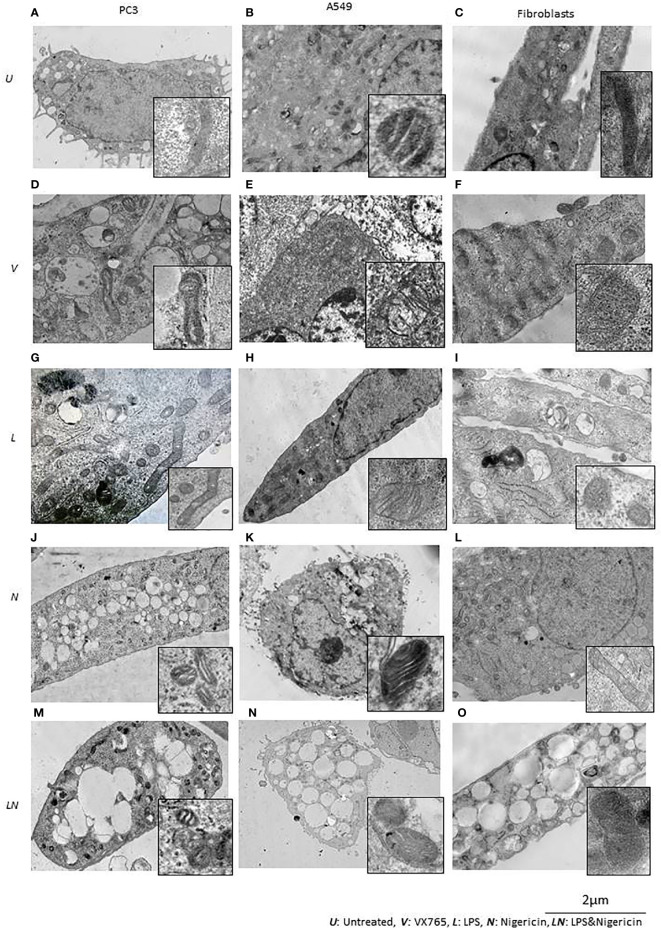
The effect of NLRP3 inflammasome modulation on cell morphology in A549, PC3, and human fibroblast cells. Nigericin (20 µM, Invivogen) treatment for 24 h with and without 3 h pre-incubation with LPS (1 µg/ml, Sigma, St. Louis, USA) was used to activate the NLRP3 inflammasome. To inhibit Caspase 1, cells were treated with VX765 (20 µM, Invivogen). **(A)** In untreated PC3 cells, small vacuoles were found in the cytoplasm, some of them contain electron-dense material. The cytoplasm is electron-dense containing many free ribosomes. **(B)** In untreated A549 cells, ER appears as narrow, elongated, rough tanks with a large number of free ribosomes. Also, oval-shaped mitochondria with clear cristae and an average electron density matrix are visualized. **(C)** In untreated fibroblasts, free ribosomes and polyribosomes were found in the cytoplasm. The shape of mitochondria was elongated with a condensed matrix and a large number of cristae. VX765 treatment: **(D)** significantly affected the ultrastructure of PC3 cells; increased numbers of large membrane-bound vacuoles were found in the cytoplasm, where some of them were merged or contained multi-vesicular aggregates. There was no visible difference in the ultrastructure of mitochondria between VX765 treated and untreated PC3 cell mitochondria. Cristae were slightly clearer compared to untreated cells. **(E)** In A549 cells, increased numbers of free ribosomes and granularization of ER were detected. Mitochondria were slightly enlarged and the outer membrane of mitochondria and their cristae became clear. **(F)** in fibroblasts, increased numbers of organelles, granularization of ER and changes to the ER structure to an expanded, branched, and irregular shape was identified. There were few free ribosomes and many polyribosomes. Additionally, the Golgi apparatus was expanded with a large number of vesicles. Mitochondria displayed a round or oval shape with condensed matrix and lacked distinct cristae. LPS treatment: **(G)** In PC3 cells, an increased number of organelles was identified. ER cisternae were expanded. The Golgi apparatus developed cistern stacks. Functional activity of mitochondria was increased: the ultrastructure of mitochondria was changed: the length of the mitochondria was considerably elongated, the matrix became electron-dense, and some of the cristae were visible. **(H)** In A549 cells, after LPS treatment; ER cisternae were expanded, had an irregular shape and formed a network. **(I)** In fibroblasts, similar to the effect of VX765; after LPS treatment protein synthesis and export were considerably increased; where ER cisternae were greatly expanded which had an irregular shape and formed a network. Additionally, the Golgi apparatus was visualized with well-developed cisternae stacks and a large number of vesicles in the cytoplasm. These vesicles were also detected near the cytoplasmic membrane of the cells; some of them were involved in exocytosis. After Nigericin or combined LPS and Nigericin treatments: **(J, M, K, N)**. PC3 and A549 cells showed signs of lytic cell death such as nuclear condensation, numerous round transparent vacuoles in the cytoplasm, pore formation in the cell membrane, cell swelling and bursting. The morphology of the mitochondria was significantly changed as compared to control where: the shape of the organelle was toroidal; the matrix became electron-dense with few elongated cristae or, the absence of cristae. **(L)**. Different from the tumor cell lines, in Fibroblasts, Nigericin treatment did not cause death cell morphology. Nigericin affected the nucleus structure where the integrity of the karyolemma appeared slightly destroyed. Mitochondria were round or oval, with clear cristae. The cytoplasm contained few free ribosomes and many polyribosomes. **(O)**. After combined LPS and Nigericin treatment fibroblasts demonstrated a lytic cell death morphology including nuclear condensation, large electron-transparent vacuoles and pore formation in the cell membrane, cell swelling and bursting.

We found that, when the NLRP3 inflammasome is activated, two groups of tumor cells could be identified based on LDH release and IL1β secretion. IL1β secretion was highest in PC3 cells and it was one of the lowest in A549 cells. Additionally, in contrast to other tumor types, A549 the levels of multiple secreted cytokines were decreased after LPS/Nigericin treatment compared to untreated controls (**Table 1**). Therefore, we sought to determine the structural differences between these two cell lines after inflammasome modulation using TEM. Human fibroblast cells were used as a normal cell control. Interestingly, VX765 did not affect the amount or shape of the ER in PC3 cells compared to controls, suggesting a lack of functional activity of this organelle (**Figures 5A, D**). In contrast, VX765 increased the granulation of ER in A549 cells, which could indicate that the organelle is functionally active (52) (**Figures 5B, E**). In fibroblasts, VX765 increased the granulation of ER. In addition, VX756 caused the ER cisternae to become expanded, branched, and irregular in shape which could indicate the over synthesis of proteins (53) (**Figures 5C, F**). The structures of the Golgi apparatus were similar in PC3, A549, and fibroblast cells with developed cistern stacks after VX765 treatment suggesting a considerable increase in protein export (54).

Inflammasome activation caused similar ultrastructural changes in ER in PC3, A549 and human fibroblasts cells. After the inflammasome priming with LPS, ER cisternae were expanded in PC3, A549 and human fibroblast cells (**Figures 5G–I**). However, the ultrastructure of the Golgi apparatus was different between PC3 and A549 cells. Unlike in PC3 cells, the Golgi apparatus was poorly developed and weakly visualized in A549 cells, which could indicate impaired protein export (**Figure 5H**). When only Nigericin or LPS/Nigericin was used, ultrastructural signs of non-apoptotic, lytic cell death were identified by TEM in both tumor cell lines. These included nuclear condensation, presence of the multiple rounds electrically transparent vacuoles in the cytoplasm, pore formation in the cell membrane, cell swelling and bursting (**Figures 5J, K, M, N**). In fibroblasts, LPS/Nigericin produced the ultrastructural signs of cell death similar to that in tumor cell lines. However, when used alone Nigericin had a limited effect on human fibroblasts, where few free ribosomes and many polyribosomes were identified in the cytoplasm by TEM suggesting that cells are alive rather than dead (**Figure 5L**).

### The Effect of Targeting NLRP3 Inflammasome on Mitochondria Structure and Function

The effect of VX765, LPS, Nigericin, and LPS/Nigericin on mitochondria structure in A549, PC3, and human fibroblasts was analyzed using TEM. In untreated A549 and PC3 tumor cells, the mitochondrial cristae were poorly defined (**Figures 5A, B**). While VX765 did not affect the ultrastructure of mitochondria in PC3 cells (**Figure 5D**), in A549 cells, the outer membrane and cristae became clearer compared to untreated controls (**Figure 5E**). This could indicate more active ATP synthesis (55–57). In fibroblasts, after VX765 treatment, mitochondria had a condensed matrix lacking distinct cristae compared to untreated controls, suggesting increased mitochondrial activity (55) (**Figures 5C, F**). These data suggest that blocking Caspase 1 function, which is an NLRP3 product, does not affect the mitochondrial activity and tumor cell growth in PC3 cells. In contrast, caspase 1 inhibition induces mitochondrial activity in A549 cells and human fibroblasts.

The matrix of mitochondria was electron-dense in LPS treated PC3 cells, although the majority of cristae were condensed, more of them were clearly visible compared to untreated controls, indicating increased mitochondrial activity (56, 58) (**Figure 5G**). Similarly, in A549 cells and human fibroblasts, some cristae were clearly visible, indicating active ATP synthesis (59) (**Figures 5H, I**). In contrast, cristae were poorly visualized in Nigericin-treated PC3 and A549 cells, suggesting decreased mitochondrial function (60) (**Figures 5J, K**). LPS/Nigericin treatment resulted in poor visualization of cristae in PC3 and A549 cell lines, indicating reduced mitochondrial function (56) (**Figures 5M, N**). Unlike in tumor cells, Nigericin and LPS/Nigericin produced better-defined cristae in fibroblast mitochondria compared to untreated controls, suggesting an increased mitochondrial function (56) (**Figure 5L**).

**Figure 6:**
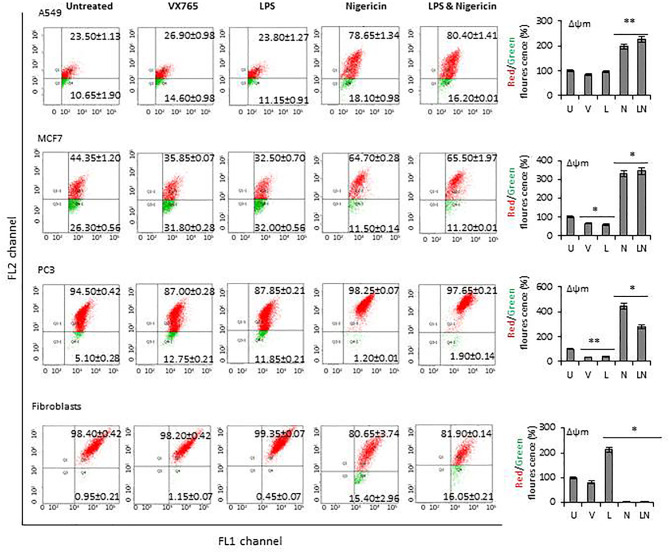
The effect of NLRP3 inflammasome modulation on Δψm in tumor cell lines and human fibroblasts. Nigericin (20 µM, Invivogen) treatment for 24 h with and without 3 h pre-incubation with LPS (1 µg/ml, Sigma, St. Louis, USA) was used to activate the NLRP3 inflammasome. To inhibit Caspase 1, cells were treated with VX765 (20 µM, Invivogen). **U**, Untreated; **V**, VX765; **L**, LPS; **N**, Nigericin; **LN**, LPS/Nigericin. *P < 0.001, **P < 0.05.

The modulation of NLRP3 activity on mitochondrial function was also confirmed by measuring the mitochondrial membrane potential (Δψm) using flow cytometry analysis of JC-1 staining. The effect of NLRP3 modulation on Δψm in A549, MCF7 and PC3 cell lines derived from epithelial tumors and fibroblasts is shown in **Figure 6**. Δψm changes induced by NLRP3 modulation in SH-SY5Y and U138MG cell lines, derived from neuronal tumors are shown in **Supplementary Figure 5**. Statistical analysis of Δψm changes after VX765, LPS, Nigericin and LPS&Nigericin treatments of all cell lines is summarized in **Supplementary Table 5**. In PC3 and MCF7 cell lines as well as human fibroblasts, VX765 slightly decreased the right-shift of the green fluorescence indicating a reduction of the Δψm. In contrast, there were no changes in the right-shift of the green fluorescence in VX765 treated A549 cells, suggesting a lack of effect on Δψm. (**Figure 6**, **Supplementary Table 5**). Interestingly, in neuronal tumors, U138MG and SH-SY5Y, VX765 increased the right-shift of the green fluorescence indicating an increased Δψm, compared to untreated controls (**Supplementary Figure 5**, **Supplementary Table 5**). LPS slightly decreased the Δψm in all tumor cells; however, in human fibroblasts, LPS increased Δψm compared to untreated controls (**Figure 6**, **Supplementary Table 5**). Nigericin and LPS/Nigericin treatment increased Δψm in all tumor cells compared to untreated cells (**Figure 6**, **Supplementary Figure 5**, **Supplementary Table 5**). In contrast, Nigericin and LPS/Nigericin treatment decreased Δψm in human fibroblasts compared to untreated controls (**Figure 6**, **Supplementary Table 5**). These data indicate that independent of the first stimulus, the second stimulus increases the mitochondria membrane potential and triggers mitochondrial dysregulation, which could cause tumor cell death. Interestingly, NLRP3 activation-induced cell death of fibroblasts requires both the first and second stimuli.

### Suppressing Caspase 1 Activity with VX765 Stimulates Angiogenesis

**Figure 7:**
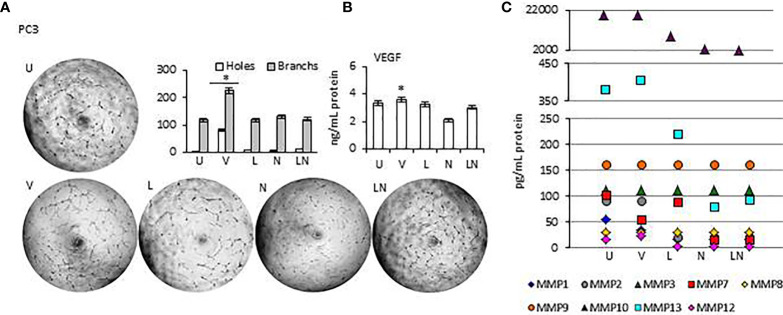
The effects of releasing cytokines from PC3 cells after inhibition or stimulation of the NLRP3 inflammasome on the HUVEC tube formation. Nigericin (20 µM) treatment for 24 h with and without 3 h pre-incubation with LPS (1 µg/ml) was used to activate the NLRP3 inflammasome. To inhibit Caspase 1, cells were treated with VX765 (20 µM). **U**, Untreated; **V**, VX765; **L**, LPS; **N**, Nigericin; **LN**, LPS/Nigericin. *p<0.05, n=3.

Tumor growth is supported by neovasculogenesis, which is regulated by cytokines released from tumors (61). Therefore, we sought to determine whether modulation of NLRP3 activity could induce endothelial cell tube formation *in vitro*, indicating activation of angiogenesis (62). Supernatants of tumors were collected at 24 h and used for the HUVEC tube formation assay. HUVECs were maintained in a culture medium from each tumor cell line after NLRP3 modulation. In addition, the level of proangiogenic VEGF and Matrix metalloproteinases (MMPs) in tumor cells conditioned culture medium was analyzed. We found that in all tumor cell lines, the level of VEGF and MMP13 in culture medium was significantly higher after treatment with VX765 compared to Nigericin and LPS/Nigericin and untreated controls (p < 0.05) (**Figure 7**, **Supplementary Figures 6, 7**, **Supplementary Tables 6, 7**). Additionally, VX765 increased the release of MMP2, MMP7, and MMP10 in A549 cells, while the secretion of MMP7, MMP10, and MMP12 was increased only in PC3 cells (**Figure 7**, **Supplementary Figure 6**, **Supplementary Tables 6** and **7**). The level of several MMPs, including MMP2, MMP3, MMP7, MMP10, and MMP12 was significantly higher in U138MG conditioned culture medium (p < 0.05) (**Figure 7**, **Supplementary Figure 7**, **Supplementary Tables 6** and **7**). In fibroblasts, VX765 increased the release of VEGF, MMP2, MMP3, MMP10, and MMP12 compared to untreated controls, while MMP3 secretion was below the detection level (**Supplementary Figure 6**, **Supplementary Tables 6** and **7**). Consistent with the VEGF and MMPs data, VX765 culture medium induced the assembly of tubular structures by the HUVECs compared to that in controls, and Nigericin or LPS/Nigericin treated groups (p < 0.05) (**Figure 7**, **Supplementary Figures 6** and **7**, **Supplementary Tables 6** and **7**).

With exception of SH-SY5Y cells, the release of VEGF was significantly decreased in Nigericin and LPS/Nigericin treated conditioned culture medium from all tumor types (A549, MCF7, PC3, and U138MG) and fibroblasts compared to VX765 and untreated controls. Accordingly, HUVECs maintained in medium conditioned by cells treated with Nigericin and LPS/Nigericin formed fewer tubular structures compared to VX765 treated conditioned cell culture medium. The difference in tube formation between HUVECs treated with conditioned culture medium from each tumor cell line was not significant (p < 0.05). The effect of conditioned culture medium from PC3 cells, expressing the highest NLRP3 level upon treatment with LPS/Nigericin, on HUVECs tube formation is shown in **Figure 7**. The effect of culture medium from A549 and MCF7 cells as well as fibroblasts is shown in **Supplementary Figure 6**. Also, the effect of culture medium from neuronal tumor cells, U138MG and SH-SY5Y, on HUVEC tube formation is shown in **Supplementary Figure 7**. Statistical analysis of HUVEC tube formation after incubation with conditioned culture medium from VX765, LPS, Nigericin, and LPS/Nigericin treated cell lines is shown in **Supplementary Table 8**.

## Dıscussıon

The main product of the NLRP3 inflammasome is active caspase 1, which is produced by cleavage of the Pro-Caspase 1 (63). Caspase 1 releases the functional IL-1β, which is a pleiotropic cytokine inducing fever, activating and recruiting immune cells into the inflamed tissue (64). In this study, we investigated the effect of an inducer of the NLRP3 inflammasome, Nigericin and an inhibitor of Caspase 1, VX765, on tumor progression in a panel of cancer cells, including A549 (lung cancer), MCF7 (breast cancer), PC3 (prostate cancer), SH-SY5Y (neuroblastoma) and U138MG (glioblastoma) cell lines. We also used fibroblasts as a non-malignant cell control.

### The Effect of NLRP3 Activation With Nigericin

NLRP3 complex formation requires two stimuli: priming (LPS) and activation (Nigericin) (37). LPS binds to pattern recognition receptors (PRRs) leading to the nuclear translocation of nuclear factor-κB (NF-κB) and increased transcription of *IL1-β* and *IL-18* (65). However, a second stimulus is required to initiate the functional inflammasome formation (66). Therefore, we analyzed the effect of LPS and Nigericin on NLRP3 activation individually and in combination. Interestingly, our results revealed that priming of tumor cells with LPS increased the release of IL1-β and IL-18 without the requirement for the second stimulus (Nigericin). In contrast, priming of fibroblasts with LPS alone failed to release IL1-β and IL-18 without treatment with Nigericin as a second stimulus. In addition to potassium efflux, NLRP3 can be activated by various stimuli, such as ATP, toxins, reactive oxygen species (ROS), hypoxia, and mitochondrial dysfunction (67, 68). Therefore, our results suggest that LPS could activate NLRP3 and cause the release of IL1-β and IL-18 without a second stimulus provided by Nigericin. In tumor cells there could be other second stimuli already present, negating the requirement for Nigericin. Such stimuli could include oncogene-induced ROS (69).

Nigericin without LPS priming also activated NLRP3 evidenced by increased IL1-β and IL-18 expression. It was recently shown that TLRs, FAS-associated death domain protein and IL-1R ligands can act as the NLRP3 priming stimuli (37, 70, 71). Tarassishin and colleagues demonstrated that, in glioblastoma cells, the priming signal can be provided by IL-1 which can be produced by tumor cells in large quantities (72). Interestingly, after combined LPS/Nigericin treatment, tumor cells demonstrated two distinct patterns of NLRP3 inflammasome activation. While the release of IL-1β and IL-18 was higher in PC3 and U138MG cells compared to controls, these cytokines levels were lower in A549, MCF7 and SH-SY5Y cells. Microenvironment (73), redox balance (74, 75) and osmolarity (76) of cells may influence the release of IL-1β from different tumor types (77). However, little is known about the mechanism of IL-1β release upon LPS/Nigericin treatment. Our data suggest that NLRP3 activation through LPS/Nigericin treatment leads to a high level of IL-1β and IL-18 secretion in prostate cancer and glioblastoma cell lines compared to cell lines derived from lung cancer, breast cancer, and neuroblastoma.

Nigericin decreased cell viability and proliferation in all tumor cell lines investigated, both as monolayers and in sphere culture. Nigericin increases the efflux of K^+^ and increases the intracellular Ca^2+^ concentration (78). According to Katsnelson and colleagues, this rapid decrease in cytosolic K^+^ is a sufficient stimulus for initiation of the NLRP3 inflammasome cascade, independent of cytosolic Ca^2+^ levels (78). However, high intracellular Ca^2+^ can trigger apoptotic and non-apoptotic programmed cell death *via* caspase cascades (79). In our study, Nigericin and LPS/Nigericin increased the released LDH in tumor cells compared to controls, suggesting induction of non-apoptotic cell death. Although we suggest non-apoptotic cell death, we could not exclude pyroptosis, an inflammatory programmed cell death (80). Pyroptosis is characterized by rapidly formed membrane pores, membrane rupture, cell swelling and release of intracellular content into the extracellular space, including cytosolic proteins such as LDH (81). Opened membrane pores in pyroptotic cells permit both, Annexin V and impermanent dyes such as Propidium Iodide (PI), to enter the cell and localize in the inner membrane [39]. Therefore, in contrast to apoptosis, pyroptotic cells appear positive for both, Annexin V and PI. Supporting pyroptosis as the mechanism of cell death we identified that Nigericin and LPS/Nigericin treated tumor cells, were Annexin V and PI positive.

Additionally, Nigericin increased Δψm in all tumor cell lines investigated in this study. The apoptotic effect of elevated Δψm was demonstrated by Vander Heiden MG and colleagues (82); however, Heerdt and colleagues also showed that Nigericin induced Δψm is not associated with increased mitochondria-associated cytochrome-c release (83). Additionally, disrupted Δψm and reactive oxygen species were linked to pyroptosis in macrophages (84). Further support that pyroptosis was the mechanism of cell death, we found signs of oncolytic cell death in cells treated with Nigericin and LPS/Nigericin in TEM. It appears that pyroptosis in this context is a unique form of cell death induced in tumor cells as we found no decrease in cell viability and proliferation of non-tumor cells, fibroblasts. Moreover, the effect of Nigericin on mitochondria was also different in tumors compared with fibroblasts, where the reduction of Δψm in fibroblasts and mitochondrial damage was negligible. These data suggest that tumor cells are more susceptible to Nigericin induced pyroptosis compared to fibroblasts.

Interestingly, while combined LPS/Nigericin treatment reduced cell proliferation in all tumor cell line monolayers, its effect on tumorsphere formation was less uniform. While combined LPS/Nigericin treatment reduced sphere formation in MCF7 and SH-SY5Y cell lines, which also had the lowest release of IL-1β, in PC3 and U138MG cells, where the release of IL-1β was the highest, treatment increased the size of tumorspheres. It was previously demonstrated that IL-1β may promote tumor growth and invasion through activation of cancer stem cell self-renewal (85). Although monolayers are useful tools for functional tests, cell-cell and cell-extracellular environment interactions which are responsible for cell differentiation, proliferation, vitality, responsiveness to stimuli and drug metabolism cannot be represented in monolayers as they would be in the tumor mass (86). Due to disturbances in interactions with the microenvironment, tumor cell lines growing adherently can lose their polarity. It results in changes in the response of those cells to various cellular signaling events which may include to stimulation of the NLRP3 inflammasome. On the other hand, spheres more likely mimic the physical and biochemical features of a solid tumor mass due to the proper cell-cell and cell-environment interactions (87). In sphere models, cell proliferation depends on many factors such as the location of cells, presence of initiating cancer stem cells and the level of hypoxia (86). In our study, after LPS/Nigericin treatment, the growth pattern of PC3 and U138MG cells were different in monolayer and spheres. It may be caused by pathophysiological differences between tumorspheres due to the level of inflammasome induced hypoxia which can trigger cancer stem cell self-renewal in U138MG and PC3 spheres (88). Studies demonstrated that IL-1β induced hypoxia in glioblastoma and modulates the tumor progression by interacting directly with the tumor cells (89). Our data suggest that the effect of Nigericin on tumor survival will depend on the balance between pyroptosis and survival associated with IL-1β release. Interestingly, pyroptosis could disrupt cell integrity and release the intracellular content including IL-1β (90). Therefore, we suggest that pyroptosis could modify the tumor microenvironment establishing a chronic inflammatory milieu by releasing pro-inflammatory cytokines (91, 92). This pro-inflammatory tumor microenvironment could also be supported by LPS, which is the priming signal in inflammasome formation (93). LPS, as a PAMP, can activate tumor-associated macrophages and trigger the release of inflammatory cytokines including TNFα, IFNγ, IL-2, and IL-4 (94–96). Additionally, LPS can stimulate IL-6 production by tumor fibroblasts (97). Our data demonstrated that LPS induced the secreted of IL-6, as well as 31 cytokines in fibroblasts. Interestingly, in contrast to fibroblasts, fewer cytokines had increased secretion; secretion of only one cytokine in A549 and MCF7 cells, 5 cytokines in SH-SY5Y cells significantly increased after LPS treatment as compared to untreated cells. In addition, LPS did not affect the cytokine secretion pattern of PC3 and U138MG cells compared to untreated control. We did not identify a common pattern of cytokine secretion changes in five different tumor cell lines induced by LPS treatment. We propose that the differences in the level of NLRP3 activation in tumor cell lines are related to variations in the inflammatory nature of the individual tumor types, where pro-inflammatory cytokines produced by the tumor could act as priming agents, instead of LPS (72, 98–100).

In contrast to LPS (where no common cytokine could be identified activated in tumor cells line), Nigericin treatment revealed MIF as the most consistently upregulated cytokine in tumor cells, except for MCF7 cells. It appears that MIF could function as positive feedback to potentiate Nigericin activation of NLRP3, as this cytokine regulates inflammasome assembly and activation (101). It also was shown that MIF is required for the NLRP3–vimentin interaction, which is essential for IL-1β and IL-18 secretion (101). Our data demonstrate that MIF production could be involved in NLRP3 activation in tumor cells treated with Nigericin. Interestingly, Nigericin did not induce the production of MIF in MCF7 breast cancer cells; instead, these cells produced CXCL8 and CCL13. Nigericin can mimic the P2X7 receptor and trigger the second stage of NLRP3 inflammasome activation (9). It was demonstrated that P2X7 receptors were involved in the production of CXCL8 in human bronchial epithelial cells (102). Additionally, the release of CXCL8 was observed together with IL-1β in cigarette smoker-associated chronic obstructive pulmonary disease (COPD) patients (102). It was reported that IL-1β induces CXCL8 *via* NLRP3 (102). Therefore, we suggest that Nigericin may also lead to the production of CXCL8 in MCF7 cells. However, the role of CCL13 in the regulation of the NLRP3 inflammasome in MCF7 cells requires further investigation.

### The Effect of VX765 on Inflammasome Function

VX765 is a selective inhibitor of Caspase 1, a major product and effector of the NLRP3 inflammasome (35). To confirm VX765 inhibition of Caspase 1, cells were treated with LPS/Nigericin, which activates NLRP3 to produce the active caspase. In all tumor cell lines and fibroblasts, Caspase 1 inhibition, partially attenuated the effects of LPS/Nigericin. In contrast to NLRP3 activation, Caspase 1 inhibition alone did not affect cell proliferation, death and LDH release. Moreover, TEM data suggest that VX765 improved the cell metabolism and mitochondrial functions in A549, PC3, and fibroblasts. Studies demonstrated that ER stress (ERS) activates the NLRP3 inflammasome and triggers mitochondrial damage. ERS increases ROS and promotes translocation of the inflammasome to the mitochondria. NLRP3 is involved in the ERS-induced cleavage of caspases including caspase-1 leading to mitochondrial damage, which is required for the production of mature IL-1β (103, 104). During ERS, the capacity of the ER to fold proteins becomes saturated by impaired protein glycosylation or disulfide bond formation (105). According to our findings, it appears that the inhibitor function VX765 to caspase-1 results with decrease in ERS and increase in the capacity of ER to fold proteins accurately. Caspase 1 inhibition also increased production of CCL24 in epithelial tumors such as lung, breast and prostate. In contrast to epithelial tumors (A549, MCF7 and PC3), Caspase 1 inhibition triggered the release of CCL11 and CCL26 in a neuroblastoma cell line. These cytokines are ligands for CCR3, which is expressed on the surface of immune and non-immune cells and promotes cell migration and proliferation (40, 106, 107). It was previously demonstrated that the CCL24-CCR3 interaction increases eosinophil adhesion and facilitates migration and infiltration of eosinophils (108). Infiltrated eosinophils were shown to increase tumor cell viability and their proangiogenic potential (109). CCL24 can also promote angiogenesis *via* the RhoB-VEGFA-VEGFR2 signaling pathway and contributing to malignancy (110). Additionally, CCL11-CCR3 interaction promotes cell migration and proliferation (110). Our data demonstrated that Caspase 1 inhibition induces the release of VEGF and MMPs from tumor cells and stimulates endothelial cell tube formation. This is further supported by our data on decreased cell death in tumor cells when Caspase 1 was inhibited. Our data is also corroborated Lopez-Pastrana and colleagues’ findings, who demonstrated that inhibition of Caspase 1 reduces pyroptosis and stimulates endothelial cell survival, which is mediated by VEGFR-2 signaling (111). Altogether our data suggest that inhibiting Caspase 1 by VX765 induces angiogenesis.

In conclusion, in this study, we, for the first time, analyzed the effect of an inducer (Nigericin) of NLRP3 and inhibitor of Caspase 1 (VX765) in a panel of different tumor types and normal fibroblast controls. We identified that the degree of inflammasome activation varies across the investigated tumor cell lines. Upon LPS/Nigericin treatment, activation of NLRP3 was the highest in prostate cancer and glioblastoma cells, whilst it was the lowest in the neuroblastoma cell line, SH-SY5Y. Additionally, for the first time, we have demonstrated that cell death caused by LPS/Nigericin treatment, produced variable effects on tumor cells, depending upon their NLRP3 activation level and cytokines released. In this study, we used only one cell line for each tumor type. Considering the heterogeneous nature of every tumor type, the efficacy of the inflammasome activation could further vary in different tumor cells derived from the same cancer type. Additionally, although inflammasome targeting is used as a therapeutic approach in many diseases, our study showed that inhibition of NLRP3 may not be the best approach for the treatment of some cancers. This is because the inhibiting of Caspase 1 activity using VX765 could stimulate the angiogenesis by releasing CCL24, CCL11, and CCL26 cytokines and protecting cell viability in some tumors. Therefore, targeting inflammasomes for cancer treatment will require prior testing of the effect of inflammasome reactivation in that tumor. Future in-vivo investigations will better clarify the efficiency of inflammasome activation and Caspase-1 inhibition on tumor progression. However, according to our current findings, inflammasomes could be excellent targets for personalized cancer treatment, as analysis of inflammasome activation status and potential outcome of their targeting in each patient could identify a supplemental strategy to control tumor growth if appropriate biomarkers can be identified and validated.

## Data AvailabilityStatement

The original contributions presented in the study are included in the article/**Supplementary Material**; further inquiries can be directed to the corresponding author.

## Author Contributions

All authors listed have made a substantial, direct, and intellectual contribution to the work and approved it for publication.

## Conflict of Interest

The authors declare that the research was conducted in the absence of any commercial or financial relationships that could be construed as a potential conflict of interest.
