# Supplementary material for: Therapeutic Potential of Pharmacological Targeting NLRP3 Inflammasome Complex in Cancer
Source: Front Immunol. 2021 Feb 3;11:607881. doi: 10.3389/fimmu.2020.607881 (PMC7887322; doi:10.3389/fimmu.2020.607881)
Supplement: Supplementary file 1 [file DataSheet_1.pdf]

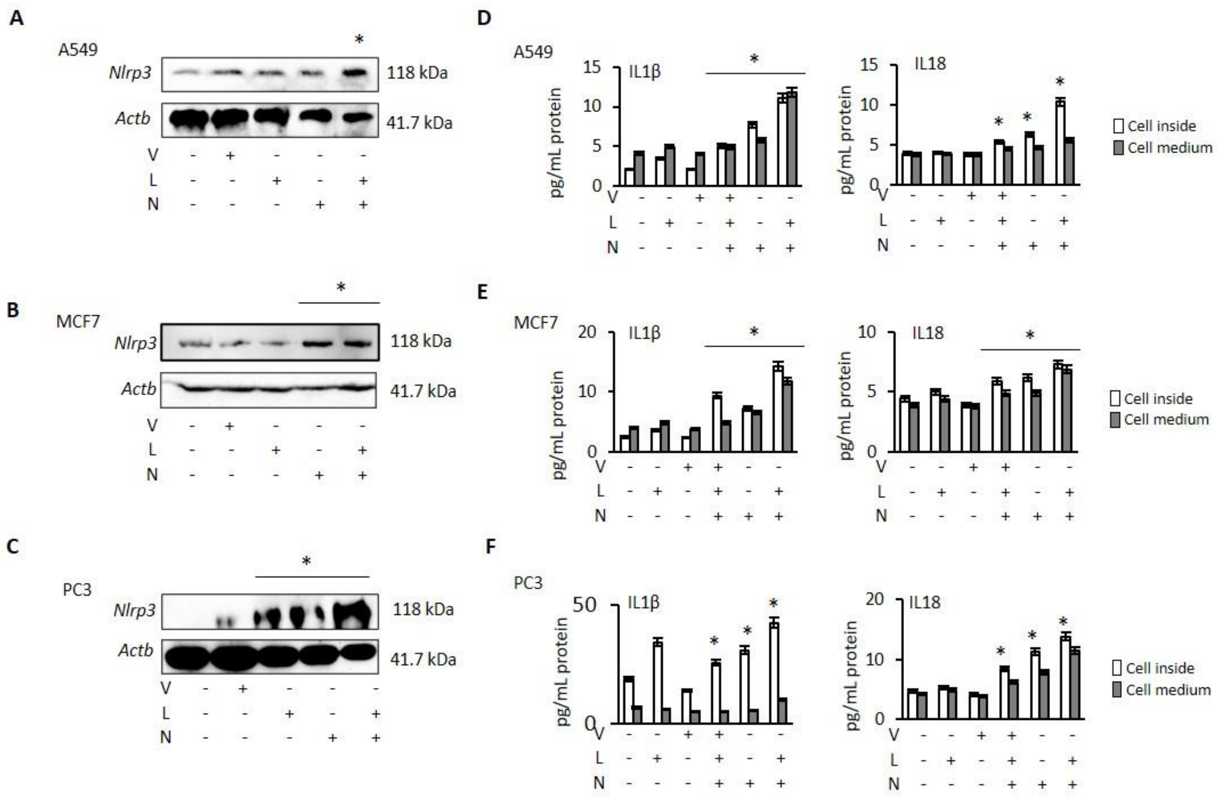

Supp. Fig 1. Inhibition and stimulation of NLRP3 in A549 and MCF7 and PC3 cell lines: Nigericin (20µM, Invivogen) treatment for 24 hours with and without 3 hours pre-incubation with LPS (1µg/ml, Sigma, St. Louis,USA) was used to activate NLRP3 inflammasome. To inhibit Caspase 1, cells were treated with VX765 (20µM, Invivogen). (A-C) NLRP3 protein expression was demonstrated by western blot. (D-F) IL-1β and IL-18 levels were quantified, from inside the cells and in the culture medium representing levels secreted from the cells, by ELISA. U: Untreated, V: VX765, L: LPS, N: Nigericin, LN: LPS/Nigericin. (*$p<0.05$, n=3)

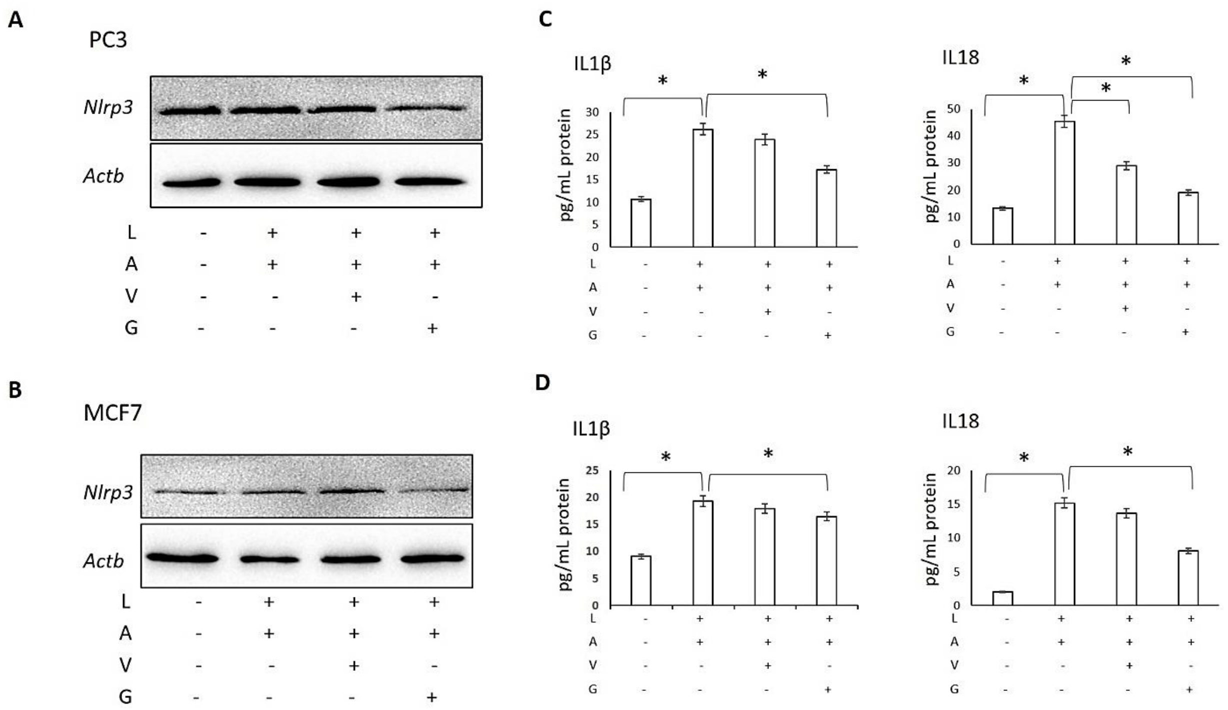

Supp. Fig 2. The effect of ATP on stimulation of NLRP3 in PC3 and MCF7 cell lines: ATP (3mM, Sigma) treatment for 1,5 hours with and without 3 hours pre-incubation with LPS (1µg/ml, Sigma, St. Louis, USA) was used to activate the NLRP3 inflammasome. To inhibit Caspase 1, LPS and ATP treated cells were co-treated with VX765 (20µM, Invivogen) or Glybenclamide (25µg/mL, Invivogen). (A-B) NLRP3 protein expression was demonstrated by western blot. (C-D) IL-1β and IL-18 levels were quantified, from the culture medium representing levels secreted from the cells, by ELISA. U: Untreated, L: LPS, A: ATP, V: VX765, G: Glibenclamide (*p<0.05, n=3).

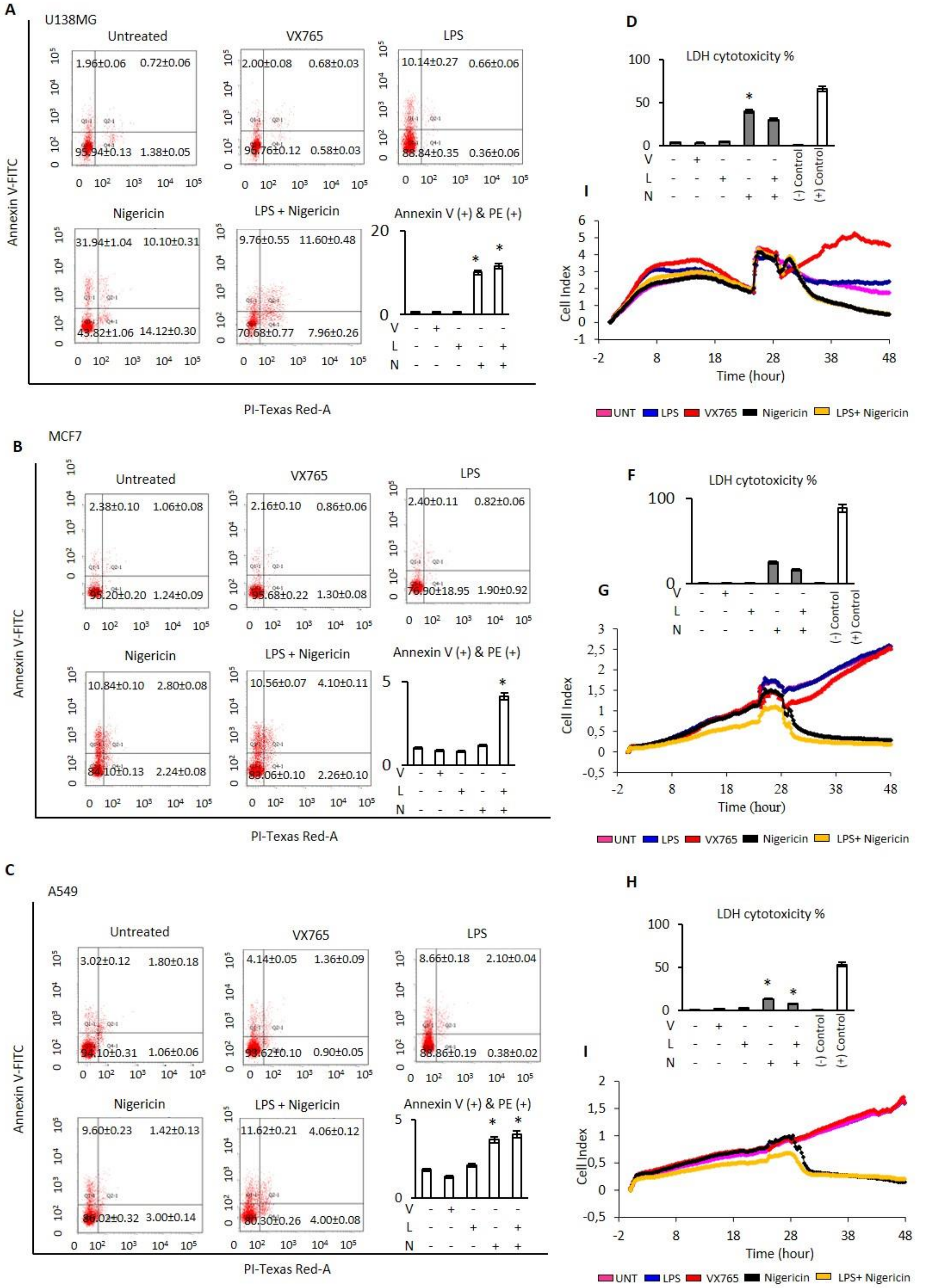

Supp. Fig 3. Effect of NLRP3 inhibition and stimulation on cell proliferation and viability of U138MG, MCF7 and A549 cell lines. Nigericin (20µM, Invivogen) treatment for 24 hours with and without 3 hours pre-incubation with LPS (1µg/ml, Sigma, St. Louis,USA) was used to activate NLRP3 inflammasome. To inhibit Caspase 1, cells were treated with VX765 (20µM, Invivogen). (A-C) Expression of Annexin V (D, F, H) LDH cytotoxicity assay and (E, G, I) a real time cell proliferation assay. U: Untreated, V: VX765, L: LPS, N: Nigericin, LN: LPS/Nigericin. *p<0.05, n=3.

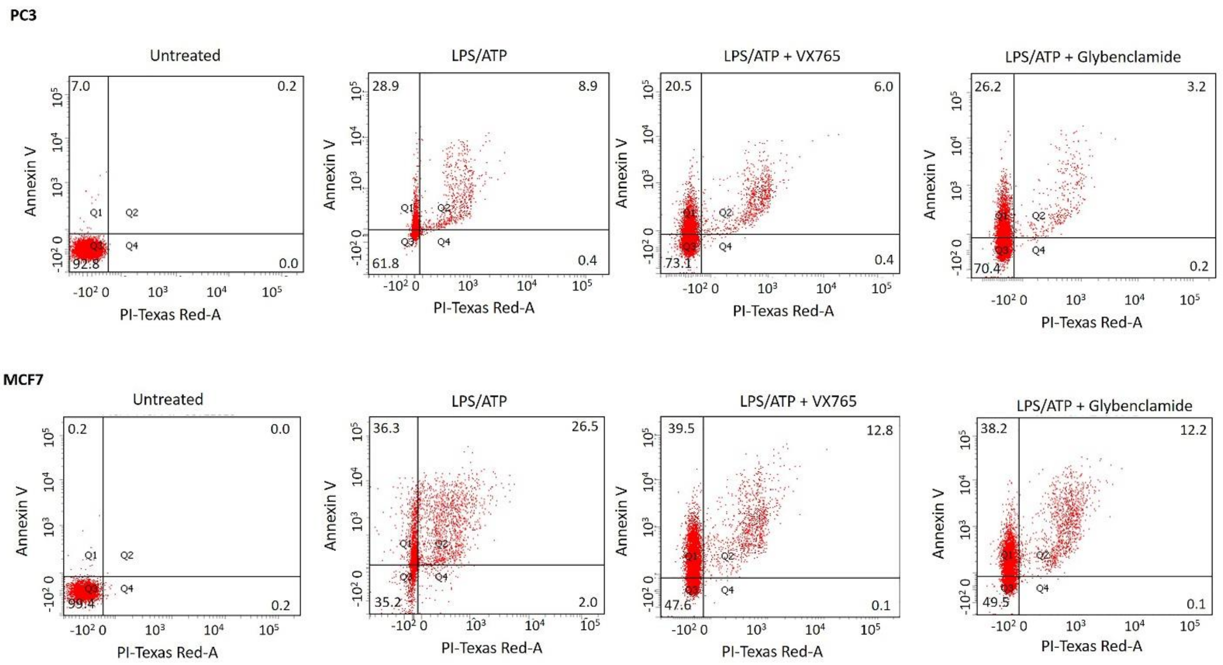

Supp. Fig 4. Effect of LPS/ATP mediated NLRP3 stimulation and inhibition of LPS/ATP mediated NLRP3 stimulation on cell viability in PC3 and MCF7 cell lines. ATP (3mM, Sigma) treatment for 1,5 hours with and without 3 hours pre-incubation with LPS (1µg/ml, Sigma, St. Louis, USA) was used to activate the NLRP3 inflammasome. To inhibit NLRP3 or Caspase 1, LPS and ATP treated cells were co-treated with VX765 (20µM, Invivogen) or Glibenclamide (25µg/mL, Invivogen), respectively.

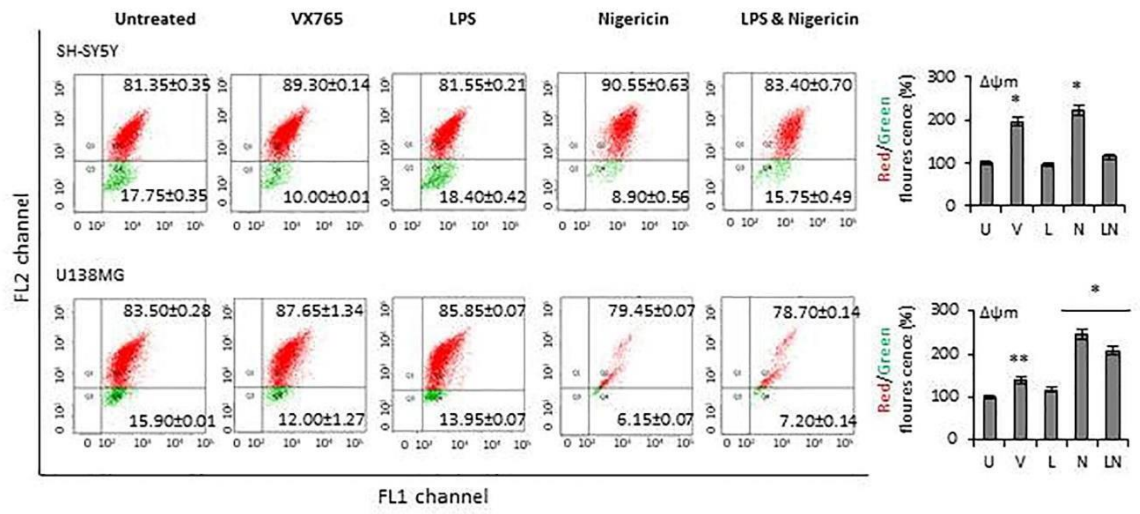

Supp. Fig 5. The effect of stimulation and suppression of NLRP3 inflammasome activation on Δψm in SH-SY5Y and U138MG cell lines. Nigericin (20µM, Invivogen) treatment for 24 hours with and without 3 hours pre-incubation with LPS (1µg/ml, Sigma, St. Louis,USA) was used to activate NLRP3 inflammasome. To inhibit Caspase 1, cells were treated with VX765 (20µM, Invivogen). **U**: Untreated, **V**: VX765, **L**: LPS, **N**: Nigericin, **LN**: LPS&Nigericin; *: P<0.001, **:P<0.05 n=3.

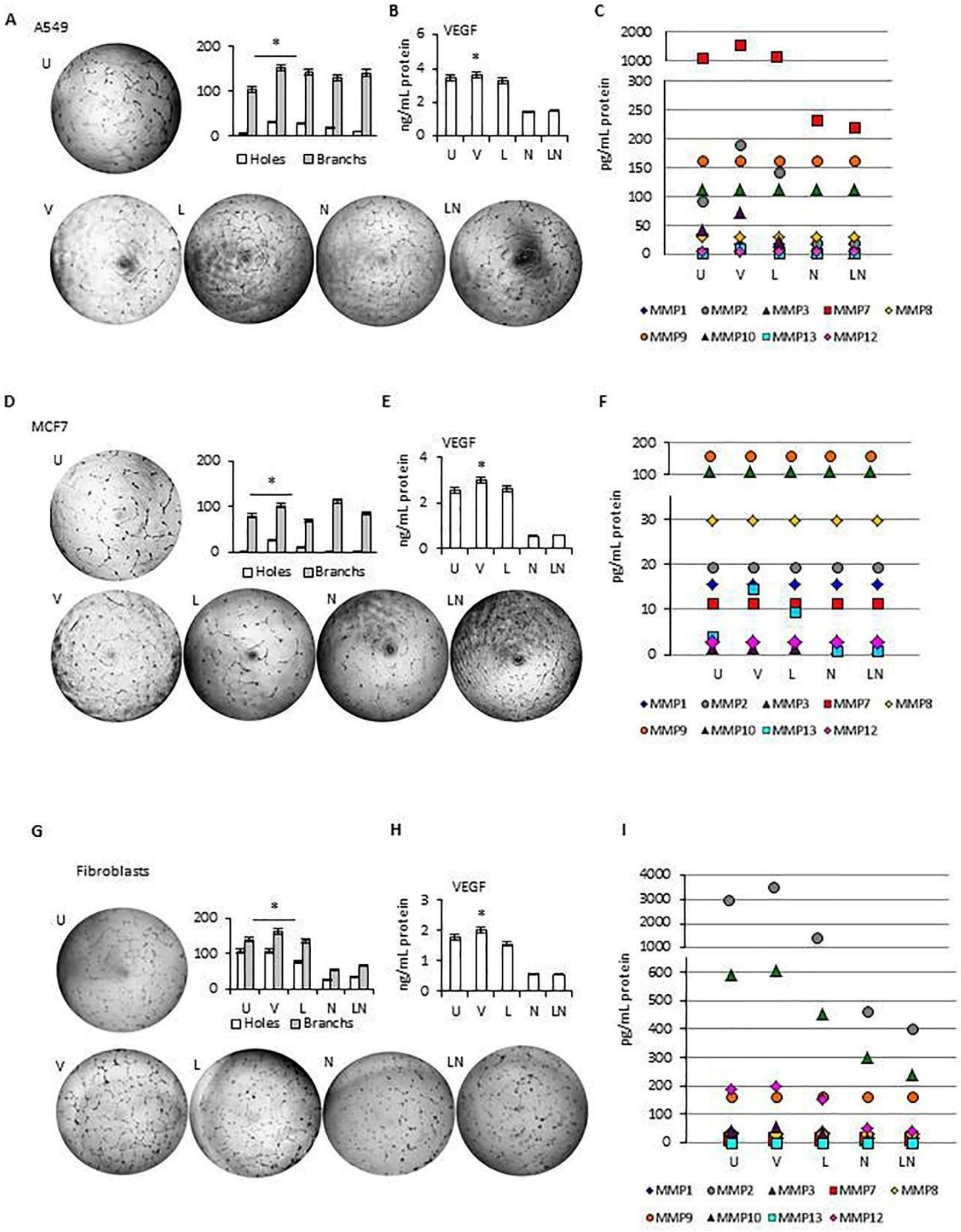

Supp. Fig 6. The effect of releasing cytokines from A549 and MCF7 cell lines and fibroblasts after inhibition or stimulation of the NLRP3 inflammasome on HUVECs tube formation. Nigericin (20µM, Invivogen) treatment for 24 hours with and without 3 hours pre-incubation with LPS (1µg/ml, Sigma, St. Louis,USA) was used to activate NLRP3 inflammasome. To inhibit Caspase 1, cells were treated with VX765 (20µM, Invivogen). **U**: Untreated, **V**: VX765, **L**: LPS, **N**: Nigericin, **LN**: LPS and Nigericin.
*p<0.05, n=3

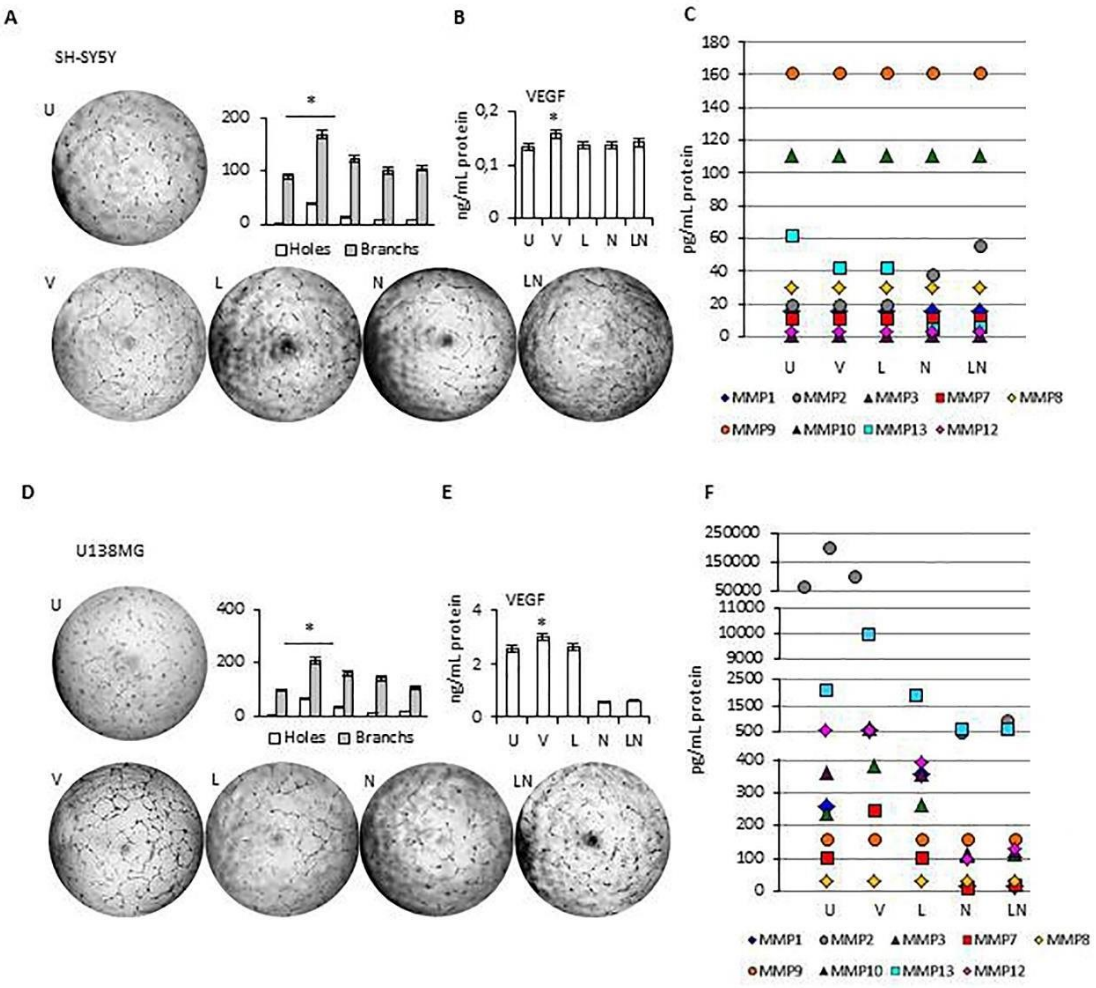

Supp. Fig 7. The effects of releasing cytokines from SH-SY5Y and U138MG cell lines after inhibition or stimulation of the NLRP3 inflammasome on HUVECs tube formation. Nigericin (20µM, Invivogen) treatment for 24 hours with and without 3 hours pre-incubation with LPS (1µg/ml, Sigma, St. Louis,USA) was used to activate NLRP3 inflammasome. To inhibit Caspase 1, cells were treated with VX765 (20µM, Invivogen). **U**: Untreated, **V**: VX765, **L**: LPS, **N**: Nigericin, **LN**: LPS and Nigericin.
*$p<0.05$, n=3

Western Blott membranes

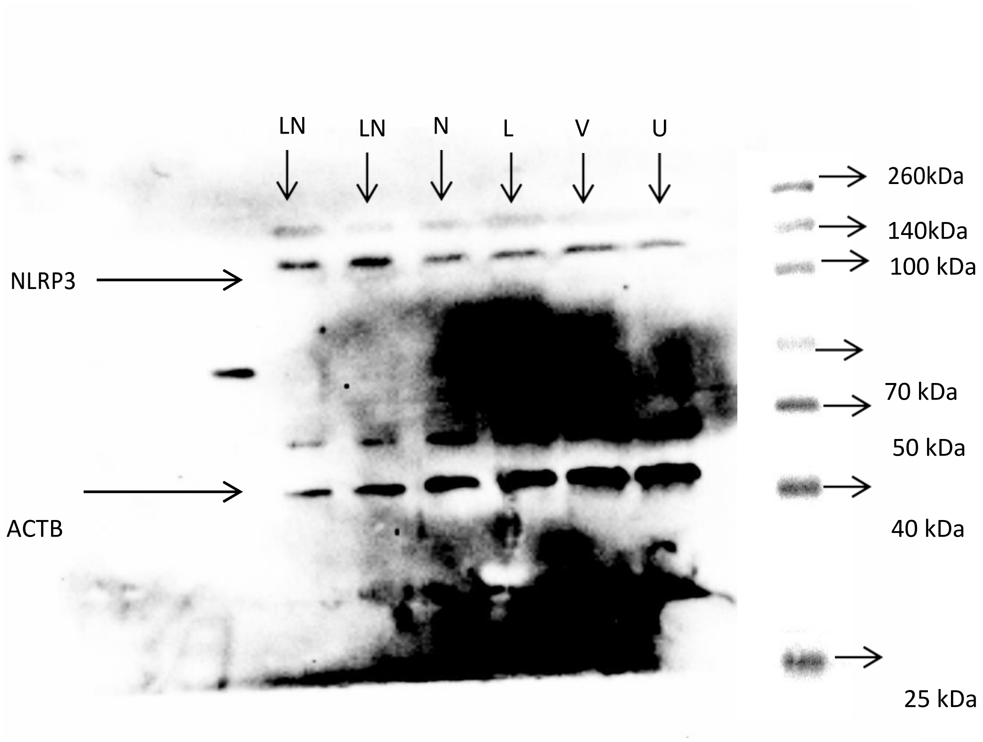

A549: NLRP3 and ACTB

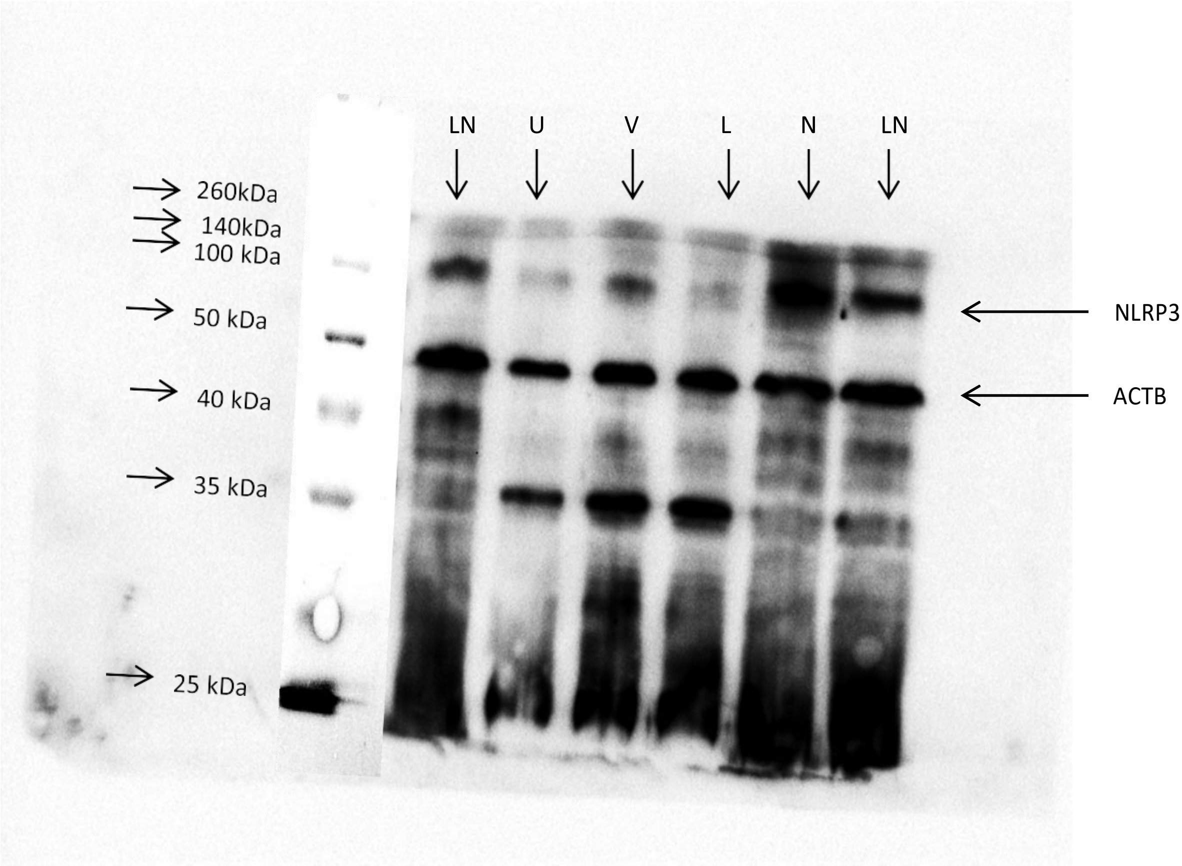

U138MG: NLRP3 and ACTB

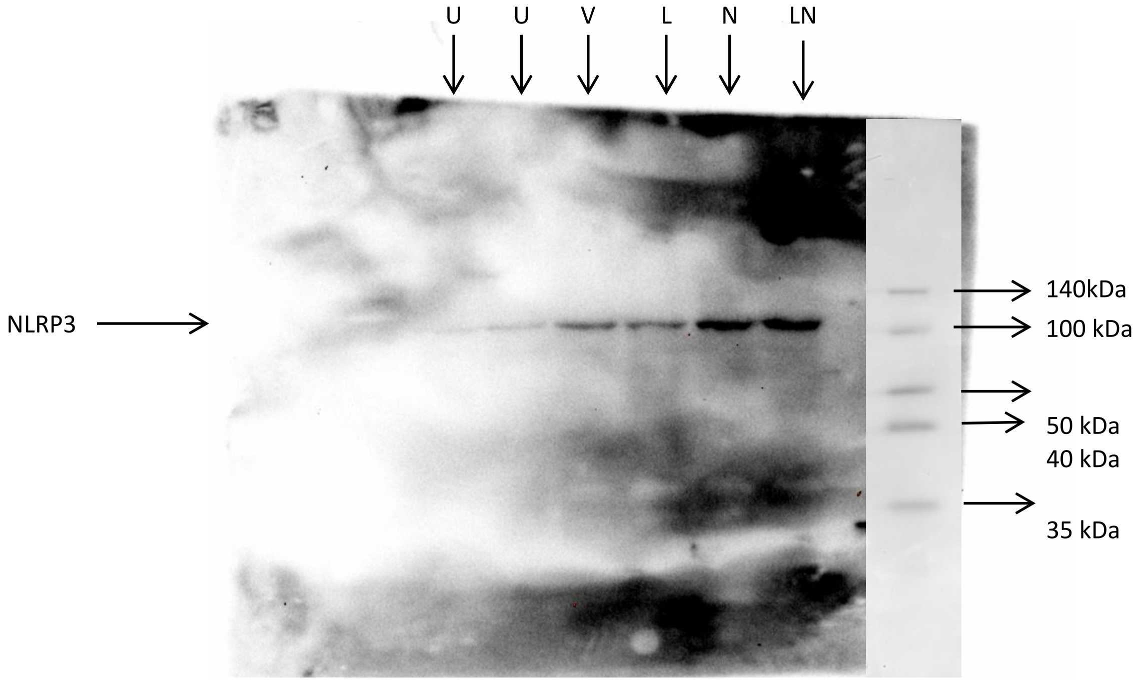

SH-SY5Y: NLRP3

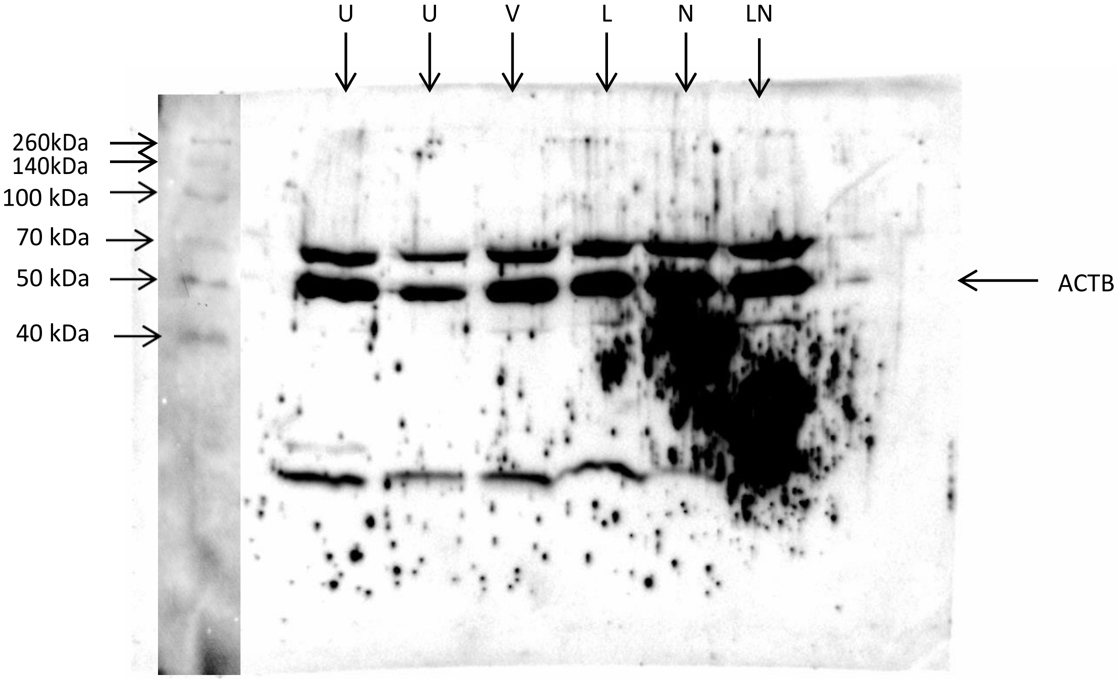

SH-SY5Y: ACTB

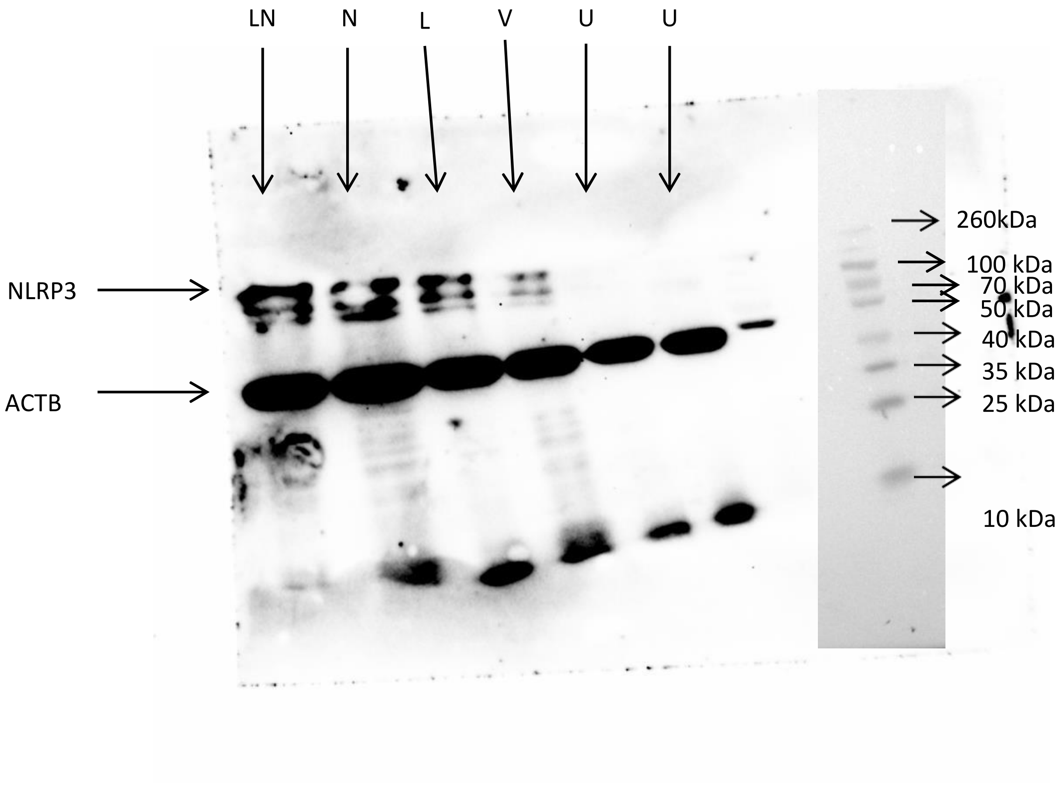

PC3: NLRP3 and ACTB

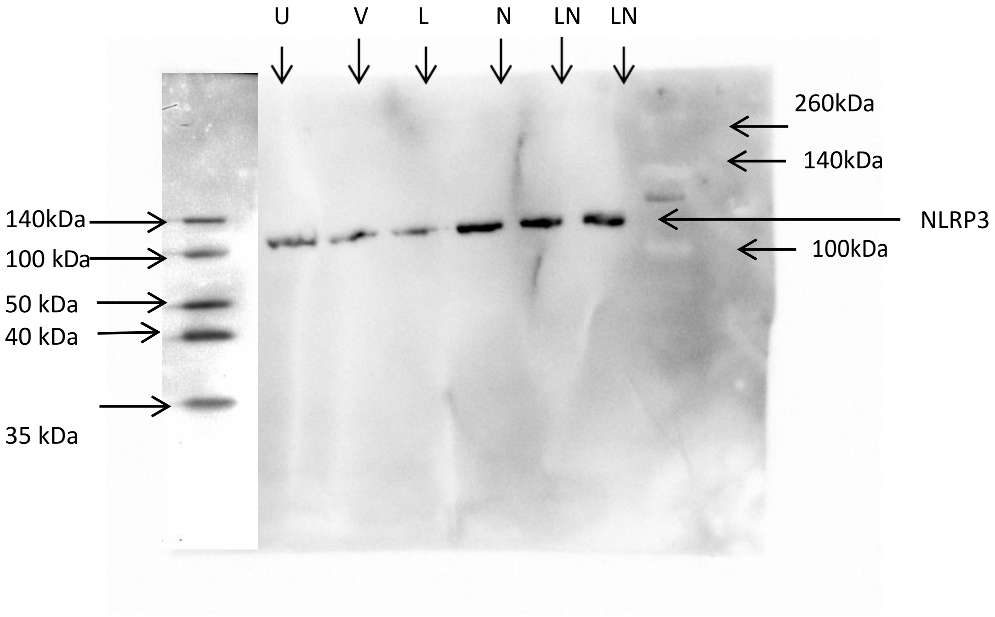

MCF7: NLRP3

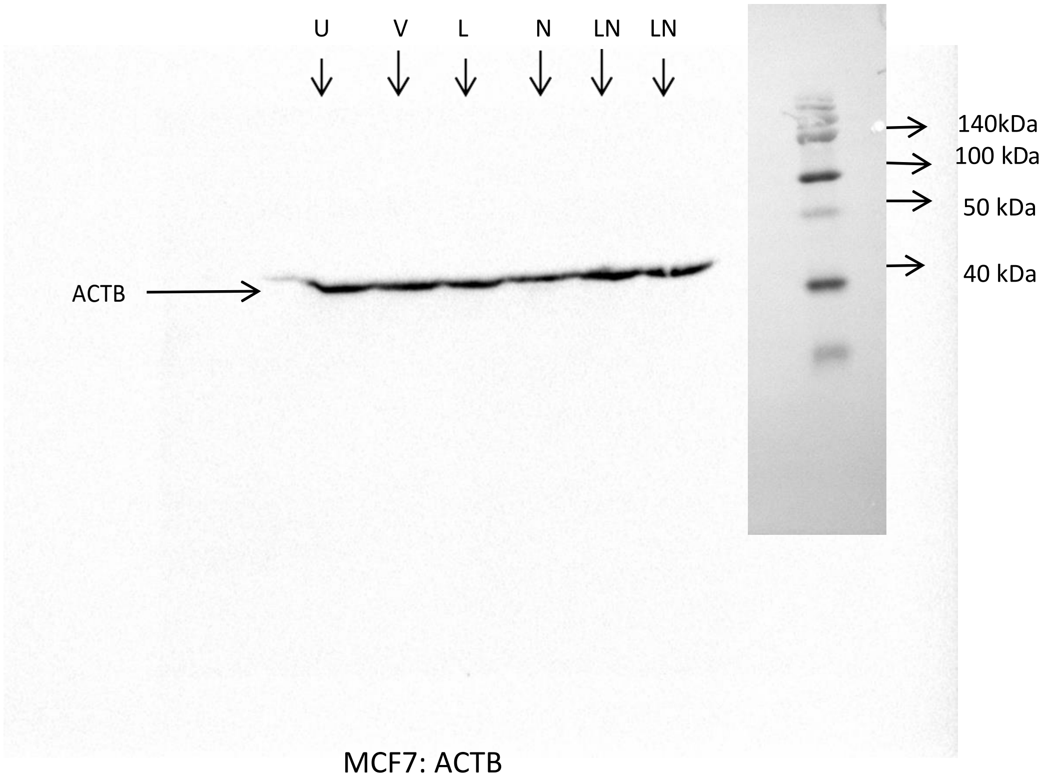

MCF7: ACTB

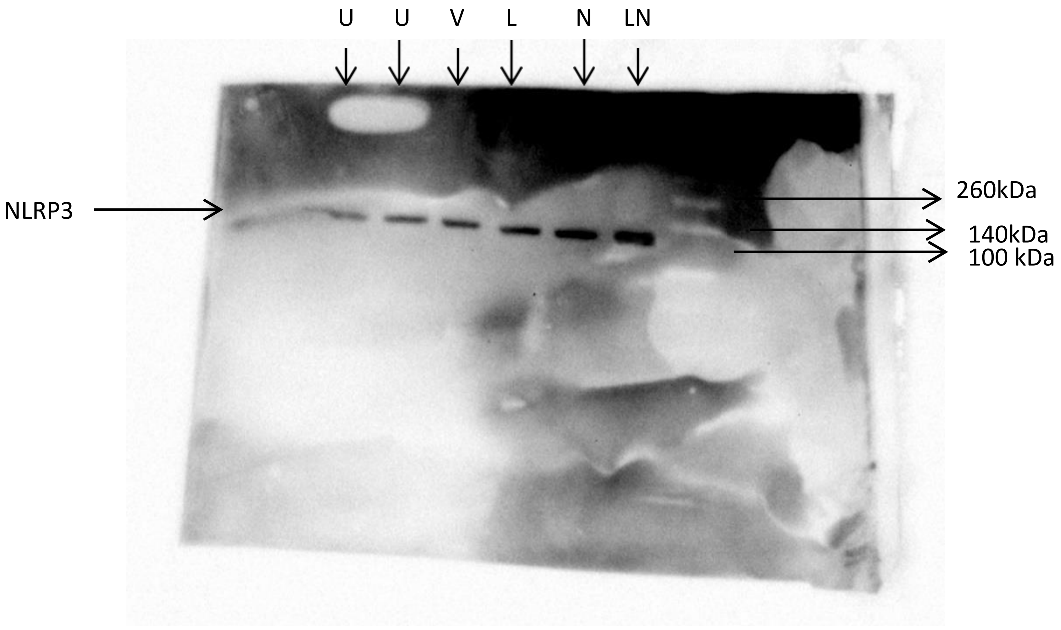

Fibroblasts: NLRP3

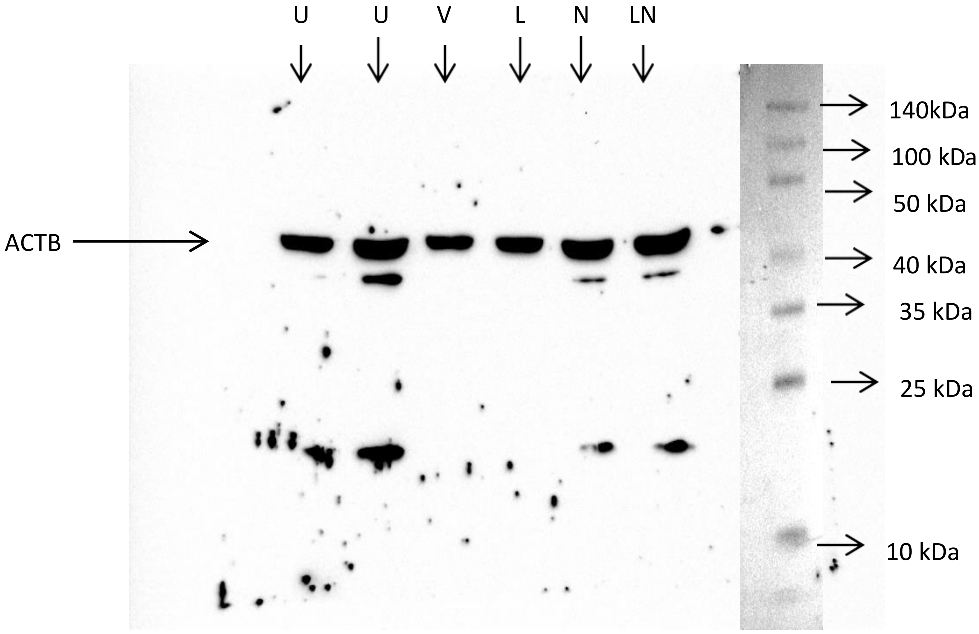

Fibroblasts: ACTB

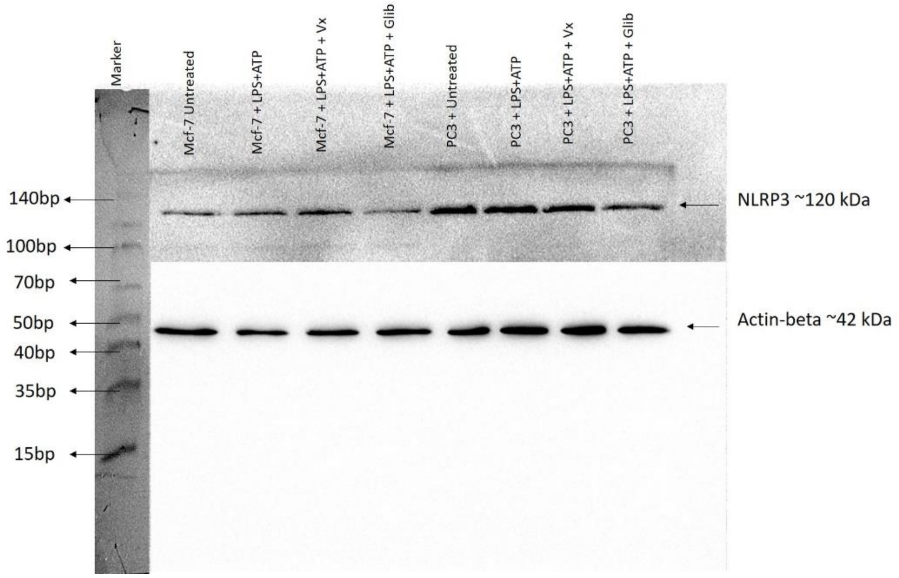

MCF7 and PC3 NLRP3 and Actb after LPS/ATP
