# Supplementary material for: Therapeutic Potential of Pharmacological Targeting NLRP3 Inflammasome Complex in Cancer
Source: Front Immunol. 2021 Feb 3;11:607881. doi: 10.3389/fimmu.2020.607881 (PMC7887322; doi:10.3389/fimmu.2020.607881)
Supplement: Supplementary file 2 [file DataSheet_2.pdf]

Supplemantary Table 1. IL-1β and IL-18 release after VX765, LPS, Nigericin and LPS&Nigericin treatments

		IL-1β										IL-18									
		inside of the cell					outside of the cell					inside of the cell					outside of the cell				
					95% CI					95% CI					95% CI					95% CI	
		MD	SE	P	L	H	MD	SE	P	L	H	MD	SE	P	L	H	MD	SE	P	L	H
A549	U-V	-0,01	1,09	1,000	-3,66	3,65	0,01	0,02	0,994	-0,07	0,10	0,54	0,09	**0,001**	0,24	0,85	0,00	1,04	1,000	-3,50	3,50
	U-VLN	-4,79	1,09	**0,009**	-8,44	-1,13	-0,87	0,02	**0,000**	-0,95	-0,78	-0,84	0,09	**0,000**	-1,14	-0,54	-0,94	1,04	0,938	-4,44	2,56
	U-L	-1,37	1,09	0,800	-5,03	2,28	-0,90	0,02	**0,000**	-0,99	-0,82	-0,39	0,09	**0,010**	-0,69	-0,08	-0,21	1,04	1,000	-3,71	3,30
	U-N	-5,58	1,09	**0,003**	-9,24	-1,93	-1,71	0,02	**0,000**	-1,80	-1,63	-1,69	0,09	**0,000**	-1,99	-1,39	-1,42	1,04	0,747	-4,92	2,08
	U-LN	-9,36	1,09	**0,000**	-13,02	-5,71	-7,67	0,02	**0,000**	-7,76	-7,59	-2,68	0,09	**0,000**	-2,98	-2,38	-2,60	1,04	0,200	-6,10	0,90
	V-L	-1,36	1,09	0,803	-5,02	2,29	-0,91	0,02	**0,000**	-1,00	-0,83	-0,93	0,09	**0,000**	-1,23	-0,63	-0,21	1,04	1,000	-3,71	3,29
	V-N	-5,57	1,09	**0,003**	-9,23	-1,92	-1,72	0,02	**0,000**	-1,81	-1,64	-2,23	0,09	**0,000**	-2,54	-1,93	-1,42	1,04	0,745	-4,92	2,08
	V-LN	-9,35	1,09	**0,000**	-13,01	-5,70	-7,69	0,02	**0,000**	-7,77	-7,60	-3,22	0,09	**0,000**	-3,52	-2,92	-2,60	1,04	0,199	-6,10	0,90
MCF7	U-V	0,03	0,04	0,971	-0,10	0,15	0,04	0,03	0,712	-0,07	0,16	0,56	0,14	**0,014**	0,10	1,02	0,06	0,78	1,000	-2,56	2,69
	U-VLN	-6,98	0,04	**0,000**	-7,11	-6,86	-0,92	0,03	**0,000**	-1,03	-0,80	-1,45	0,14	**0,000**	-1,91	-0,99	-0,65	0,78	0,955	-3,28	1,98
	U-L	-1,09	0,04	**0,000**	-1,21	-0,96	-0,92	0,03	**0,000**	-1,04	-0,80	-0,59	0,14	**0,011**	-1,05	-0,13	-0,33	0,78	0,998	-2,96	2,30
	U-N	-4,78	0,04	**0,000**	-4,92	-4,63	-2,65	0,03	**0,000**	-2,77	-2,53	-1,77	0,14	**0,000**	-2,23	-1,31	-0,68	0,78	0,948	-3,30	1,95
	U-LN	-11,89	0,04	**0,000**	-12,03	-11,75	-7,85	0,03	**0,000**	-7,97	-7,73	-2,85	0,14	**0,000**	-3,31	-2,39	-1,98	0,78	0,188	-4,61	0,64
	V-L	-1,11	0,04	**0,000**	-1,24	-0,99	-0,96	0,03	**0,000**	-1,08	-0,84	-1,15	0,14	**0,000**	-1,61	-0,69	-0,40	0,78	0,995	-3,03	2,23
	V-N	-4,80	0,04	**0,000**	-4,94	-4,66	-2,69	0,03	**0,000**	-2,81	-2,57	-2,33	0,14	**0,000**	-2,79	-1,87	-0,74	0,78	0,926	-3,37	1,89
	V-LN	-11,92	0,04	**0,000**	-12,06	-11,78	-7,89	0,03	**0,000**	-8,01	-7,78	-3,41	0,14	**0,000**	-3,88	-2,95	-2,05	0,78	0,166	-4,68	0,58
PC3	U-V	4,60	4,00	0,850	-9,31	18,51	1,80	1,01	0,511	-1,60	5,20	0,56	0,04	**0,000**	0,43	0,69	0,21	1,84	1,000	-5,96	6,38
	U-VLN	-12,93	4,00	0,072	-26,84	0,98	0,10	1,01	1,000	-3,29	3,50	-3,79	0,04	**0,000**	-3,92	-3,66	-1,29	1,84	0,978	-7,46	4,88
	U-L	-15,65	4,48	**0,048**	-31,20	-0,10	0,77	1,01	0,969	-2,63	4,17	-0,57	0,04	**0,000**	-0,70	-0,43	-0,40	1,84	1,000	-6,57	5,77
	U-N	-12,33	4,00	0,091	-26,24	1,58	1,49	1,01	0,684	-1,90	4,89	-6,54	0,04	**0,000**	-6,68	-6,41	-2,44	1,84	0,765	-8,61	3,73
	U-LN	-23,36	4,48	**0,004**	-38,92	-7,81	-3,12	1,01	0,079	-6,51	0,28	-9,17	0,04	**0,000**	-9,30	-9,04	-4,79	1,84	0,169	-10,96	1,38
	V-L	-20,26	4,48	**0,010**	-35,81	-4,70	-1,03	1,01	0,903	-4,43	2,37	-1,12	0,04	**0,000**	-1,26	-0,99	-0,61	1,84	0,999	-6,78	5,56
	V-N	-16,94	4,00	**0,016**	-30,84	-3,03	-0,31	1,01	1,000	-3,70	3,09	-7,10	0,04	**0,000**	-7,24	-6,97	-2,65	1,84	0,704	-8,82	3,52
	V-LN	-27,97	4,48	**0,001**	-43,52	-12,42	-4,92	1,01	**0,004**	-8,31	-1,52	-9,73	0,04	**0,000**	-9,86	-9,59	-4,99	1,84	0,142	-11,16	1,18
SH-SY5Y	U-V	0,42	0,05	**0,000**	0,24	0,59	0,04	0,02	0,557	-0,05	0,14	0,09	0,03	0,084	-0,01	0,18	0,02	0,08	1,000	-0,24	0,28
	U-VLN	0,05	0,05	0,901	-0,12	0,22	-0,14	0,02	0,007	-0,24	-0,05	-1,46	0,03	**0,000**	-1,56	-1,36	-0,67	0,08	**0,000**	-0,93	-0,41
	U-L	-0,03	0,05	0,990	-0,20	0,14	-0,05	0,02	0,337	-0,15	0,04	-0,09	0,03	0,087	-0,18	0,01	-0,04	0,08	0,994	-0,30	0,22
	U-N	-0,89	0,05	**0,000**	-1,06	-0,71	-1,91	0,02	**0,000**	-2,01	-1,82	-2,35	0,03	**0,000**	-2,45	-2,25	-0,83	0,08	**0,000**	-1,09	-0,57
	U-LN	-1,49	0,05	**0,000**	-1,66	-1,32	-6,82	0,02	**0,000**	-6,91	-6,72	-6,52	0,03	**0,000**	-6,62	-6,43	-1,75	0,08	**0,000**	-2,01	-1,49
	V-L	-0,45	0,05	**0,000**	-0,62	-0,27	-0,10	0,02	0,050	-0,19	0,00	-0,17	0,03	**0,001**	-0,27	-0,08	-0,06	0,08	0,965	-0,32	0,20
	V-N	-1,30	0,05	**0,000**	-1,47	-1,13	-1,96	0,02	**0,000**	-2,05	-1,86	-2,44	0,03	**0,000**	-2,53	-2,34	-0,85	0,08	**0,000**	-1,11	-0,59
	V-LN	-1,91	0,05	**0,000**	-2,08	-1,74	-6,86	0,02	**0,000**	-6,95	-6,76	-6,61	0,03	**0,000**	-6,71	-6,51	-1,77	0,08	**0,000**	-2,03	-1,51
U138MG	U-V	0,71	0,27	0,167	-0,20	1,63	5,02	0,25	**0,000**	4,12	5,91	0,54	0,10	**0,002**	0,20	0,87	0,24	1,05	1,000	-3,29	3,76
	U-VLN	-4,85	0,27	0,000	-5,77	-3,94	-2,84	0,22	**0,000**	-3,66	-2,02	-1,05	0,10	**0,000**	-1,38	-0,72	-0,79	1,05	0,970	-4,32	2,73
	U-L	-0,55	0,27	0,389	-1,47	0,37	-1,81	0,25	**0,001**	-2,71	-0,91	-0,45	0,10	**0,007**	-0,78	-0,12	-0,22	1,05	1,000	-3,74	3,31
	U-N	-8,96	0,27	**0,000**	-9,87	-8,04	-3,67	0,22	**0,000**	-4,49	-2,85	-2,25	0,10	**0,000**	-2,58	-1,92	-1,18	1,05	0,863	-4,70	2,35
	U-LN	-20,09	0,27	**0,000**	-21,00	-19,17	-5,00	0,25	**0,000**	-5,90	-4,11	-4,03	0,10	**0,000**	-4,36	-3,69	-2,40	1,05	0,270	-5,93	1,12
	V-L	-1,26	0,27	**0,006**	-2,18	-0,35	-6,83	0,25	**0,000**	-7,72	-5,93	-0,98	0,10	**0,000**	-1,32	-0,65	-0,46	1,05	0,997	-3,98	3,07
	V-N	-9,67	0,27	**0,000**	-10,59	-8,76	-8,69	0,22	**0,000**	-9,51	-7,87	-2,78	0,10	**0,000**	-3,11	-2,45	-1,42	1,05	0,754	-4,94	2,11
	V-LN	-20,80	0,27	**0,000**	-21,72	-19,88	-10,02	0,25	**0,000**	-10,91	-9,12	-4,56	0,10	**0,000**	-4,89	-4,23	-2,64	1,05	0,194	-6,16	0,89
Fibroblasts	U-V	0,41	0,49	0,953	-1,28	2,10	0,01	0,04	0,999	-0,14	0,17	0,09	0,11	0,961	-0,27	0,44	0,02	0,01	0,297	-0,01	0,04
	U-VLN	-4,77	0,44	**0,000**	-6,29	-3,26	-7,70	0,04	**0,000**	-7,86	-7,54	-2,30	0,11	0,000	-2,66	-1,95	-1,40	0,01	0,000	-1,42	-1,38
	U-L	-1,23	0,49	0,204	-2,93	0,46	-0,92	0,04	**0,000**	-1,08	-0,77	-0,03	0,11	1,000	-0,39	0,32	-0,06	0,01	**0,000**	-0,08	-0,03
	U-N	-2,35	0,44	**0,003**	-3,86	-0,83	-6,84	0,04	**0,000**	-6,99	-6,68	-1,89	0,11	**0,000**	-2,25	-1,53	-0,98	0,01	**0,000**	-1,00	-0,95
	U-LN	-7,25	0,44	**0,000**	-8,77	-5,73	-10,23	0,04	**0,000**	-10,39	-10,08	-7,02	0,11	**0,000**	-7,37	-6,66	-3,35	0,01	**0,000**	-3,37	-3,32
	V-L	-1,64	0,53	0,092	-3,50	0,21	-0,94	0,04	**0,000**	-1,09	-0,78	-0,12	0,11	0,867	-0,48	0,24	-0,07	0,01	**0,000**	-0,10	-0,05
	V-N	-2,76	0,49	**0,002**	-4,45	-1,06	-6,85	0,04	**0,000**	-7,01	-6,69	-1,97	0,11	**0,000**	-2,33	-1,62	-0,99	0,01	**0,000**	-1,02	-0,97
	V-LN	-7,66	0,49	**0,000**	-9,35	-5,97	-10,25	0,04	**0,000**	-10,40	-10,09	-7,10	0,11	**0,000**	-7,46	-6,74	-3,36	0,01	**0,000**	-3,39	-3,34

P values calculated using Oneway Anova, Post Hoc Tests, Tukey HSD.
U: Untreated; L: LPS; V: VX765; VLN: VX765 + LPS + Nigericin; N: Nigericin; LN: LPS + Nigericin
MD: Mean difference, SE: Standard Error, CI: confidence Interval, L: Lowest, H: Highest

Supplemantary Table 2: Annexin V and PI anlysis of cell death induced by VX765, LPS, Nigericin and LPS&Nigericin by Annexin V-FITC/PI assay

Cell line		A549					MCF7					PC3				
					95% CI					95% CI					95% CI	
Comparison		MD	SE	P	L	H	MD	SE	P	L	H	MD	SE	P	L	H
U	V	0,44	0,17	0,1560	-0,10	0,98	0,20	0,11	0,5199	-0,16	0,56	0,18	0,29	0,9891	-0,73	1,09
	L	-0,30	0,17	0,5310	-0,84	0,24	0,24	0,11	0,3259	-0,12	0,60	0,98	0,29	**0,0296**	0,07	1,89
	N	-1,92	0,17	**0,0000**	-2,46	-1,38	-0,14	0,11	0,8236	-0,50	0,22	-7,46	0,29	**0,0000**	-8,37	-6,55
	LN	-2,26	0,17	**0,0000**	-2,80	-1,72	-3,04	0,11	**0,0000**	-3,40	-2,68	-18,34	0,29	**0,0000**	-19,25	-17,43
L	U	0,30	0,17	0,5310	-0,24	0,84	-0,24	0,11	0,3259	-0,60	0,12	-0,98	0,29	**0,0296**	-1,89	-0,07
	V	0,74	0,17	**0,0034**	0,20	1,28	-0,04	0,11	0,9992	-0,40	0,32	-0,80	0,29	0,1081	-1,71	0,11
	N	0,68	0,17	**0,0078**	0,14	1,22	-0,38	0,11	**0,0311**	-0,74	-0,02	-8,44	0,29	**0,0000**	-9,35	-7,53
	LN	-1,96	0,17	**0,0000**	-2,50	-1,42	-3,28	0,11	**0,0000**	-3,64	-2,92	-19,32	0,29	**0,0000**	-20,23	-18,41
V	U	-0,44	0,17	0,1560	-0,98	0,10	-0,20	0,11	0,5199	-0,56	0,16	-0,18	0,29	0,9891	-1,09	0,73
	L	-0,74	0,17	**0,0034**	-1,28	-0,20	0,04	0,11	0,9992	-0,32	0,40	0,80	0,29	0,1081	-0,11	1,71
	N	-0,06	0,17	0,9993	-0,60	0,48	-0,34	0,11	0,0663	-0,70	0,02	-7,64	0,29	**0,0000**	-8,55	-6,73
	LN	-2,70	0,17	**0,0000**	-3,24	-2,16	-3,24	0,11	**0,0000**	-3,60	-2,88	-18,52	0,29	**0,0000**	-19,43	-17,61
N	U	1,92	0,17	**0,0000**	1,38	2,46	0,14	0,11	0,8236	-0,22	0,50	7,46	0,29	**0,0000**	6,55	8,37
	L	1,62	0,17	**0,0000**	1,08	2,16	0,38	0,11	**0,0311**	0,02	0,74	8,44	0,29	**0,0000**	7,53	9,35
	V	2,36	0,17	**0,0000**	1,82	2,90	0,34	0,11	0,0663	-0,02	0,70	7,64	0,29	**0,0000**	6,73	8,55
	LN	-0,34	0,17	0,3970	-0,88	0,20	-2,90	0,11	**0,0000**	-3,26	-2,54	-10,88	0,29	**0,0000**	-11,79	-9,97
LN	U	2,26	0,17	**0,0000**	1,72	2,80	3,04	0,11	**0,0000**	2,68	3,40	18,34	0,29	**0,0000**	17,43	19,25
	L	1,96	0,17	**0,0000**	1,42	2,50	3,28	0,11	**0,0000**	2,92	3,64	19,32	0,29	**0,0000**	18,41	20,23
	V	2,70	0,17	**0,0000**	2,16	3,24	3,24	0,11	**0,0000**	2,88	3,60	18,52	0,29	**0,0000**	17,61	19,43
	N	2,64	0,17	**0,0000**	2,10	3,18	2,90	0,11	**0,0000**	2,54	3,26	10,88	0,29	**0,0000**	9,97	11,79

Cell line		SH-SY5Y					U138MG					Fibroblasts				
					95% CI					95% CI					95% CI	
Comparison		MD	SE	P	L	H	MD	SE	P	L	H	MD	SE	P	L	H
U	V	0,10	0,22	0,9972	-0,58	0,78	0,04	0,34	1,0000	-1,03	1,11	0,24	0,08	0,0691	-0,01	0,49
	L	0,94	0,22	**0,0031**	0,26	1,62	0,06	0,34	1,0000	-1,01	1,13	-0,30	0,08	**0,0134**	-0,55	-0,05
	N	-1,34	0,22	**0,0000**	-2,02	-0,66	-9,38	0,34	**0,0000**	-10,45	-8,31	-0,06	0,08	0,9755	-0,31	0,19
	LN	-4,64	0,22	**0,0000**	-5,32	-3,96	-10,88	0,34	**0,0000**	-11,95	-9,81	-0,66	0,08	**0,0000**	-0,91	-0,41
L	U	-0,94	0,22	**0,0031**	-1,62	-0,26	-0,06	0,34	1,0000	-1,13	1,01	0,30	0,08	**0,0134**	0,05	0,55
	V	-0,84	0,22	**0,0094**	-1,52	-0,16	-0,02	0,34	1,0000	-1,09	1,05	0,54	0,08	**0,0000**	0,29	0,79
	N	-2,28	0,22	**0,0000**	-2,96	-1,60	-9,44	0,34	**0,0000**	-10,51	-8,37	0,24	0,08	0,0691	-0,01	0,49
	LN	-5,58	0,22	**0,0000**	-6,26	-4,90	-10,94	0,34	**0,0000**	-12,01	-9,87	-0,36	0,08	**0,0023**	-0,61	-0,11
V	U	-0,10	0,22	0,9972	-0,78	0,58	-0,04	0,34	1,0000	-1,11	1,03	-0,24	0,08	0,0691	-0,49	0,01
	L	0,84	0,22	**0,0094**	0,16	1,52	0,02	0,34	1,0000	-1,05	1,09	-0,54	0,08	**0,0000**	-0,79	-0,29
	N	-1,44	0,22	**0,0000**	-2,12	-0,76	-9,42	0,34	**0,0000**	-10,49	-8,35	-0,30	0,08	**0,0134**	-0,55	-0,05
	LN	-4,74	0,22	**0,0000**	-5,42	-4,06	-10,92	0,34	**0,0000**	-11,99	-9,85	-0,90	0,08	**0,0000**	-1,15	-0,65
N	U	1,34	0,22	**0,0000**	0,66	2,02	9,38	0,34	**0,0000**	8,31	10,45	0,06	0,08	0,9755	-0,19	0,31
	L	2,28	0,22	**0,0000**	1,60	2,96	9,44	0,34	**0,0000**	8,37	10,51	-0,24	0,08	0,0691	-0,49	0,01
	V	1,44	0,22	**0,0000**	0,76	2,12	9,42	0,34	**0,0000**	8,35	10,49	0,30	0,08	**0,0134**	0,05	0,55
	LN	-3,30	0,22	**0,0000**	-3,98	-2,62	-1,50	0,34	**0,0026**	-2,57	-0,43	-0,60	0,08	**0,0000**	-0,85	-0,35
LN	U	4,64	0,22	**0,0000**	3,96	5,32	10,88	0,34	**0,0000**	9,81	11,95	0,66	0,08	**0,0000**	0,41	0,91
	L	5,58	0,22	**0,0000**	4,90	6,26	10,94	0,34	**0,0000**	9,87	12,01	0,36	0,08	**0,0023**	0,11	0,61
	V	4,74	0,22	**0,0000**	4,06	5,42	10,92	0,34	**0,0000**	9,85	11,99	0,90	0,08	**0,0000**	0,65	1,15
	N	3,30	0,22	**0,0000**	2,62	3,98	1,50	0,34	**0,0026**	0,43	2,57	0,60	0,08	**0,0000**	0,35	0,85

P values calculated using Oneway Anova, Post Hoc Tests, Tukey HSD.
U: Untreated; L: LPS; V: VX765; VLN: VX765 + LPS + Nigericin; N: Nigericin; LN: LPS + Nigericin
MD: Mean difference, SE: Standard Error, CI: confidence Interval, L: Lowest, H: Highest

Supplemantary Table 3. LDH release after VX765, LPS, Nigericin and LPS&Nigericin treatments

	Type	N	Mean	SD	SE	CI 95 %	**Kruskal-Wallis multiple comparison**	Z	P.unadj
**A549**	U	3	0,69	0,77	0,44	1,91	U - VX	-1,38	0,1663
	VX	3	2,52	0,52	0,30	1,30	L - U	1,11	0,2681
	L	3	2,52	0,00	0,00	0,00	N - U	3,32	**0,0009**
	N	3	13,22	0,00	0,00	0,00	LN - U	2,49	**0,0127**
	LN	3	7,72	0,00	0,00	0,00	L - VX	-0,28	0,7819
							N - VX	1,94	0,0526
							LN - VX	1,11	0,2681
**MCF7**	U	2	0,23	0,01	0,01	0,11	U - VX	1,00	0,3188
	VX	2	0,14	0,00	0,00	0,01	L - U	0,00	1,0000
	L	2	0,21	0,06	0,04	0,52	N - U	1,66	0,0966
	N	2	25,08	0,00	0,00	0,00	LN - U	1,00	0,3188
	LN	2	16,58	0,00	0,00	0,00	L - VX	1,00	0,3188
							N - VX	2,66	0,0078
							LN - VX	1,99	0,0462
**PC3**	U	3	8,23	3,96	2,29	9,84	U - VX	-0,55	0,5839
	VX	3	14,93	5,18	2,99	12,87	L - U	2,19	0,0285
	L	3	28,73	4,88	2,82	12,13	N - U	3,10	**0,0019**
	N	3	76,60	5,67	3,27	14,08	LN - U	1,46	0,1441
	LN	3	22,44	3,19	1,84	7,92	L - VX	1,64	0,1003
							N - VX	2,56	0,0106
							LN - VX	0,91	0,3613
**SH-SY5Y**	U	3	0,50	0,10	0,06	0,25	U - VX	1,37	0,1694
	VX	3	0,18	0,02	0,01	0,04	L - U	0,09	0,9270
	L	3	0,50	0,18	0,11	0,46	N - U	1,65	0,0991
	N	3	4,00	0,00	0,00	0,00	LN - U	-0,37	0,7140
	LN	3	0,43	0,30	0,18	0,75	L - VX	1,47	0,1427
							N - VX	3,02	0,0025
							LN - VX	1,01	0,3136
**U138MG**	U	3	3,99	1,44	0,83	3,58	U - VX	0,37	0,7150
	VX	3	3,63	0,57	0,33	1,41	L - U	0,64	0,5228
	L	3	4,73	0,05	0,03	0,13	N - U	2,56	**0,0106**
	N	3	40,63	1,21	0,70	2,99	LN - U	1,73	0,0828
	LN	3	30,34	3,14	1,81	7,81	L - VX	1,00	0,3153
							N - VX	2,92	0,0035
							LN - VX	2,10	0,0358
**Fibroblasts**	U	2	0,22	0,01	0,01	0,07	U - VX	-1,00	0,3173
	VX	2	0,49	0,00	0,00	0,00	L - U	1,00	0,3173
	L	2	0,63	0,24	0,17	2,13	N - U	2,67	**0,0077**
	N	2	4,09	0,00	0,00	0,00	LN - U	2,00	**0,0455**
	LN	2	3,13	0,00	0,00	0,00	L - VX	0,00	1,0000
							N - VX	1,67	0,0956
							LN - VX	1,00	0,3173

P values calculated using Kruskal-Wallis multiple comparison test.

U: Untreated; L: LPS; V: VX765; VLN: VX765 + LPS + Nigericin; N: Nigericin; LN: LPS + Nigericin

MD: Mean difference, SE: Standard Error, CI: confidence Interval, L: Lowest, H: Highest

Supplemantary Table 4A: Cytokine secretion levels in A549 cells after VX765, LPS, Nigericin and LPS&Nigericin treatments

	Type	N	Mean	SD	SE	CI 95 %	**Kruskal-Wallis multiple comparison**	Z	P
**6Cine/CCL21 (12)**	U	2	52,39	3,34	2,37	30,05	U - V	0,48	0,632
	V	2	43,00	0,00	0,00	0,00	U - L	-0,96	0,338
	L	2	75,64	1,05	0,75	9,47	U - N	-0,48	0,632
	N	2	65,17	2,03	1,44	18,23	U - LN	1,44	0,151
	LN	2	31,82	0,30	0,22	2,73	L - V	1,44	0,151
	C	2	16,71	0,52	0,37	4,64	N - V	0,96	0,338
							LN - V	-0,96	0,338
							C - U	-1,91	0,056
							C - N	-2,39	**0,017**
							C - LN	-0,48	0,632
**BCA-1/CXCL13 (74)**	U	2	1,28	0,45	0,32	4,00	U - V	-1,31	0,189
	V	2	2,94	0,08	0,06	0,70	U - L	-1,31	0,189
	L	2	3,12	0,36	0,26	3,24	U - N	-1,31	0,189
	N	2	2,96	0,01	0,01	0,13	U - LN	0,48	0,633
	LN	2	0,94	0,30	0,21	2,67	L - V	0,00	1,000
	C	2	0,58	0,01	0,01	0,13	N - V	0,00	1,000
							LN - V	-1,79	0,073
							C - U	-1,08	0,282
							C - N	-2,39	**0,017**
							C - LN	-0,60	0,550
**CTACK/CCL27 (72)**	U	2	3,33	0,93	0,66	8,39	U - V	-0,96	0,339
	V	2	5,40	0,21	0,15	1,84	U - L	0,00	1,000
	L	2	3,24	0,45	0,32	4,07	U - N	-1,43	0,151
	N	2	5,68	0,06	0,05	0,57	U - LN	1,43	0,151
	LN	2	1,37	0,04	0,03	0,32	L - V	-0,96	0,339
	C	2	1,56	0,04	0,03	0,32	N - V	0,48	0,633
							LN - V	-2,39	**0,017**
							C - U	-0,96	0,339
							C - N	-2,39	**0,017**
							C - LN	0,48	0,633
**ENA-78/CXCL5 (73)**	U	2	25181,28	1427,01	1009,05	12821,13	U - V	0,00	1,000
	V	2	25468,61	693,30	490,24	6229,03	U - L	-0,60	0,550
	L	2	27606,95	2907,61	2055,99	26123,83	U - N	-0,84	0,403
	N	2	26419,86	173,08	122,39	1555,05	U - LN	1,31	0,189
	LN	2	3587,64	815,09	576,36	7323,28	L - V	0,60	0,550
	C	2	1071,07	39,84	28,17	357,93	N - V	0,84	0,403
							LN - V	-1,31	0,189
							C - U	-1,79	0,073
							C - N	-2,63	**0,009**
							C - LN	-0,48	0,633
**Eotaxin/CCL11 (43)**	U	2	26,91	1,10	0,78	9,91	U - V	-0,48	0,633
	V	2	29,60	0,06	0,05	0,57	U - L	-0,96	0,339
	L	2	33,37	1,91	1,35	17,15	U - N	0,48	0,633
	N	2	20,91	1,34	0,95	12,01	U - LN	1,91	0,056
	LN	2	10,82	0,17	0,12	1,52	L - V	0,48	0,633
	C	2	12,63	0,33	0,24	2,99	N - V	-0,96	0,339
							LN - V	-2,39	**0,017**
							C - U	-1,43	0,151
							C - N	-0,96	0,339
							C - LN	0,48	0,633
**Eotaxin2/CCL24 (30)**	U	2	4,00	0,09	0,07	0,83	U - V	-0,96	0,338
	V	2	5,15	0,35	0,25	3,18	U - L	-0,24	0,811
	L	2	4,21	0,37	0,26	3,30	U - N	0,24	0,811
	N	2	3,94	0,06	0,05	0,57	U - LN	1,62	0,106
	LN	2	2,82	0,27	0,19	2,41	L - V	-0,72	0,473
	C	2	2,70	0,10	0,07	0,89	N - V	-1,20	0,231
							LN - V	-2,57	**0,010**
							C - U	-1,73	0,083
							C - N	-1,50	0,135
							C - LN	-0,12	0,905
**Eotaxin-3/CCL26 (65)**	U	2	7,51	0,23	0,17	2,10	U - V	-0,96	0,339
	V	2	11,89	0,15	0,11	1,33	U - L	-0,48	0,633
	L	2	10,47	0,55	0,39	4,96	U - N	0,48	0,633
	N	2	6,90	0,16	0,11	1,40	U - LN	1,43	0,151
	LN	2	3,82	0,79	0,56	7,12	L - V	-0,48	0,633
	C	2	2,42	0,14	0,10	1,27	N - V	-1,43	0,151
							LN - V	-2,39	**0,017**
							C - U	-1,91	0,056
							C - N	-1,43	0,151
							C - LN	-0,48	0,633
**Fractalkine?CX3CL1 (77)**	U	2	414,60	0,03	0,02	0,25	U - V	-1,43	0,151
	V	2	868,12	17,62	12,46	158,32	U - L	-0,96	0,339
	L	2	641,09	13,80	9,76	123,95	U - N	-0,48	0,633
	N	2	505,57	12,22	8,64	109,78	U - LN	0,48	0,633
	LN	2	337,33	43,47	30,74	390,59	L - V	-0,48	0,633

	C	2	13,44	0,53	0,38	4,76	N - V	-0,96	0,339
							LN - V	-1,91	0,056
							C - U	-1,43	0,151
							C - N	-1,91	0,056
							C - LN	-0,96	0,339
**GCP/CXCL6 (15)**	U	2	16,25	0,25	0,18	2,29	U - V	-0,48	0,633
	V	2	21,43	0,76	0,54	6,80	U - L	-1,43	0,151
	L	2	40,43	2,26	1,60	20,33	U - N	-0,96	0,339
	N	2	29,04	0,11	0,08	1,02	U - LN	0,96	0,339
	LN	2	8,45	0,75	0,53	6,73	L - V	0,96	0,339
	C	2	2,80	0,71	0,50	6,35	N - V	0,48	0,633
							LN - V	-1,43	0,151
							C - U	-1,43	0,151
							C - N	-2,39	**0,017**
							C - LN	-0,48	0,633
**GMCSF (34)**	U	2	31,71	3,39	2,40	30,49	U - V	-1,20	0,231
	V	2	37,44	2,36	1,67	21,22	U - L	-2,27	**0,023**
	L	2	65,83	6,05	4,28	54,38	U - N	-2,03	**0,042**
	N	2	62,32	5,56	3,94	50,00	U - LN	-0,48	0,632
	LN	2	35,01	0,21	0,15	1,91	L - V	1,08	0,282
	C	2	13,22	0,73	0,52	6,54	N - V	0,84	0,402
							LN - V	-0,72	0,473
							C - U	-0,48	0,632
							C - N	-2,51	**0,012**
							C - LN	-0,96	0,338
**Gro-a/CXCL1 (61)**	U	2	34471,52	358,81	253,72	3223,82	U - V	2,03	**0,042**
	V	2	2865,06	208,12	147,17	1869,91	U - L	1,20	0,232
	L	2	3349,25	47,98	33,93	431,12	U - N	2,87	**0,004**
	N	2	1674,71	361,12	255,35	3244,53	U - LN	2,27	**0,023**
	LN	2	2588,59	526,50	372,29	4730,39	L - V	0,84	0,403
	C	2	6896,89	568,71	402,14	5109,67	N - V	-0,84	0,403
							LN - V	-0,24	0,811
							C - U	-0,48	0,633
							C - N	2,39	**0,017**
							C - LN	1,79	0,073
**Gro-b/CXCL2 (78)**	U	2	815,26	61,40	43,42	551,64	U - V	-0,36	0,720
	V	2	889,00	78,91	55,80	709,01	U - L	0,60	0,550
	L	2	714,57	4,14	2,93	37,17	U - N	-1,31	0,189
	N	2	1530,56	358,13	253,24	3217,66	U - LN	1,08	0,282
	LN	2	329,03	60,66	42,89	544,97	L - V	-0,96	0,339
	C	2	31,84	0,71	0,50	6,35	N - V	0,96	0,339
							LN - V	-1,43	0,151
							C - U	-1,55	0,120
							C - N	-2,87	**0,004**
							C - LN	-0,48	0,633
**I-309/CCL1 (20)**	U	2	21,95	4,31	3,05	38,75	U - V	-0,24	0,811
	V	2	23,44	0,04	0,03	0,32	U - L	0,24	0,811
	L	2	21,76	1,17	0,83	10,55	U - N	0,96	0,339
	N	2	17,92	0,05	0,04	0,44	U - LN	1,91	0,056
	LN	2	10,19	0,93	0,66	8,39	L - V	-0,48	0,633
	C	2	8,34	0,15	0,11	1,33	N - V	-1,20	0,232
							LN - V	-2,15	**0,031**
							C - U	-2,39	**0,017**
							C - N	-1,43	0,151
							C - LN	-0,48	0,633
**IFNg (21)**	U	2	1,67	0,66	0,47	5,91	U - V	-0,12	0,905
	V	2	2,08	0,05	0,04	0,44	U - L	-1,43	0,151
	L	2	4,90	0,25	0,18	2,22	U - N	-0,96	0,339
	N	2	3,80	0,28	0,20	2,48	U - LN	0,12	0,905
	LN	2	1,80	0,39	0,28	3,49	L - V	1,31	0,189
	C	2	0,28	0,01	0,01	0,13	N - V	0,84	0,403
							LN - V	-0,24	0,811
							C - U	-1,43	0,151
							C - N	-2,39	**0,017**
							C - LN	-1,31	0,189
**IL1b (39)**	U	2	37,52	0,10	0,07	0,89	U - V	0,00	1,000
	V	2	36,49	3,80	2,69	34,12	U - L	-2,15	**0,031**
	L	2	348,46	11,55	8,17	103,75	U - N	-1,67	0,094
	N	2	276,91	2,92	2,07	26,24	U - LN	-1,20	0,232
	LN	2	132,94	5,48	3,88	49,24	L - V	2,15	**0,031**
	C	2	4,72	0,02	0,02	0,19	N - V	1,67	0,094
							LN - V	1,20	0,232
							C - U	-0,72	0,473
							C - N	-2,39	**0,017**
							C - LN	-1,91	0,056
**IL2 (38)**	U	2	2,92	0,89	0,63	8,00	U - V	-0,96	0,339
	V	2	5,66	0,27	0,19	2,41	U - L	-1,79	0,073
	L	2	10,10	1,32	0,94	11,88	U - N	-1,20	0,232
	N	2	6,45	0,86	0,61	7,75	U - LN	0,36	0,720
	LN	2	2,23	0,41	0,29	3,68	L - V	0,84	0,403
	C	2	1,18	0,13	0,09	1,14	N - V	0,24	0,811

							LN - V	-1,31	0,189
							C - U	-1,08	0,282
							C - N	-2,27	**0,023**
							C - LN	-0,72	0,473
**IL4 (52)**	U	2	5,32	1,22	0,86	10,93	U - V	0,24	0,811
	V	2	5,79	0,23	0,16	2,03	U - L	-0,96	0,339
	L	2	7,22	0,62	0,44	5,59	U - N	-0,24	0,811
	N	2	6,08	0,07	0,05	0,64	U - LN	1,08	0,282
	LN	2	3,74	0,06	0,04	0,51	L - V	1,20	0,232
	C	2	1,53	0,04	0,03	0,38	N - V	0,48	0,633
							LN - V	-0,84	0,403
							C - U	-1,91	0,056
							C - N	-2,15	**0,031**
							C - LN	-0,84	0,403
**IL6 (19)**	U	2	10916,73	1072,14	758,12	9632,83	U - V	0,48	0,633
	V	2	5130,88	382,52	270,48	3436,77	U - L	0,96	0,339
	L	2	4235,36	440,17	311,25	3954,74	U - N	1,43	0,151
	N	2	2216,69	183,85	130,00	1651,81	U - LN	2,87	**0,004**
	LN	2	928,73	84,97	60,08	763,39	L - V	-0,48	0,633
	C	2	1346,88	38,11	26,95	342,43	N - V	-0,96	0,339
							LN - V	-2,39	**0,017**
							C - U	-1,91	0,056
							C - N	-0,48	0,633
							C - LN	0,96	0,339
**IL8/CXCL8 (54)**	U	2	14714,82	120,10	84,92	1079,01	U - V	1,31	0,189
	V	2	10294,05	513,84	363,34	4616,67	U - L	1,08	0,282
	L	2	11029,34	600,31	424,48	5393,53	U - N	2,27	**0,023**
	N	2	8466,66	375,23	265,33	3371,34	U - LN	2,87	**0,004**
	LN	2	6226,80	1325,71	937,42	11910,99	L - V	0,24	0,811
	C	2	12553,65	109,75	77,61	986,07	N - V	-0,96	0,339
							LN - V	-1,55	0,120
							C - U	-0,48	0,633
							C - N	1,79	0,073
							C - LN	2,39	**0,017**
**IL10 (56)**	U	2	28,98	0,86	0,61	7,75	U - V	-0,72	0,473
	V	2	31,19	0,25	0,18	2,29	U - L	-1,20	0,232
	L	2	35,90	0,49	0,35	4,45	U - N	0,00	1,000
	N	2	28,63	0,04	0,03	0,38	U - LN	1,08	0,282
	LN	2	22,34	2,00	1,42	17,98	L - V	0,48	0,633
	C	2	21,23	1,48	1,05	13,34	N - V	-0,72	0,473
							LN - V	-1,79	0,073
							C - U	-1,55	0,120
							C - N	-1,55	0,120
							C - LN	-0,48	0,633
**IL16 (27)**	U	2	77,95	2,31	1,64	20,77	U - V	0,72	0,473
	V	2	63,10	1,77	1,25	15,88	U - L	-0,72	0,473
	L	2	118,88	1,66	1,18	14,93	U - N	0,00	1,000
	N	2	77,53	7,23	5,12	64,99	U - LN	1,20	0,231
	LN	2	32,46	0,89	0,63	8,00	L - V	1,44	0,151
	C	2	14,67	0,67	0,48	6,04	N - V	0,72	0,473
							LN - V	-0,48	0,632
							C - U	-2,15	**0,031**
							C - N	-2,15	**0,031**
							C - LN	-0,96	0,338
**IP10/CXCL10 (48)**	U	2	2,84	0,35	0,25	3,18	U - V	-0,72	0,473
	V	2	4,05	0,01	0,01	0,06	U - L	0,00	1,000
	L	2	2,95	0,08	0,06	0,76	U - N	1,08	0,282
	N	2	2,52	0,04	0,03	0,32	U - LN	1,91	0,056
	LN	2	1,56	0,16	0,11	1,40	L - V	-0,72	0,473
	C	2	1,53	0,03	0,02	0,25	N - V	-1,79	0,073
							LN - V	-2,63	**0,009**
							C - U	-1,91	0,056
							C - N	-0,84	0,403
							C - LN	0,00	1,000
**I-TAC/CXCL11 (25)**	U	2	7,53	1,22	0,87	10,99	U - V	-0,12	0,905
	V	2	7,72	0,23	0,17	2,10	U - L	0,12	0,905
	L	2	7,50	0,40	0,29	3,62	U - N	0,96	0,339
	N	2	6,36	0,28	0,20	2,54	U - LN	1,91	0,056
	LN	2	3,88	0,05	0,04	0,44	L - V	-0,24	0,811
	C	2	3,15	0,01	0,01	0,13	N - V	-1,08	0,282
							LN - V	-2,03	**0,042**
							C - U	-2,39	**0,017**
							C - N	-1,43	0,151
							C - LN	-0,48	0,633
**MCP-1/CCL2 (53)**	U	2	233,04	3,71	2,62	33,29	U - V	0,48	0,633
	V	2	197,07	6,08	4,30	54,64	U - L	-0,48	0,633
	L	2	243,39	10,59	7,49	95,17	U - N	0,96	0,339
	N	2	175,68	10,04	7,10	90,21	U - LN	1,91	0,056
	LN	2	61,67	3,31	2,34	29,73	L - V	0,96	0,339
	C	2	6,45	0,24	0,17	2,16	N - V	-0,48	0,633
							LN - V	-1,43	0,151

							C - U	-2,39	**0,017**
							C - N	-1,43	0,151
							C - LN	-0,48	0,633
**MCP-2/CCL8 (57)**	U	2	0,72	0,18	0,13	1,59	U - V	-1,32	0,188
	V	2	1,09	0,01	0,01	0,06	U - L	-0,72	0,472
	L	2	0,91	0,15	0,11	1,33	U - N	-0,12	0,905
	N	2	0,73	0,00	0,00	0,00	U - LN	1,08	0,281
	LN	2	0,45	0,00	0,00	0,00	L - V	-0,60	0,549
	C	2	0,38	0,03	0,02	0,25	N - V	-1,20	0,231
							LN - V	-2,40	**0,017**
							C - U	-1,56	0,119
							C - N	-1,68	0,094
							C - LN	-0,48	0,632
**MCP-3/CCL7 (26)**	U	2	28,46	3,24	2,29	29,10	U - V	-0,48	0,632
	V	2	48,67	0,74	0,53	6,67	U - L	-1,91	0,056
	L	2	192,18	0,54	0,38	4,83	U - N	-1,44	0,151
	N	2	170,03	3,89	2,75	34,94	U - LN	-0,96	0,338
	LN	2	125,64	2,67	1,89	23,95	L - V	1,44	0,151
	C	2	7,57	0,00	0,00	0,00	N - V	0,96	0,338
							LN - V	0,48	0,632
							C - U	-0,96	0,338
							C - N	-2,39	**0,017**
							C - LN	-1,91	0,056
**MCP-4/CCL13 (28)**	U	2	84,65	6,81	4,82	61,18	U - V	0,48	0,633
	V	2	77,17	0,66	0,47	5,97	U - L	1,91	0,056
	L	2	44,07	0,16	0,12	1,46	U - N	2,51	**0,012**
	N	2	31,75	2,59	1,83	23,25	U - LN	2,75	**0,006**
	LN	2	26,03	8,95	6,33	80,43	L - V	-1,43	0,151
	C	2	63,10	8,20	5,80	73,70	N - V	-2,03	**0,042**
							LN - V	-2,27	**0,023**
							C - U	-1,08	0,282
							C - N	1,43	0,151
							C - LN	1,67	0,094
**MDC/CCL22 (29)**	U	2	5,19	0,75	0,53	6,73	U - V	-0,90	0,369
	V	2	6,59	0,07	0,05	0,64	U - L	-0,18	0,857
	L	2	5,43	0,29	0,21	2,60	U - N	1,02	0,309
	N	2	4,35	0,20	0,14	1,78	U - LN	1,98	**0,048**
	LN	2	2,80	0,07	0,05	0,64	L - V	-0,72	0,472
	C	2	3,51	0,00	0,00	0,00	N - V	-1,92	0,055
							LN - V	-2,87	**0,004**
							C - U	-1,50	0,134
							C - N	-0,48	0,632
							C - LN	0,48	0,632
**MIF (35)**	U	2	288,45	9,19	6,50	82,53	U - V	0,96	0,339
	V	2	200,99	1,10	0,78	9,91	U - L	0,48	0,633
	L	2	234,04	6,51	4,61	58,51	U - N	-0,96	0,339
	N	2	391,76	15,20	10,75	136,53	U - LN	-1,43	0,151
	LN	2	784,34	13,67	9,67	122,81	L - V	0,48	0,633
	C	2	315,87	3,92	2,77	35,20	N - V	1,91	0,056
							LN - V	2,39	**0,017**
							C - U	0,48	0,633
							C - N	-0,48	0,633
							C - LN	-0,96	0,339
**MIG/CXCL9 (14)**	U	2	24,34	5,96	4,22	53,56	U - V	-0,48	0,633
	V	2	28,45	0,76	0,54	6,80	U - L	-1,08	0,282
	L	2	33,84	2,11	1,49	18,93	U - N	0,12	0,905
	N	2	26,87	0,97	0,69	8,70	U - LN	1,31	0,189
	LN	2	15,82	0,33	0,23	2,92	L - V	0,60	0,550
	C	2	8,61	0,13	0,09	1,14	N - V	-0,60	0,550
							LN - V	-1,79	0,073
							C - U	-1,79	0,073
							C - N	-1,67	0,094
							C - LN	-0,48	0,633
**MIP-1a/CCL3 (55)**	U	2	1,70	0,35	0,25	3,11	U - V	-0,72	0,473
	V	2	2,54	0,04	0,03	0,38	U - L	-1,68	0,094
	L	2	3,82	0,21	0,15	1,91	U - N	-1,20	0,231
	N	2	2,67	0,08	0,06	0,76	U - LN	0,72	0,473
	LN	2	1,21	0,13	0,10	1,21	L - V	0,96	0,338
	C	2	1,07	0,03	0,02	0,25	N - V	0,48	0,632
							LN - V	-1,44	0,151
							C - U	-1,20	0,231
							C - N	-2,39	**0,017**
							C - LN	-0,48	0,632
**MIP1b/CCL15 (66)**	U	2	7,28	1,72	1,22	15,44	U - V	-1,20	0,232
	V	2	10,24	0,05	0,04	0,44	U - L	-1,79	0,073
	L	2	11,52	1,39	0,98	12,45	U - N	-0,96	0,339
	N	2	9,44	1,09	0,77	9,78	U - LN	0,00	1,000
	LN	2	6,44	0,28	0,20	2,48	L - V	0,60	0,550
	C	2	4,68	0,30	0,22	2,73	N - V	-0,24	0,811
							LN - V	-1,20	0,232
							C - U	-1,08	0,282

							C - N	-2,03	**0,042**
							C - LN	-1,08	0,282
**MIP-3a/CCL20 (62)**	U	2	172,71	17,92	12,67	160,99	U - V	-1,02	0,309
	V	2	1062,49	1101,11	778,60	9893,05	U - L	-0,90	0,370
	L	2	297,22	18,85	13,33	169,37	U - N	0,72	0,473
	N	2	154,41	5,14	3,64	46,19	U - LN	1,20	0,231
	LN	2	38,81	20,66	14,61	185,64	L - V	-0,12	0,905
	C	2	2,22	0,08	0,06	0,76	N - V	-1,73	0,083
							LN - V	-2,21	**0,027**
							C - U	-1,68	0,094
							C - N	-0,96	0,338
							C - LN	-0,48	0,632
**MIP3-b/CCL19 (76)**	U	2	14,36	2,71	1,92	24,33	U - V	0,00	1,000
	V	2	14,33	0,07	0,05	0,64	U - L	-1,68	0,094
	L	2	47,13	6,84	4,84	61,43	U - N	-1,20	0,231
	N	2	38,14	4,99	3,53	44,85	U - LN	-0,72	0,473
	LN	2	29,91	4,00	2,83	35,96	L - V	1,68	0,094
	C	2	6,42	0,00	0,00	0,00	N - V	1,20	0,231
							LN - V	0,72	0,473
							C - U	-1,20	0,231
							C - N	-2,39	**0,017**
							C - LN	-1,91	0,056
**MPIF-1/CCL23 (37)**	U	2	11,55	0,57	0,40	5,08	U - V	0,00	1,000
	V	2	11,50	0,25	0,18	2,29	U - L	-1,20	0,232
	L	2	16,47	0,49	0,35	4,38	U - N	-0,72	0,473
	N	2	13,09	0,49	0,35	4,45	U - LN	0,72	0,473
	LN	2	8,22	0,17	0,12	1,52	L - V	1,20	0,232
	C	2	2,87	0,13	0,09	1,14	N - V	0,72	0,473
							LN - V	-0,72	0,473
							C - U	-1,67	0,094
							C - N	-2,39	**0,017**
							C - LN	-0,96	0,339
**SCYB16/CXCL16 (64)**	U	2	54,43	2,85	2,02	25,60	U - V	-0,48	0,633
	V	2	60,20	0,09	0,07	0,83	U - L	-1,43	0,151
	L	2	98,41	1,94	1,37	17,41	U - N	-0,96	0,339
	N	2	61,53	0,06	0,04	0,51	U - LN	1,20	0,232
	LN	2	9,30	0,18	0,13	1,59	L - V	0,96	0,339
	C	2	17,26	0,33	0,24	2,99	N - V	0,48	0,633
							LN - V	-1,67	0,094
							C - U	-0,48	0,633
							C - N	-1,43	0,151
							C - LN	0,72	0,473
**SDF-1ab/CXCL12 (22)**	U	2	34,00	4,77	3,38	42,88	U - V	-1,67	0,094
	V	2	48,33	0,83	0,59	7,50	U - L	-1,08	0,282
	L	2	43,96	4,50	3,18	40,41	U - N	-0,84	0,403
	N	2	42,15	3,13	2,22	28,14	U - LN	0,72	0,473
	LN	2	28,66	0,47	0,34	4,26	L - V	-0,60	0,550
	C	2	19,89	0,45	0,32	4,00	N - V	-0,84	0,403
							LN - V	-2,39	**0,017**
							C - U	-1,20	0,232
							C - N	-2,03	**0,042**
							C - LN	-0,48	0,633
**TARC/CCL17 (67)**	U	2	14,49	3,02	2,14	27,13	U - V	0,00	1,000
	V	2	16,15	0,25	0,18	2,29	U - L	-1,20	0,232
	L	2	28,52	1,23	0,87	11,05	U - N	-0,72	0,473
	N	2	22,81	0,74	0,52	6,61	U - LN	1,20	0,232
	LN	2	9,31	0,07	0,05	0,64	L - V	1,20	0,232
	C	2	4,79	0,24	0,17	2,16	N - V	0,72	0,473
							LN - V	-1,20	0,232
							C - U	-1,67	0,094
							C - N	-2,39	**0,017**
							C - LN	-0,48	0,633
**TERC/CCL25 (46)**	U	2	166,58	17,00	12,02	152,73	U - V	-1,32	0,188
	V	2	321,67	12,65	8,95	113,66	U - L	-0,84	0,402
	L	2	235,95	26,40	18,67	237,16	U - N	0,24	0,811
	N	2	151,28	9,04	6,39	81,19	U - LN	1,08	0,282
	LN	2	55,09	6,68	4,73	60,04	L - V	-0,48	0,632
	C	2	45,07	0,59	0,42	5,34	N - V	-1,56	0,120
							LN - V	-2,39	**0,017**
							C - U	-1,56	0,120
							C - N	-1,32	0,188
							C - LN	-0,48	0,632
**TNFa (36)**	U	2	6,09	2,02	1,43	18,11	U - V	-1,20	0,232
	V	2	12,10	0,09	0,07	0,83	U - L	-1,67	0,094
	L	2	13,00	0,49	0,35	4,38	U - N	-0,72	0,473
	N	2	9,82	0,88	0,63	7,94	U - LN	0,96	0,339
	LN	2	2,35	0,60	0,43	5,40	L - V	0,48	0,633
	C	2	2,70	0,06	0,04	0,51	N - V	-0,48	0,633
							LN - V	-2,15	**0,031**
							C - U	-0,96	0,339
							C - N	-1,67	0,094

C - LN	0,00	1,000

SD: Standart deviation**, SE:** Standard Error**, CI:** Confidence Interval**, U:** Untreated**, V:** VX765**, L:** LPS**, N:** Nigericin**, LN:** LPS&Nigericin**, C:** Champtothesin
P value calculated using Kruskal-Wallis multiple comparison test

Supplemantary Table 4B: Cytokine secretion levels in MCF7 cells after VX765, LPS, Nigericin and LPS&Nigericin treatments

	Type	N	Mean	SD	SE	CI 95 %	**Kruskal-Wallis multiple comparison**	Z	P
**6Cine/CCL21 (12)**	U	2	6,61	0,13	0,10	1,21	U - V	0,48	0,632
	V	2	5,47	0,31	0,22	2,80	U - L	-1,44	0,151
	L	2	20,72	0,00	0,00	0,00	U - N	-0,66	0,510
	N	2	7,18	0,40	0,28	3,56	U - LN	-2,40	**0,017**
	LN	2	46,35	2,46	1,74	22,11	L - V	1,92	0,055
	C	2	7,27	0,52	0,37	4,70	N - V	1,14	0,255
							LN - V	2,87	**0,004**
							C - U	0,78	0,436
							C - N	0,12	0,905
							C - LN	-1,62	0,106
**BCA-1/CXCL13 (74)**	U	2	0,32	0,03	0,02	0,25	U - V	0,30	0,765
	V	2	0,31	0,01	0,01	0,13	U - L	-0,90	0,369
	L	2	0,48	0,02	0,02	0,19	U - N	1,02	0,309
	N	2	0,28	0,01	0,01	0,13	U - LN	-1,86	0,063
	LN	2	1,05	0,12	0,09	1,08	L - V	1,20	0,231
	C	2	0,33	0,01	0,01	0,06	N - V	-0,72	0,472
							LN - V	2,16	**0,031**
							C - U	0,12	0,905
							C - N	1,14	0,255
							C - LN	-1,74	0,082
**CTACK/CCL27 (72)**	U	2	0,45	0,03	0,02	0,25	U - V	-1,03	0,302
	V	2	0,51	0,01	0,01	0,13	U - L	-1,34	0,182
	L	2	0,53	0,04	0,03	0,32	U - N	-1,46	0,145
	N	2	0,52	0,00	0,00	0,00	U - LN	-2,91	**0,004**
	LN	2	1,15	0,14	0,10	1,27	L - V	0,30	0,761
	C	2	0,51	0,01	0,01	0,13	N - V	0,42	0,671
							LN - V	1,88	0,060
							C - U	1,03	0,302
							C - N	-0,42	0,671
							C - LN	-1,88	0,060
**ENA-78/CXCL5 (73)**	U	2	25,59	2,27	1,61	20,39	U - V	-1,73	0,083
	V	2	72,48	10,85	7,68	97,52	U - L	-0,96	0,338
	L	2	55,16	3,63	2,57	32,65	U - N	-1,62	0,106
	N	2	67,88	4,36	3,08	39,14	U - LN	-2,87	**0,004**
	LN	2	196,69	1,34	0,95	12,07	L - V	-0,78	0,437
	C	2	33,56	4,49	3,18	40,34	N - V	-0,12	0,905
							LN - V	1,14	0,256
							C - U	0,48	0,632
							C - N	-1,14	0,256
							C - LN	-2,39	**0,017**
**Eotaxin/CCL11 (43)**	U	2	4,10	0,12	0,09	1,08	U - V	0,00	1,000
	V	2	4,10	0,12	0,09	1,08	U - L	-1,51	0,130
	L	2	4,68	0,21	0,15	1,84	U - N	-0,85	0,396
	N	2	4,44	0,12	0,09	1,08	U - LN	-2,30	**0,021**
	LN	2	9,31	0,35	0,25	3,11	L - V	1,51	0,130
	C	2	4,01	0,00	0,00	0,00	N - V	0,85	0,396
							LN - V	2,30	**0,021**
							C - U	-0,36	0,716
							C - N	-1,21	0,226
							C - LN	-2,66	**0,008**
**Eotaxin2/CCL24 (30)**	U	2	1,73	0,33	0,23	2,92	U - V	-2,63	**0,009**
	V	2	4,27	0,31	0,22	2,80	U - L	0,00	1,000
	L	2	1,86	0,01	0,01	0,13	U - N	-2,03	**0,042**
	N	2	3,32	0,34	0,24	3,05	U - LN	-1,43	0,151
	LN	2	2,80	0,49	0,35	4,45	L - V	-2,63	**0,009**
	C	2	2,78	0,07	0,05	0,64	N - V	-0,60	0,550
							LN - V	-1,20	0,232
							C - U	1,43	0,151
							C - N	-0,60	0,550
							C - LN	0,00	1,000
**Eotaxin-3/CCL26 (65)**	U	2	1,20	0,08	0,06	0,70	U - V	-1,08	0,282
	V	2	1,63	0,04	0,03	0,32	U - L	-2,15	**0,031**
	L	2	2,78	0,36	0,26	3,24	U - N	-0,24	0,811
	N	2	1,30	0,18	0,13	1,65	U - LN	-2,75	**0,006**
	LN	2	4,30	0,28	0,20	2,54	L - V	1,08	0,282
	C	2	1,63	0,00	0,00	0,00	N - V	-0,84	0,402
							LN - V	1,68	0,094
							C - U	1,08	0,282

							C - N	0,84	0,402
							C - LN	-1,68	0,094
**Fractalkine?CX3CL1 (77)**	U	2	43,06	2,96	2,10	26,62	U - V	0,48	0,633
	V	2	35,11	1,25	0,89	11,24	U - L	-0,48	0,633
	L	2	291,51	1,99	1,41	17,85	U - N	1,43	0,151
	N	2	15,32	0,53	0,38	4,76	U - LN	-1,43	0,151
	LN	2	455,69	2,36	1,67	21,22	L - V	0,96	0,339
	C	2	22,21	1,61	1,14	14,42	N - V	-0,96	0,339
							LN - V	1,91	0,056
							C - U	-0,96	0,339
							C - N	0,48	0,633
							C - LN	-2,39	**0,017**
**GCP/CXCL6 (15)**	U	2	0,43	0,14	0,10	1,27	U - V	0,00	1,000
	V	2	0,43	0,14	0,10	1,27	U - L	-1,35	0,177
	L	2	1,63	0,01	0,01	0,13	U - N	-0,49	0,623
	N	2	0,53	0,00	0,00	0,00	U - LN	-2,33	**0,020**
	LN	2	13,17	1,18	0,84	10,61	L - V	1,35	0,177
	C	2	0,43	0,14	0,10	1,27	N - V	0,49	0,623
							LN - V	2,33	**0,020**
							C - U	0,00	1,000
							C - N	-0,49	0,623
							C - LN	-2,33	**0,020**
**GMCSF (34)**	U	2	1,83	0,79	0,56	7,12	U - V	-1,67	0,094
	V	2	14,70	0,09	0,07	0,83	U - L	-1,67	0,094
	L	2	13,38	2,09	1,48	18,74	U - N	-0,72	0,473
	N	2	7,53	0,18	0,13	1,59	U - LN	-2,87	**0,004**
	LN	2	33,48	1,53	1,08	13,72	L - V	0,00	1,000
	C	2	8,78	2,29	1,62	20,58	N - V	-0,96	0,339
							LN - V	1,20	0,232
							C - U	0,72	0,473
							C - N	0,00	1,000
							C - LN	-2,15	**0,031**
**Gro-a/CXCL1 (61)**	U	2	4,10	0,36	0,26	3,24	U - V	-0,90	0,370
	V	2	7,21	0,28	0,20	2,54	U - L	-1,73	0,083
	L	2	12,88	1,97	1,40	17,73	U - N	-1,50	0,135
	N	2	11,68	0,69	0,49	6,16	U - LN	-2,81	**0,005**
	LN	2	143,48	25,27	17,87	227,06	L - V	0,84	0,402
	C	2	4,83	0,67	0,48	6,04	N - V	0,60	0,550
							LN - V	1,91	0,056
							C - U	0,36	0,720
							C - N	-1,14	0,256
							C - LN	-2,45	**0,014**
**Gro-b/CXCL2 (78)**	U	2	2,42	0,21	0,15	1,91	U - V	0,00	1,000
	V	2	2,42	0,21	0,15	1,91	U - L	0,90	0,367
	L	2	2,16	0,00	0,00	0,00	U - N	-1,14	0,253
	N	2	6,17	0,27	0,19	2,41	U - LN	-1,99	**0,047**
	LN	2	42,88	2,80	1,98	25,16	L - V	-0,90	0,367
	C	2	2,70	0,61	0,43	5,46	N - V	1,14	0,253
							LN - V	1,99	**0,047**
							C - U	0,18	0,857
							C - N	-0,96	0,336
							C - LN	-1,80	0,071
**I-309/CCL1 (20)**	U	2	2,89	0,00	0,00	0,00	U - V	0,48	0,631
	V	2	2,75	0,00	0,00	0,00	U - L	-1,44	0,150
	L	2	4,30	0,00	0,00	0,00	U - N	-0,66	0,509
	N	2	3,36	0,08	0,06	0,76	U - LN	-2,40	**0,016**
	LN	2	10,27	0,08	0,06	0,70	L - V	1,92	0,055
	C	2	3,43	0,18	0,13	1,59	N - V	1,14	0,254
							LN - V	2,88	**0,004**
							C - U	0,78	0,435
							C - N	0,12	0,904
							C - LN	-1,62	0,105
**IFNg (21)**	U	2	0,08	0,00	0,00	0,00	U - V	0,60	0,547
	V	2	0,06	0,00	0,00	0,00	U - L	-1,32	0,186
	L	2	0,26	0,11	0,08	1,02	U - N	-0,24	0,810
	N	2	0,09	0,01	0,01	0,06	U - LN	-2,29	**0,022**
	LN	2	2,05	0,10	0,07	0,89	L - V	1,93	0,054
	C	2	0,10	0,00	0,00	0,00	N - V	0,84	0,400
							LN - V	2,89	**0,004**
							C - U	0,84	0,400
							C - N	0,60	0,547
							C - LN	-1,44	0,149
**IL1b (39)**	U	2	1,06	0,04	0,03	0,32	U - V	0,66	0,510
	V	2	0,57	0,10	0,07	0,89	U - L	-1,50	0,135
	L	2	73,01	0,16	0,11	1,40	U - N	-0,12	0,905
	N	2	1,11	0,11	0,08	0,95	U - LN	-2,21	**0,027**
	LN	2	174,24	10,77	7,62	96,76	L - V	2,15	**0,031**
	C	2	2,31	0,06	0,04	0,51	N - V	0,78	0,437
							LN - V	2,87	**0,004**
							C - U	0,78	0,437
							C - N	0,66	0,510

							C - LN	-1,44	0,151
**IL2 (38)**	U	2	0,37	0,03	0,02	0,25	U - V	-1,45	0,147
	V	2	0,51	0,00	0,00	0,00	U - L	0,48	0,628
	L	2	0,11	0,01	0,01	0,13	U - N	-0,73	0,468
	N	2	0,43	0,00	0,00	0,00	U - LN	-1,94	0,053
	LN	2	2,33	0,27	0,19	2,41	L - V	-1,94	0,053
	C	2	0,43	0,00	0,00	0,00	N - V	-0,73	0,468
							LN - V	0,48	0,628
							C - U	0,73	0,468
							C - N	0,00	1,000
							C - LN	-1,21	0,226
**IL4 (52)**	U	2	0,10	0,00	0,00	0,00	U - V	-1,44	0,151
	V	2	0,28	0,00	0,00	0,00	U - L	-1,92	0,055
	L	2	1,91	0,06	0,05	0,57	U - N	-0,96	0,338
	N	2	0,17	0,01	0,01	0,06	U - LN	-2,87	**0,004**
	LN	2	3,15	0,03	0,02	0,25	L - V	0,48	0,632
	C	2	0,15	0,01	0,01	0,06	N - V	-0,48	0,632
							LN - V	1,44	0,151
							C - U	0,48	0,632
							C - N	-0,48	0,632
							C - LN	-2,40	**0,017**
**IL6 (19)**	U	2	39,69	2,23	1,58	20,08	U - V	-0,48	0,633
	V	2	86,55	0,54	0,38	4,83	U - L	1,43	0,151
	L	2	17,01	0,69	0,49	6,16	U - N	0,48	0,633
	N	2	34,49	2,21	1,56	19,82	U - LN	-0,96	0,339
	LN	2	293,01	0,98	0,70	8,83	L - V	-1,91	0,056
	C	2	20,86	0,34	0,24	3,05	N - V	-0,96	0,339
							LN - V	0,48	0,633
							C - U	-0,96	0,339
							C - N	-0,48	0,633
							C - LN	-1,91	0,056
**IL8/CXCL8 (54)**	U	2	14,76	1,18	0,84	10,61	U - V	-1,79	0,073
	V	2	58,32	1,24	0,88	11,18	U - L	-0,96	0,339
	L	2	47,14	0,76	0,54	6,86	U - N	-2,39	**0,017**
	N	2	146,64	10,30	7,29	92,56	U - LN	-2,87	**0,004**
	LN	2	203,90	10,38	7,34	93,26	L - V	-0,84	0,403
	C	2	25,82	0,99	0,70	8,89	N - V	0,60	0,550
							LN - V	1,08	0,282
							C - U	0,48	0,633
							C - N	-1,91	0,056
							C - LN	-2,39	**0,017**
**IL10 (56)**	U	2	2,22	0,21	0,15	1,84	U - V	-0,24	0,811
	V	2	2,36	0,21	0,15	1,84	U - L	-1,67	0,094
	L	2	3,26	0,08	0,06	0,70	U - N	-1,43	0,151
	N	2	3,21	0,05	0,04	0,44	U - LN	-2,75	**0,006**
	LN	2	6,28	0,16	0,11	1,40	L - V	1,43	0,151
	C	2	2,63	0,13	0,09	1,14	N - V	1,20	0,232
							LN - V	2,51	**0,012**
							C - U	0,84	0,403
							C - N	-0,60	0,550
							C - LN	-1,91	0,056
**IL16 (27)**	U	2	2,36	0,22	0,16	1,97	U - V	-0,73	0,466
	V	2	2,67	0,22	0,16	1,97	U - L	-1,76	0,078
	L	2	4,47	0,00	0,00	0,00	U - N	-0,73	0,466
	N	2	2,67	0,22	0,16	1,97	U - LN	-2,73	**0,006**
	LN	2	29,31	1,79	1,27	16,07	L - V	1,03	0,302
	C	2	2,67	0,22	0,16	1,97	N - V	0,00	1,000
							LN - V	2,01	**0,045**
							C - U	0,73	0,466
							C - N	0,00	1,000
							C - LN	-2,01	**0,045**
**IP10/CXCL10 (48)**	U	2	4,14	0,01	0,01	0,13	U - V	1,91	0,056
	V	2	0,33	0,18	0,13	1,65	U - L	1,20	0,232
	L	2	1,17	0,01	0,01	0,13	U - N	1,91	0,056
	N	2	0,30	0,01	0,01	0,06	U - LN	-0,72	0,473
	LN	2	8,83	0,40	0,28	3,56	L - V	0,72	0,473
	C	2	1,55	0,11	0,08	1,02	N - V	0,00	1,000
							LN - V	2,63	**0,009**
							C - U	-0,72	0,473
							C - N	1,20	0,232
							C - LN	-1,43	0,151
**I-TAC/CXCL11 (25)**	U	2	1,14	0,00	0,00	0,00	U - V	1,08	0,279
	V	2	0,78	0,00	0,00	0,00	U - L	-0,84	0,400
	L	2	1,43	0,18	0,13	1,59	U - N	0,60	0,548
	N	2	0,88	0,07	0,05	0,64	U - LN	-1,80	0,071
	LN	2	4,07	0,32	0,23	2,86	L - V	1,92	0,054
	C	2	1,17	0,04	0,03	0,32	N - V	0,48	0,631
							LN - V	2,88	**0,004**
							C - U	0,24	0,810
							C - N	0,84	0,400
							C - LN	-1,56	0,118

**MCP-1/CCL2 (53)**	U	2	117,30	1,08	0,77	9,72	U - V	0,48	0,632
	V	2	96,68	1,92	1,36	17,22	U - L	-0,96	0,338
	L	2	257,56	3,65	2,58	32,78	U - N	1,26	0,209
	N	2	91,81	3,01	2,13	27,00	U - LN	-1,44	0,151
	LN	2	382,59	15,55	11,00	139,70	L - V	1,44	0,151
	C	2	92,81	1,58	1,12	14,23	N - V	-0,78	0,437
							LN - V	1,91	0,056
							C - U	-1,14	0,256
							C - N	0,12	0,905
							C - LN	-2,57	**0,010**
**MCP-2/CCL8 (57)**	U	2	0,13	0,01	0,01	0,13	U - V	-0,96	0,338
	V	2	0,17	0,01	0,01	0,06	U - L	-1,44	0,151
	L	2	0,21	0,02	0,02	0,19	U - N	0,42	0,675
	N	2	0,12	0,01	0,01	0,06	U - LN	-2,40	**0,017**
	LN	2	0,44	0,04	0,03	0,32	L - V	0,48	0,632
	C	2	0,15	0,01	0,01	0,06	N - V	-1,38	0,168
							LN - V	1,44	0,151
							C - U	0,42	0,675
							C - N	0,84	0,402
							C - LN	-1,98	**0,048**
**MCP-3/CCL7 (26)**	U	2	1,98	0,45	0,32	4,07	U - V	0,84	0,400
	V	2	1,50	0,23	0,16	2,03	U - L	-1,02	0,306
	L	2	43,16	1,49	1,06	13,41	U - N	-0,18	0,857
	N	2	2,06	0,11	0,08	1,02	U - LN	-1,99	**0,047**
	LN	2	113,19	4,16	2,95	37,42	L - V	1,86	0,062
	C	2	1,98	0,00	0,00	0,00	N - V	1,02	0,306
							LN - V	2,83	**0,005**
							C - U	-0,06	0,952
							C - N	-0,24	0,810
							C - LN	-2,05	**0,041**
**MCP-4/CCL13 (28)**	U	2	0,26	0,04	0,03	0,32	U - V	-0,96	0,338
	V	2	0,57	0,06	0,05	0,57	U - L	-1,44	0,151
	L	2	0,73	0,00	0,00	0,00	U - N	-2,15	**0,031**
	N	2	1,20	0,02	0,02	0,19	U - LN	-2,87	**0,004**
	LN	2	2,99	0,13	0,09	1,14	L - V	0,48	0,632
	C	2	0,39	0,01	0,01	0,06	N - V	1,20	0,231
							LN - V	1,91	0,056
							C - U	0,48	0,632
							C - N	-1,68	0,094
							C - LN	-2,39	**0,017**
**MDC/CCL22 (29)**	U	2	56,70	2,71	1,92	24,33	U - V	0,96	0,339
	V	2	10,69	0,12	0,09	1,08	U - L	-1,43	0,151
	L	2	402,75	8,61	6,09	77,38	U - N	1,43	0,151
	N	2	5,67	0,12	0,09	1,08	U - LN	-0,96	0,339
	LN	2	252,69	6,87	4,86	61,75	L - V	2,39	**0,017**
	C	2	21,33	0,16	0,12	1,46	N - V	-0,48	0,633
							LN - V	1,91	0,056
							C - U	-0,48	0,633
							C - N	0,96	0,339
							C - LN	-1,43	0,151
**MIF (35)**	U	2	584,13	1,75	1,24	15,69	U - V	1,43	0,151
	V	2	166,07	2,19	1,55	19,69	U - L	0,96	0,339
	L	2	364,40	9,01	6,37	80,94	U - N	0,48	0,633
	N	2	414,11	0,28	0,20	2,54	U - LN	-0,96	0,339
	LN	2	823,38	24,22	17,13	217,59	L - V	0,48	0,633
	C	2	1273,74	62,90	44,48	565,11	N - V	0,96	0,339
							LN - V	2,39	**0,017**
							C - U	1,43	0,151
							C - N	1,91	0,056
							C - LN	0,48	0,633
**MIG/CXCL9 (14)**	U	2	3,81	0,16	0,11	1,40	U - V	0,49	0,623
	V	2	3,70	0,00	0,00	0,00	U - L	-1,11	0,269
	L	2	9,66	0,16	0,11	1,40	U - N	0,00	1,000
	N	2	3,81	0,16	0,11	1,40	U - LN	-2,09	**0,037**
	LN	2	15,74	1,14	0,81	10,23	L - V	1,60	0,110
	C	2	3,81	0,16	0,11	1,40	N - V	0,49	0,623
							LN - V	2,58	**0,010**
							C - U	0,00	1,000
							C - N	0,00	1,000
							C - LN	-2,09	**0,037**
**MIP-1a/CCL3 (55)**	U	2	0,40	0,00	0,00	0,00	U - V	-1,08	0,279
	V	2	0,46	0,06	0,04	0,51	U - L	0,48	0,631
	L	2	0,36	0,01	0,01	0,13	U - N	-0,84	0,400
	N	2	0,44	0,02	0,02	0,19	U - LN	-1,92	0,054
	LN	2	0,85	0,07	0,05	0,64	L - V	-1,56	0,118
	C	2	0,45	0,04	0,03	0,38	N - V	-0,24	0,810
							LN - V	0,84	0,400
							C - U	0,96	0,336
							C - N	0,12	0,904
							C - LN	-0,96	0,336
**MIP1b/CCL15 (66)**	U	2	2,33	0,39	0,28	3,49	U - V	0,00	1,000

	V	2	2,40	0,18	0,13	1,65	U - L	-1,55	0,120
	L	2	10,02	0,50	0,36	4,51	U - N	1,20	0,232
	N	2	1,65	0,16	0,11	1,40	U - LN	-1,08	0,282
	LN	2	6,96	0,24	0,17	2,16	L - V	1,55	0,120
	C	2	1,78	0,13	0,09	1,14	N - V	-1,20	0,232
							LN - V	1,08	0,282
							C - U	-0,96	0,339
							C - N	0,24	0,811
							C - LN	-2,03	**0,042**
**MIP-3a/CCL20 (62)**	U	2	1,40	0,03	0,02	0,25	U - V	0,48	0,633
	V	2	0,60	0,36	0,26	3,24	U - L	-0,96	0,339
	L	2	4,15	0,18	0,13	1,65	U - N	-0,48	0,633
	N	2	1,55	0,01	0,01	0,13	U - LN	-2,39	**0,017**
	LN	2	75,58	1,65	1,17	14,87	L - V	1,43	0,151
	C	2	4,82	0,43	0,31	3,88	N - V	0,96	0,339
							LN - V	2,87	**0,004**
							C - U	1,43	0,151
							C - N	0,96	0,339
							C - LN	-0,96	0,339
**MIP3-b/CCL19 (76)**	U	2	0,92	0,04	0,03	0,32	U - V	-0,48	0,633
	V	2	1,15	0,07	0,05	0,64	U - L	-0,96	0,339
	L	2	4,73	0,06	0,04	0,51	U - N	0,48	0,633
	N	2	0,85	0,01	0,01	0,13	U - LN	-1,91	0,056
	LN	2	10,05	0,23	0,16	2,03	L - V	0,48	0,633
	C	2	0,80	0,01	0,01	0,13	N - V	-0,96	0,339
							LN - V	1,43	0,151
							C - U	-0,96	0,339
							C - N	-0,48	0,633
							C - LN	-2,87	**0,004**
**MPIF-1/CCL23 (37)**	U	2	0,58	0,00	0,00	0,00	U - V	-0,86	0,391
	V	2	0,74	0,00	0,00	0,00	U - L	-2,14	**0,032**
	L	2	2,21	0,83	0,59	7,50	U - N	-0,86	0,391
	N	2	0,74	0,00	0,00	0,00	U - LN	-2,94	**0,003**
	LN	2	7,25	0,18	0,13	1,59	L - V	1,29	0,198
	C	2	0,82	0,11	0,08	1,02	N - V	0,00	1,000
							LN - V	2,08	**0,037**
							C - U	1,23	0,220
							C - N	0,37	0,713
							C - LN	-1,72	0,086
**SCYB16/CXCL16 (64)**	U	2	76,40	4,97	3,52	44,66	U - V	1,43	0,151
	V	2	38,85	1,94	1,37	17,41	U - L	-0,96	0,339
	L	2	149,31	4,41	3,12	39,58	U - N	1,91	0,056
	N	2	2,08	0,15	0,11	1,33	U - LN	-0,48	0,633
	LN	2	87,78	5,25	3,71	47,14	L - V	2,39	**0,017**
	C	2	46,81	1,03	0,73	9,28	N - V	-0,48	0,633
							LN - V	1,91	0,056
							C - U	-0,96	0,339
							C - N	0,96	0,339
							C - LN	-1,43	0,151
**SDF-1ab/CXCL12 (22)**	U	2	5,71	0,00	0,00	0,00	U - V	0,48	0,631
	V	2	0,80	0,06	0,05	0,57	U - L	-0,96	0,337
	L	2	24,25	0,72	0,51	6,48	U - N	1,02	0,308
	N	2	0,71	0,01	0,01	0,13	U - LN	-1,44	0,150
	LN	2	33,15	0,00	0,00	0,00	L - V	1,44	0,150
	C	2	0,69	0,01	0,01	0,13	N - V	-0,54	0,589
							LN - V	1,92	0,055
							C - U	-1,38	0,168
							C - N	-0,36	0,719
							C - LN	-2,82	**0,005**
**TARC/CCL17 (67)**	U	2	0,59	0,14	0,10	1,27	U - V	-0,80	0,426
	V	2	0,78	0,13	0,09	1,14	U - L	-1,72	0,086
	L	2	2,60	0,00	0,00	0,00	U - N	-0,37	0,713
	N	2	0,69	0,00	0,00	0,00	U - LN	-2,70	**0,007**
	LN	2	11,10	0,55	0,39	4,96	L - V	0,92	0,358
	C	2	0,78	0,13	0,09	1,14	N - V	-0,43	0,668
							LN - V	1,90	0,058
							C - U	0,80	0,426
							C - N	0,43	0,668
							C - LN	-1,90	0,058
**TERC/CCL25 (46)**	U	2	11,77	0,26	0,19	2,35	U - V	-0,12	0,905
	V	2	11,95	0,52	0,37	4,64	U - L	-0,78	0,436
	L	2	13,77	0,44	0,31	3,94	U - N	-1,44	0,151
	N	2	16,44	1,70	1,20	15,25	U - LN	-2,70	**0,007**
	LN	2	62,49	2,01	1,42	18,04	L - V	0,66	0,510
	C	2	16,99	0,92	0,65	8,26	N - V	1,32	0,188
							LN - V	2,58	**0,010**
							C - U	1,56	0,119
							C - N	0,12	0,905
							C - LN	-1,14	0,255
**TNFa (36)**	U	2	7,31	0,40	0,28	3,56	U - V	-0,48	0,632
	V	2	13,38	0,82	0,58	7,37	U - L	0,48	0,632

Type	N	Mean	SD	SE	CI 95 %	Kruskal-Wallis multiple comparison	Z	P
L	2	3,24	0,24	0,17	2,16	U - N	-1,97	**0,048**
N	2	44,72	2,04	1,45	18,36	U - LN	-1,44	0,151
LN	2	36,80	7,56	5,35	67,91	L - V	-0,96	0,338
C	2	47,22	1,50	1,06	13,47	N - V	1,50	0,135
						LN - V	0,96	0,338
						C - U	2,33	**0,020**
						C - N	0,36	0,720
						C - LN	0,90	0,370

SD: Standart deviation**, SE:** Standard Error**, CI:** Confidence Interval**, U:** Untreated**, V:** VX765**, L:** LPS**, N:** Nigericin**, LN:** LPS&Nigericin**, C:** Champtothesin
P value calculated using Kruskal-Wallis multiple comparison test

Supplemantary Table 4C: Cytokine secretion levels in PC3 cells after VX765, LPS, Nigericin and LPS&Nigericin treatments

	Type	N	Mean	SD	SE	CI 95 %	**Kruskal-Wallis multiple comparison**	Z	P
**6Cine/CCL21 (12)**	U	2	36,31	1,45	1,03	13,02	U - V	0,24	0,811
	V	2	35,54	0,12	0,09	1,08	U - L	-1,67	0,094
	L	2	74,67	2,04	1,44	18,30	U - N	-0,24	0,811
	N	2	36,93	0,23	0,17	2,10	U - LN	-2,39	**0,017**
	LN	2	79,33	0,64	0,45	5,72	L - V	1,91	0,056
	C	2	40,57	0,66	0,47	5,91	N - V	0,48	0,633
							LN - V	2,63	**0,009**
							C - U	0,96	0,339
							C - N	0,72	0,473
							C - LN	-1,43	0,151
**BCA-1/CXCL13 (74)**	U	2	2,11	0,04	0,03	0,38	U - V	0,96	0,339
	V	2	1,87	0,01	0,01	0,06	U - L	-1,31	0,189
	L	2	3,75	0,03	0,02	0,25	U - N	0,48	0,633
	N	2	2,04	0,01	0,01	0,13	U - LN	-1,91	0,056
	LN	2	4,10	0,42	0,30	3,81	L - V	2,27	**0,023**
	C	2	2,39	0,13	0,09	1,14	N - V	0,48	0,633
							LN - V	2,87	**0,004**
							C - U	0,48	0,633
							C - N	0,96	0,339
							C - LN	-1,43	0,151
**CTACK/CCL27 (72)**	U	2	3,66	0,08	0,06	0,70	U - V	0,00	1,000
	V	2	3,68	0,02	0,02	0,19	U - L	1,38	0,168
	L	2	3,34	0,04	0,03	0,32	U - N	-1,02	0,309
	N	2	4,09	0,10	0,07	0,89	U - LN	0,30	0,765
	LN	2	3,63	0,38	0,27	3,43	L - V	-1,38	0,168
	C	2	4,28	0,16	0,12	1,46	N - V	1,02	0,309
							LN - V	-0,30	0,765
							C - U	1,38	0,168
							C - N	0,36	0,719
							C - LN	1,68	0,094
**ENA-78/CXCL5 (73)**	U	2	1058,72	18,46	13,05	165,82	U - V	0,00	1,000
	V	2	1051,82	55,42	39,19	497,96	U - L	1,43	0,151
	L	2	945,95	65,09	46,03	584,80	U - N	0,24	0,811
	N	2	1043,59	16,53	11,69	148,54	U - LN	1,31	0,189
	LN	2	964,19	3,00	2,12	26,94	L - V	-1,43	0,151
	C	2	1101,31	20,39	14,42	183,16	N - V	-0,24	0,811
							LN - V	-1,31	0,189
							C - U	0,72	0,473
							C - N	0,96	0,339
							C - LN	2,03	**0,042**
**Eotaxin/CCL11 (43)**	U	2	20,74	0,54	0,38	4,83	U - V	0,48	0,633
	V	2	19,18	0,08	0,06	0,70	U - L	-1,55	0,120
	L	2	24,07	0,69	0,49	6,23	U - N	-0,72	0,473
	N	2	23,37	0,13	0,10	1,21	U - LN	-2,39	**0,017**
	LN	2	31,86	2,37	1,68	21,28	L - V	2,03	**0,042**
	C	2	22,87	0,98	0,69	8,77	N - V	1,20	0,232
							LN - V	2,87	**0,004**
							C - U	0,72	0,473
							C - N	0,00	1,000
							C - LN	-1,67	0,094
**Eotaxin2/CCL24 (30)**	U	2	5,30	0,95	0,67	8,51	U - V	0,12	0,905
	V	2	4,92	0,19	0,14	1,72	U - L	-0,24	0,811
	L	2	5,44	1,01	0,72	9,08	U - N	1,55	0,120
	N	2	3,77	0,42	0,30	3,75	U - LN	0,36	0,720
	LN	2	4,93	0,47	0,34	4,26	L - V	0,36	0,720
	C	2	5,75	1,20	0,85	10,80	N - V	-1,43	0,151
							LN - V	-0,24	0,811
							C - U	0,72	0,473
							C - N	2,27	**0,023**
							C - LN	1,08	0,282
**Eotaxin-3/CCL26 (65)**	U	2	11,69	1,25	0,89	11,24	U - V	1,31	0,189

	V	2	7,32	0,36	0,26	3,24	U - L	-1,08	0,282
	L	2	14,10	0,30	0,21	2,67	U - N	1,79	0,073
	N	2	6,25	0,35	0,25	3,11	U - LN	0,36	0,720
	LN	2	10,75	2,05	1,45	18,42	L - V	2,39	**0,017**
	C	2	11,20	0,98	0,69	8,77	N - V	-0,48	0,633
							LN - V	0,96	0,339
							C - U	-0,36	0,720
							C - N	1,43	0,151
							C - LN	0,00	1,000
**Fractalkine?CX3CL1 (77)**	U	2	585,75	0,61	0,43	5,46	U - V	0,48	0,633
	V	2	346,39	0,79	0,56	7,12	U - L	-1,67	0,094
	L	2	816,95	4,15	2,94	37,29	U - N	0,96	0,339
	N	2	331,81	9,07	6,42	81,51	U - LN	-1,20	0,232
	LN	2	791,95	181,89	128,62	1634,21	L - V	2,15	**0,031**
	C	2	728,83	49,62	35,09	445,86	N - V	-0,48	0,633
							LN - V	1,67	0,094
							C - U	0,84	0,403
							C - N	1,79	0,073
							C - LN	-0,36	0,720
**GCP/CXCL6 (15)**	U	2	6152,33	1632,64	1154,45	14668,68	U - V	0,48	0,633
	V	2	2527,63	25,94	18,35	233,10	U - L	-0,96	0,339
	L	2	16372,36	2779,97	1965,74	24977,03	U - N	0,96	0,339
	N	2	816,20	2,86	2,02	25,67	U - LN	1,43	0,151
	LN	2	681,67	145,69	103,02	1308,93	L - V	1,43	0,151
	C	2	9574,00	134,55	95,14	1208,87	N - V	-0,48	0,633
							LN - V	-0,96	0,339
							C - U	0,48	0,633
							C - N	1,43	0,151
							C - LN	1,91	0,056
**GMCSF (34)**	U	2	157,40	5,96	4,22	53,56	U - V	-0,48	0,633
	V	2	211,45	9,55	6,75	85,77	U - L	-0,96	0,339
	L	2	226,87	2,15	1,52	19,31	U - N	-1,43	0,151
	N	2	238,93	3,68	2,61	33,10	U - LN	-2,39	**0,017**
	LN	2	1004,13	128,04	90,54	1150,36	L - V	0,48	0,633
	C	2	78,65	10,20	7,22	91,68	N - V	0,96	0,339
							LN - V	1,91	0,056
							C - U	-0,48	0,633
							C - N	-1,91	0,056
							C - LN	-2,87	**0,004**
**Gro-a/CXCL1 (61)**	U	2	5161,15	3999,12	2827,81	35930,67	U - V	0,36	0,720
	V	2	2769,86	67,90	48,01	610,02	U - L	0,36	0,720
	L	2	2748,83	633,55	447,99	5692,19	U - N	1,31	0,189
	N	2	2010,45	7,88	5,57	70,77	U - LN	1,79	0,073
	LN	2	1837,73	187,69	132,72	1686,30	L - V	0,00	1,000
	C	2	3690,39	912,08	644,94	8194,74	N - V	-0,96	0,339
							LN - V	-1,43	0,151
							C - U	0,24	0,811
							C - N	1,55	0,120
							C - LN	2,03	**0,042**
**Gro-b/CXCL2 (78)**	U	2	969,42	29,22	20,66	262,51	U - V	-0,48	0,633
	V	2	1098,19	27,54	19,48	247,45	U - L	0,96	0,339
	L	2	719,46	14,69	10,39	131,95	U - N	-1,43	0,151
	N	2	2384,38	227,17	160,63	2041,00	U - LN	-1,91	0,056
	LN	2	17557,14	17793,35	12581,80	159866,90	L - V	-1,43	0,151
	C	2	769,68	0,64	0,46	5,78	N - V	0,96	0,339
							LN - V	1,43	0,151
							C - U	-0,48	0,633
							C - N	-1,91	0,056
							C - LN	-2,39	**0,017**
**I-309/CCL1 (20)**	U	2	18,15	0,00	0,00	0,00	U - V	0,48	0,632
	V	2	17,08	0,08	0,06	0,76	U - L	-1,91	0,056
	L	2	21,73	0,18	0,13	1,59	U - N	-0,48	0,632
	N	2	19,20	0,10	0,07	0,89	U - LN	-2,39	**0,017**
	LN	2	23,75	2,02	1,43	18,17	L - V	2,39	**0,017**
	C	2	20,26	0,10	0,07	0,89	N - V	0,96	0,338
							LN - V	2,87	**0,004**
							C - U	0,96	0,338
							C - N	0,48	0,632
							C - LN	-1,44	0,151
**IFNg (21)**	U	2	1,51	0,08	0,06	0,76	U - V	0,84	0,402
	V	2	1,36	0,01	0,01	0,13	U - L	-1,38	0,169
	L	2	5,61	0,06	0,04	0,51	U - N	0,24	0,811
	N	2	1,46	0,03	0,02	0,25	U - LN	-1,56	0,120
	LN	2	5,79	0,60	0,43	5,40	L - V	2,21	**0,027**
	C	2	1,74	0,05	0,04	0,44	N - V	0,60	0,550
							LN - V	2,39	**0,017**
							C - U	0,60	0,550
							C - N	0,84	0,402
							C - LN	-0,96	0,338
**IL1b (39)**	U	2	122,90	6,03	4,27	54,19	U - V	1,43	0,151
	V	2	70,79	0,94	0,67	8,45	U - L	-1,08	0,282

	L	2	544,44	10,67	7,55	95,87	U - N	0,96	0,339
	N	2	95,30	0,91	0,64	8,13	U - LN	-0,48	0,633
	LN	2	473,49	45,87	32,44	412,13	L - V	2,51	**0,012**
	C	2	110,37	5,59	3,95	50,19	N - V	0,48	0,633
							LN - V	1,91	0,056
							C - U	-0,48	0,633
							C - N	0,48	0,633
							C - LN	-0,96	0,339
**IL2 (38)**	U	2	4,58	0,49	0,35	4,38	U - V	1,20	0,232
	V	2	3,15	0,01	0,01	0,13	U - L	-0,84	0,403
	L	2	12,38	0,57	0,40	5,08	U - N	0,72	0,473
	N	2	3,73	0,33	0,23	2,92	U - LN	-1,55	0,120
	LN	2	13,29	0,40	0,29	3,62	L - V	2,03	**0,042**
	C	2	4,60	0,38	0,27	3,43	N - V	0,48	0,633
							LN - V	2,75	**0,006**
							C - U	0,00	1,000
							C - N	0,72	0,473
							C - LN	-1,55	0,120
**IL4 (52)**	U	2	4,41	0,30	0,22	2,73	U - V	0,72	0,473
	V	2	4,09	0,00	0,00	0,00	U - L	-1,20	0,231
	L	2	7,63	0,04	0,03	0,38	U - N	0,00	1,000
	N	2	4,52	0,08	0,06	0,76	U - LN	-2,15	**0,031**
	LN	2	10,44	0,45	0,32	4,00	L - V	1,91	0,056
	C	2	5,17	0,11	0,08	1,02	N - V	0,72	0,473
							LN - V	2,87	**0,004**
							C - U	0,72	0,473
							C - N	0,72	0,473
							C - LN	-1,44	0,151
**IL6 (19)**	U	2	103,34	7,86	5,56	70,65	U - V	0,48	0,633
	V	2	83,26	0,46	0,33	4,13	U - L	0,96	0,339
	L	2	39,95	2,84	2,01	25,48	U - N	-1,55	0,120
	N	2	3311,68	263,74	186,50	2369,64	U - LN	-1,79	0,073
	LN	2	4091,82	1278,10	903,76	11483,30	L - V	-0,48	0,633
	C	2	288,55	7,58	5,36	68,11	N - V	2,03	**0,042**
							LN - V	2,27	**0,023**
							C - U	0,48	0,633
							C - N	-1,08	0,282
							C - LN	-1,31	0,189
**IL8/CXCL8 (54)**	U	2	11767,61	33,70	23,83	302,79	U - V	-1,20	0,232
	V	2	12787,73	513,19	362,88	4610,83	U - L	-0,36	0,720
	L	2	11759,42	1540,33	1089,18	13839,28	U - N	-0,72	0,473
	N	2	12351,86	265,58	187,79	2386,10	U - LN	0,12	0,905
	LN	2	11374,26	1446,10	1022,55	12992,73	L - V	-0,84	0,403
	C	2	13391,60	22,21	15,71	199,55	N - V	-0,48	0,633
							LN - V	-1,31	0,189
							C - U	2,15	**0,031**
							C - N	1,43	0,151
							C - LN	2,27	**0,023**
**IL10 (56)**	U	2	29,52	0,04	0,03	0,32	U - V	1,02	0,309
	V	2	28,06	0,07	0,05	0,64	U - L	-1,73	0,083
	L	2	34,15	1,03	0,73	9,28	U - N	0,12	0,905
	N	2	29,47	0,11	0,08	0,95	U - LN	-1,50	0,135
	LN	2	33,66	0,46	0,33	4,13	L - V	2,75	**0,006**
	C	2	30,67	0,88	0,62	7,88	N - V	0,90	0,370
							LN - V	2,51	**0,012**
							C - U	0,66	0,510
							C - N	0,78	0,437
							C - LN	-0,84	0,402
**IL16 (27)**	U	2	47,46	2,33	1,65	20,97	U - V	0,60	0,550
	V	2	43,20	1,53	1,09	13,79	U - L	-1,55	0,120
	L	2	130,04	4,14	2,93	37,23	U - N	0,00	1,000
	N	2	47,81	6,22	4,40	55,84	U - LN	-1,91	0,056
	LN	2	141,42	18,58	13,14	166,90	L - V	2,15	**0,031**
	C	2	52,21	1,89	1,34	16,96	N - V	0,60	0,550
							LN - V	2,51	**0,012**
							C - U	0,60	0,550
							C - N	0,60	0,550
							C - LN	-1,31	0,189
**IP10/CXCL10 (48)**	U	2	32,85	0,54	0,39	4,89	U - V	0,96	0,339
	V	2	16,11	0,05	0,04	0,44	U - L	2,39	**0,017**
	L	2	3,45	0,02	0,02	0,19	U - N	1,43	0,151
	N	2	15,37	0,69	0,49	6,16	U - LN	1,91	0,056
	LN	2	4,44	0,27	0,19	2,41	L - V	-1,43	0,151
	C	2	16,92	1,03	0,73	9,28	N - V	-0,48	0,633
							LN - V	-0,96	0,339
							C - U	-0,48	0,633
							C - N	0,96	0,339
							C - LN	1,43	0,151
**I-TAC/CXCL11 (25)**	U	2	38,16	6,23	4,41	55,97	U - V	1,43	0,151
	V	2	12,47	0,47	0,34	4,26	U - L	2,87	**0,004**
	L	2	9,06	0,20	0,14	1,78	U - N	0,84	0,403

	N	2	20,25	0,33	0,23	2,92	U - LN	1,91	0,056
	LN	2	10,93	0,64	0,46	5,78	L - V	-1,43	0,151
	C	2	20,34	0,37	0,27	3,37	N - V	0,60	0,550
							LN - V	-0,48	0,633
							C - U	-0,60	0,550
							C - N	0,24	0,811
							C - LN	1,31	0,189
**MCP-1/CCL2 (53)**	U	2	15,12	0,03	0,02	0,25	U - V	0,48	0,633
	V	2	13,07	0,45	0,32	4,00	U - L	-0,48	0,633
	L	2	28,54	1,67	1,18	14,99	U - N	2,39	**0,017**
	N	2	4,24	0,14	0,10	1,27	U - LN	0,96	0,339
	LN	2	12,18	0,76	0,54	6,86	L - V	0,96	0,339
	C	2	6,22	0,45	0,32	4,07	N - V	-1,91	0,056
							LN - V	-0,48	0,633
							C - U	-1,91	0,056
							C - N	0,48	0,633
							C - LN	-0,96	0,339
**MCP-2/CCL8 (57)**	U	2	0,75	0,03	0,02	0,25	U - V	0,48	0,632
	V	2	0,71	0,00	0,00	0,00	U - L	-1,62	0,106
	L	2	0,97	0,05	0,04	0,44	U - N	-0,48	0,632
	N	2	0,82	0,01	0,01	0,13	U - LN	-2,16	**0,031**
	LN	2	1,05	0,13	0,10	1,21	L - V	2,10	**0,036**
	C	2	0,92	0,03	0,02	0,25	N - V	0,96	0,338
							LN - V	2,64	0,008
							C - U	1,20	0,231
							C - N	0,72	0,472
							C - LN	-0,96	0,338
**MCP-3/CCL7 (26)**	U	2	37,16	1,23	0,87	11,05	U - V	1,20	0,231
	V	2	26,44	2,67	1,89	24,01	U - L	-1,32	0,188
	L	2	200,99	6,42	4,54	57,69	U - N	0,72	0,473
	N	2	31,62	0,73	0,52	6,54	U - LN	-1,32	0,188
	LN	2	201,36	8,68	6,14	77,95	L - V	2,51	**0,012**
	C	2	36,99	0,00	0,00	0,00	N - V	0,48	0,632
							LN - V	2,51	**0,012**
							C - U	0,00	1,000
							C - N	0,72	0,473
							C - LN	-1,32	0,188
**MCP-4/CCL13 (28)**	U	2	67,82	1,01	0,72	9,08	U - V	0,36	0,720
	V	2	65,50	2,28	1,61	20,46	U - L	1,67	0,094
	L	2	39,25	7,18	5,08	64,48	U - N	1,08	0,282
	N	2	51,06	0,01	0,01	0,13	U - LN	1,91	0,056
	LN	2	33,55	2,52	1,78	22,62	L - V	-1,31	0,189
	C	2	67,46	1,82	1,29	16,33	N - V	-0,72	0,473
							LN - V	-1,55	0,120
							C - U	0,00	1,000
							C - N	1,08	0,282
							C - LN	1,91	0,056
**MDC/CCL22 (29)**	U	2	5,89	0,05	0,04	0,44	U - V	0,90	0,369
	V	2	5,23	0,00	0,00	0,00	U - L	-1,02	0,309
	L	2	6,44	0,19	0,14	1,72	U - N	0,36	0,719
	N	2	5,80	0,07	0,05	0,64	U - LN	-1,98	**0,048**
	LN	2	7,77	0,52	0,37	4,64	L - V	1,92	0,055
	C	2	6,37	0,02	0,02	0,19	N - V	0,54	0,590
							LN - V	2,87	**0,004**
							C - U	1,02	0,309
							C - N	1,38	0,168
							C - LN	-0,96	0,338
**MIF (35)**	U	2	486,05	75,28	53,23	676,35	U - V	0,84	0,403
	V	2	320,39	66,04	46,70	593,38	U - L	0,24	0,811
	L	2	438,66	13,62	9,63	122,36	U - N	-1,55	0,120
	N	2	1603,60	105,53	74,62	948,14	U - LN	-0,60	0,550
	LN	2	656,10	112,52	79,57	1010,97	L - V	0,60	0,550
	C	2	1006,71	89,07	62,99	800,30	N - V	2,39	**0,017**
							LN - V	1,43	0,151
							C - U	1,08	0,282
							C - N	-0,48	0,633
							C - LN	0,48	0,633
**MIG/CXCL9 (14)**	U	2	19,75	0,32	0,23	2,86	U - V	0,24	0,811
	V	2	18,95	1,34	0,95	12,01	U - L	-1,56	0,120
	L	2	33,56	0,57	0,40	5,08	U - N	-0,60	0,550
	N	2	22,17	0,05	0,04	0,44	U - LN	-2,51	**0,012**
	LN	2	37,80	1,32	0,94	11,88	L - V	1,79	0,073
	C	2	24,11	0,62	0,44	5,53	N - V	0,84	0,402
							LN - V	2,75	**0,006**
							C - U	1,08	0,282
							C - N	0,48	0,632
							C - LN	-1,44	0,151
**MIP-1a/CCL3 (55)**	U	2	1,90	0,11	0,08	1,02	U - V	-1,02	0,309
	V	2	2,05	0,02	0,02	0,19	U - L	-2,09	**0,036**
	L	2	4,13	0,16	0,12	1,46	U - N	-0,12	0,905
	N	2	1,96	0,03	0,02	0,25	U - LN	-2,69	**0,007**

	LN	2	5,27	0,69	0,49	6,16	L - V	1,08	0,282
	C	2	2,14	0,21	0,15	1,84	N - V	-0,90	0,370
							LN - V	1,68	0,094
							C - U	1,02	0,309
							C - N	0,90	0,370
							C - LN	-1,68	0,094
**MIP1b/CCL15 (66)**	U	2	9,87	0,88	0,62	7,88	U - V	1,32	0,188
	V	2	7,55	0,26	0,19	2,35	U - L	-1,08	0,282
	L	2	13,31	0,69	0,49	6,23	U - N	0,54	0,590
	N	2	8,49	0,16	0,11	1,40	U - LN	-1,32	0,188
	LN	2	14,13	1,07	0,76	9,66	L - V	2,39	**0,017**
	C	2	7,95	0,62	0,44	5,53	N - V	0,78	0,437
							LN - V	2,63	**0,008**
							C - U	-1,02	0,309
							C - N	-0,48	0,632
							C - LN	-2,33	**0,020**
**MIP-3a/CCL20 (62)**	U	2	1016,75	142,96	101,09	1284,47	U - V	1,55	0,120
	V	2	346,71	8,70	6,15	78,14	U - L	-0,36	0,720
	L	2	1118,85	155,58	110,02	1397,87	U - N	1,55	0,120
	N	2	346,01	0,38	0,27	3,43	U - LN	-0,12	0,905
	LN	2	1332,76	1336,31	944,92	12006,28	L - V	1,91	0,056
	C	2	1274,41	139,07	98,34	1249,53	N - V	0,00	1,000
							LN - V	1,67	0,094
							C - U	0,72	0,473
							C - N	2,27	**0,023**
							C - LN	0,60	0,550
**MIP3-b/CCL19 (76)**	U	2	12,03	0,10	0,07	0,89	U - V	0,60	0,550
	V	2	11,62	0,30	0,21	2,67	U - L	-1,43	0,151
	L	2	40,85	5,89	4,17	52,92	U - N	0,12	0,905
	N	2	12,33	0,86	0,61	7,75	U - LN	-1,43	0,151
	LN	2	42,95	2,68	1,90	24,08	L - V	2,03	**0,042**
	C	2	15,06	0,81	0,58	7,31	N - V	0,48	0,633
							LN - V	2,03	**0,042**
							C - U	0,72	0,473
							C - N	0,84	0,403
							C - LN	-0,72	0,473
**MPIF-1/CCL23 (37)**	U	2	8,70	0,23	0,17	2,10	U - V	0,72	0,473
	V	2	7,57	0,41	0,29	3,68	U - L	-1,68	0,094
	L	2	17,85	0,00	0,00	0,00	U - N	0,00	1,000
	N	2	8,58	0,47	0,34	4,26	U - LN	-2,15	**0,031**
	LN	2	19,19	1,69	1,20	15,18	L - V	2,39	**0,017**
	C	2	9,48	0,66	0,47	5,91	N - V	0,72	0,473
							LN - V	2,87	**0,004**
							C - U	0,72	0,473
							C - N	0,72	0,473
							C - LN	-1,44	0,151
**SCYB16/CXCL16 (64)**	U	2	28,36	0,33	0,23	2,92	U - V	1,20	0,232
	V	2	18,74	0,35	0,25	3,18	U - L	0,96	0,339
	L	2	19,59	1,99	1,41	17,92	U - N	2,87	**0,004**
	N	2	4,39	0,10	0,07	0,89	U - LN	2,15	**0,031**
	LN	2	5,95	0,71	0,50	6,35	L - V	0,24	0,811
	C	2	20,20	0,33	0,24	2,99	N - V	-1,67	0,094
							LN - V	-0,96	0,339
							C - U	-0,72	0,473
							C - N	2,15	**0,031**
							C - LN	1,43	0,151
**SDF-1ab/CXCL12 (22)**	U	2	40,07	0,00	0,00	0,00	U - V	0,72	0,472
	V	2	36,67	0,71	0,50	6,35	U - L	-1,86	0,063
	L	2	49,22	0,83	0,59	7,43	U - N	-0,48	0,632
	N	2	43,29	1,40	0,99	12,58	U - LN	-1,68	0,094
	LN	2	49,31	3,92	2,78	35,26	L - V	2,58	**0,010**
	C	2	41,78	4,29	3,04	38,56	N - V	1,20	0,231
							LN - V	2,40	**0,017**
							C - U	0,24	0,811
							C - N	-0,24	0,811
							C - LN	-1,44	0,151
**TARC/CCL17 (67)**	U	2	10,42	0,52	0,37	4,70	U - V	0,12	0,905
	V	2	10,12	0,10	0,07	0,89	U - L	-1,62	0,105
	L	2	24,96	0,00	0,00	0,00	U - N	-0,90	0,368
	N	2	12,35	0,00	0,00	0,00	U - LN	-2,58	**0,010**
	LN	2	32,55	1,77	1,26	15,95	L - V	1,74	0,082
	C	2	12,51	0,34	0,24	3,05	N - V	1,02	0,308
							LN - V	2,70	**0,007**
							C - U	0,90	0,368
							C - N	0,00	1,000
							C - LN	-1,68	0,093
**TERC/CCL25 (46)**	U	2	221,95	0,91	0,64	8,13	U - V	0,96	0,339
	V	2	177,57	2,30	1,63	20,65	U - L	-1,20	0,232
	L	2	251,39	2,74	1,94	24,59	U - N	0,48	0,633
	N	2	210,74	1,11	0,79	9,97	U - LN	-1,91	0,056
	LN	2	294,65	6,40	4,53	57,50	L - V	2,15	**0,031**

	Type	N	Mean	SD	SE	CI 95 %	Kruskal-Wallis multiple comparison	Z	P
	C	2	239,45	12,03	8,51	108,07	N - V	0,48	0,633
							LN - V	2,87	**0,004**
							C - U	0,60	0,550
							C - N	1,08	0,282
							C - LN	-1,31	0,189
**TNFa (36)**	U	2	12,62	1,19	0,84	10,67	U - V	-0,48	0,632
	V	2	14,68	0,00	0,00	0,00	U - L	-0,96	0,338
	L	2	17,18	0,09	0,07	0,83	U - N	-1,91	0,056
	N	2	37,20	1,13	0,80	10,16	U - LN	-2,63	**0,008**
	LN	2	129,64	47,48	33,57	426,55	L - V	0,48	0,632
	C	2	29,21	1,75	1,24	15,69	N - V	1,44	0,151
							LN - V	2,15	**0,031**
							C - U	1,44	0,151
							C - N	-0,48	0,632
							C - LN	-1,20	0,231

SD: Standart deviation**, SE:** Standard Error**, CI:** Confidence Interval**, U:** Untreated**, V:** VX765**, L:** LPS**, N:** Nigericin**, LN:** LPS&Nigericin**, C:** Champtothesin

P value calculated using Kruskal-Wallis multiple comparison test

Supplemantary Table 4D: Cytokine secretion levels in SH-SY5Y cells after VX765, LPS, Nigericin and LPS&Nigericin treatments

	Type	N	Mean	SD	SE	CI 95 %	**Kruskal-Wallis multiple comparison**	Z	P
**6Cine/CCL21 (12)**	U	2	2,33	0,48	0,34	4,32	U - V	0,00	1,000
	V	2	2,33	0,48	0,34	4,32	U - L	-2,02	**0,043**
	L	2	5,41	0,13	0,09	1,14	U - N	0,18	0,854
	N	2	2,17	0,25	0,18	2,22	U - LN	-1,04	0,298
	LN	2	5,00	0,14	0,10	1,27	L - V	2,02	**0,043**
	C	2	1,99	0,00	0,00	0,00	N - V	-0,18	0,854
							LN - V	1,04	0,298
							C - U	-0,55	0,582
							C - N	-0,37	0,714
							C - LN	-1,59	0,112
**BCA-1/CXCL13 (74)**	U	2	0,25	0,00	0,00	0,00	U - V	1,22	0,224
	V	2	0,23	0,00	0,00	0,00	U - L	-1,34	0,181
	L	2	0,45	0,02	0,02	0,19	U - N	1,22	0,224
	N	2	0,23	0,00	0,00	0,00	U - LN	-0,49	0,626
	LN	2	0,37	0,03	0,02	0,25	L - V	2,56	**0,011**
	C	2	0,24	0,00	0,00	0,00	N - V	0,00	1,000
							LN - V	1,70	0,088
							C - U	-0,49	0,626
							C - N	0,73	0,465
							C - LN	-0,97	0,330
**CTACK/CCL27 (72)**	U	2	0,43	0,00	0,00	0,00	U - V	0,85	0,395
	V	2	0,37	0,04	0,03	0,32	U - L	-1,40	0,163
	L	2	0,53	0,04	0,03	0,32	U - N	0,85	0,395
	N	2	0,37	0,04	0,03	0,32	U - LN	-0,49	0,627
	LN	2	0,45	0,00	0,00	0,00	L - V	2,25	**0,025**
	C	2	0,34	0,00	0,00	0,00	N - V	0,00	1,000
							LN - V	1,34	0,182
							C - U	-1,21	0,225
							C - N	-0,36	0,716
							C - LN	-1,70	0,089
**ENA-78/CXCL5 (73)**	U	2	33,56	0,00	0,00	0,00	U - V	0,42	0,674
	V	2	28,77	6,77	4,79	60,86	U - L	1,80	0,071
	L	2	17,80	8,06	5,70	72,43	U - N	0,18	0,857
	N	2	30,36	9,02	6,38	81,00	U - LN	2,04	**0,041**
	LN	2	12,40	0,14	0,10	1,27	L - V	-1,38	0,167
	C	2	12,57	2,34	1,66	21,03	N - V	0,24	0,810
							LN - V	-1,62	0,105
							C - U	-2,04	**0,041**
							C - N	-1,86	0,062
							C - LN	0,00	1,000
**Eotaxin/CCL11 (43)**	U	2	3,01	0,13	0,10	1,21	U - V	2,16	**0,031**
	V	2	2,41	0,15	0,11	1,33	U - L	-0,30	0,764
	L	2	3,17	0,16	0,11	1,40	U - N	1,68	0,092
	N	2	2,51	0,29	0,21	2,60	U - LN	1,44	0,149
	LN	2	2,61	0,00	0,00	0,00	L - V	2,46	**0,014**
	C	2	2,61	0,14	0,10	1,27	N - V	0,48	0,631
							LN - V	0,72	0,471
							C - U	-1,44	0,149
							C - N	0,24	0,810
							C - LN	0,00	1,000
**Eotaxin2/CCL24 (30)**	U	2	3,39	0,22	0,16	1,97	U - V	0,72	0,473
	V	2	2,75	0,06	0,05	0,57	U - L	2,09	**0,036**
	L	2	0,88	0,04	0,03	0,38	U - N	0,36	0,720
	N	2	3,01	0,33	0,24	2,99	U - LN	2,15	**0,031**

	LN	2	0,86	0,12	0,09	1,08	L - V	-1,38	0,169
	C	2	1,83	0,01	0,01	0,13	N - V	0,36	0,720
							LN - V	-1,44	0,151
							C - U	-1,32	0,188
							C - N	-0,96	0,338
							C - LN	0,84	0,402
**Eotaxin-3/CCL26 (65)**	U	2	1,06	0,00	0,00	0,00	U - V	2,52	**0,012**
	V	2	0,73	0,04	0,03	0,32	U - L	0,06	0,952
	L	2	1,07	0,08	0,06	0,76	U - N	1,86	0,063
	N	2	0,82	0,01	0,01	0,13	U - LN	1,32	0,187
	LN	2	0,86	0,04	0,03	0,38	L - V	2,46	**0,014**
	C	2	0,79	0,10	0,07	0,89	N - V	0,66	0,509
							LN - V	1,20	0,230
							C - U	-1,92	0,055
							C - N	-0,06	0,952
							C - LN	-0,60	0,548
**Fractalkine?CX3CL1 (77)**	U	2	1,81	0,04	0,03	0,32	U - V	1,21	0,226
	V	2	1,59	0,12	0,09	1,08	U - L	-0,91	0,364
	L	2	44,12	2,86	2,03	25,73	U - N	0,67	0,506
	N	2	1,73	0,08	0,06	0,70	U - LN	-1,39	0,164
	LN	2	48,60	3,54	2,50	31,77	L - V	2,12	**0,034**
	C	2	1,78	0,00	0,00	0,00	N - V	0,54	0,586
							LN - V	2,60	**0,009**
							C - U	-0,30	0,762
							C - N	0,36	0,717
							C - LN	-1,69	0,090
**GCP/CXCL6 (15)**	U	2	0,53	0,00	0,00	0,00	U - V	0,97	0,333
	V	2	0,10	0,01	0,01	0,06	U - L	-1,39	0,164
	L	2	1,86	0,18	0,13	1,59	U - N	0,67	0,505
	N	2	0,31	0,32	0,23	2,86	U - LN	-0,79	0,431
	LN	2	1,72	0,02	0,02	0,19	L - V	2,36	**0,018**
	C	2	0,43	0,14	0,10	1,27	N - V	0,30	0,762
							LN - V	1,76	0,079
							C - U	-0,30	0,762
							C - N	0,36	0,716
							C - LN	-1,09	0,276
**GMCSF (34)**	U	2	11,10	0,27	0,19	2,41	U - V	1,62	0,105
	V	2	5,94	1,37	0,97	12,33	U - L	-0,90	0,368
	L	2	14,84	2,04	1,44	18,30	U - N	0,78	0,436
	N	2	9,65	1,78	1,26	16,01	U - LN	-0,48	0,631
	LN	2	13,36	3,13	2,22	28,14	L - V	2,52	**0,012**
	C	2	6,56	2,59	1,83	23,25	N - V	0,84	0,401
							LN - V	2,10	**0,036**
							C - U	-1,56	0,119
							C - N	-0,78	0,436
							C - LN	-2,04	**0,041**
**Gro-a/CXCL1 (61)**	U	2	1,74	0,52	0,37	4,64	U - V	0,96	0,339
	V	2	0,87	0,01	0,01	0,06	U - L	-0,60	0,550
	L	2	10,63	0,18	0,13	1,65	U - N	0,48	0,633
	N	2	0,96	0,01	0,01	0,13	U - LN	-1,43	0,151
	LN	2	11,27	0,10	0,07	0,89	L - V	1,55	0,120
	C	2	0,84	0,01	0,01	0,06	N - V	0,48	0,633
							LN - V	2,39	**0,017**
							C - U	-1,43	0,151
							C - N	-0,96	0,339
							C - LN	-2,87	**0,004**
**Gro-b/CXCL2 (78)**	U	2	1,77	0,25	0,18	2,29	U - V	0,88	0,381
	V	2	1,59	0,00	0,00	0,00	U - L	-0,75	0,453
	L	2	1,90	0,37	0,27	3,37	U - N	0,00	1,000
	N	2	1,77	0,25	0,18	2,29	U - LN	0,75	0,453
	LN	2	1,47	0,23	0,16	2,03	L - V	1,63	0,104
	C	2	1,59	0,00	0,00	0,00	N - V	0,88	0,381
							LN - V	0,13	0,900
							C - U	-0,88	0,381
							C - N	-0,88	0,381
							C - LN	-0,13	0,900
**I-309/CCL1 (20)**	U	2	2,23	0,11	0,08	0,95	U - V	1,15	0,248
	V	2	1,90	0,12	0,09	1,08	U - L	-1,40	0,162
	L	2	3,96	0,16	0,12	1,46	U - N	1,03	0,302
	N	2	1,94	0,06	0,04	0,51	U - LN	-0,61	0,543
	LN	2	3,60	0,00	0,00	0,00	L - V	2,55	**0,011**
	C	2	1,98	0,00	0,00	0,00	N - V	0,12	0,903
							LN - V	1,76	0,078
							C - U	-0,73	0,466
							C - N	0,30	0,761
							C - LN	-1,34	0,181
**IFNg (21)**	U	2	0,05	0,01	0,01	0,13	U - V	0,06	0,951
	V	2	0,05	0,00	0,00	0,00	U - L	-1,66	0,097
	L	2	0,11	0,01	0,01	0,06	U - N	-0,37	0,712
	N	2	0,06	0,01	0,01	0,06	U - LN	-1,91	0,057
	LN	2	0,12	0,03	0,02	0,25	L - V	1,72	0,085

	C	2	0,05	0,00	0,00	0,00	N - V	0,43	0,667
							LN - V	1,97	**0,049**
							C - U	-0,06	0,951
							C - N	-0,43	0,667
							C - LN	-1,97	**0,049**
**IL1b (39)**	U	2	0,48	0,04	0,03	0,32	U - V	0,96	0,337
	V	2	0,39	0,04	0,03	0,32	U - L	-1,20	0,230
	L	2	61,13	5,70	4,03	51,21	U - N	0,72	0,471
	N	2	0,41	0,06	0,05	0,57	U - LN	-1,44	0,150
	LN	2	63,86	0,37	0,27	3,37	L - V	2,16	**0,031**
	C	2	0,46	0,06	0,05	0,57	N - V	0,24	0,810
							LN - V	2,40	**0,016**
							C - U	-0,24	0,810
							C - N	0,48	0,631
							C - LN	-1,68	0,093
**IL2 (38)**	U	2	0,34	0,02	0,02	0,19	U - V	0,67	0,505
	V	2	0,30	0,03	0,02	0,25	U - L	1,46	0,146
	L	2	0,11	0,01	0,01	0,06	U - N	-0,36	0,716
	N	2	0,35	0,00	0,00	0,00	U - LN	2,30	**0,021**
	LN	2	0,07	0,00	0,00	0,00	L - V	-0,79	0,431
	C	2	0,30	0,03	0,02	0,25	N - V	1,03	0,303
							LN - V	-1,64	0,102
							C - U	-0,67	0,505
							C - N	-1,03	0,303
							C - LN	1,64	0,102
**IL4 (52)**	U	2	0,15	0,01	0,01	0,06	U - V	0,96	0,335
	V	2	0,11	0,01	0,01	0,06	U - L	-0,84	0,399
	L	2	1,55	0,06	0,05	0,57	U - N	0,00	1,000
	N	2	0,15	0,01	0,01	0,06	U - LN	-1,39	0,166
	LN	2	1,64	0,06	0,05	0,57	L - V	1,81	0,071
	C	2	0,11	0,03	0,02	0,25	N - V	0,96	0,335
							LN - V	2,35	**0,019**
							C - U	-0,96	0,335
							C - N	-0,96	0,335
							C - LN	-2,35	**0,019**
**IL6 (19)**	U	2	0,65	0,02	0,02	0,19	U - V	0,12	0,904
	V	2	0,63	0,04	0,03	0,38	U - L	0,85	0,398
	L	2	0,37	0,12	0,09	1,08	U - N	-0,60	0,546
	N	2	0,69	0,04	0,03	0,38	U - LN	1,45	0,147
	LN	2	0,10	0,00	0,00	0,00	L - V	-0,72	0,469
	C	2	0,78	0,00	0,00	0,00	N - V	0,72	0,469
							LN - V	-1,33	0,184
							C - U	1,33	0,184
							C - N	0,72	0,469
							C - LN	2,78	**0,005**
**IL8/CXCL8 (54)**	U	2	1,18	0,04	0,03	0,32	U - V	0,72	0,473
	V	2	0,69	0,19	0,14	1,72	U - L	-0,96	0,339
	L	2	9,21	0,04	0,03	0,32	U - N	1,20	0,232
	N	2	0,40	0,05	0,04	0,44	U - LN	-1,67	0,094
	LN	2	16,39	2,34	1,66	21,03	L - V	1,67	0,094
	C	2	1,11	0,21	0,15	1,84	N - V	-0,48	0,633
							LN - V	2,39	**0,017**
							C - U	0,00	1,000
							C - N	1,20	0,232
							C - LN	-1,67	0,094
**IL10 (56)**	U	2	1,50	0,06	0,04	0,51	U - V	1,45	0,148
	V	2	1,22	0,00	0,00	0,00	U - L	-1,26	0,206
	L	2	3,25	0,23	0,16	2,03	U - N	0,60	0,547
	N	2	1,34	0,06	0,04	0,51	U - LN	-0,48	0,630
	LN	2	2,41	0,00	0,00	0,00	L - V	2,71	**0,007**
	C	2	1,30	0,00	0,00	0,00	N - V	0,84	0,399
							LN - V	1,93	0,054
							C - U	-0,84	0,399
							C - N	-0,24	0,810
							C - LN	-1,32	0,185
**IL16 (27)**	U	2	2,05	0,21	0,15	1,91	U - V	1,40	0,161
	V	2	1,30	0,00	0,00	0,00	U - L	-1,16	0,247
	L	2	3,77	0,99	0,70	8,89	U - N	0,85	0,393
	N	2	1,60	0,42	0,30	3,81	U - LN	0,06	0,951
	LN	2	2,35	1,02	0,72	9,15	L - V	2,56	**0,010**
	C	2	1,90	0,00	0,00	0,00	N - V	0,55	0,583
							LN - V	1,34	0,180
							C - U	-0,30	0,760
							C - N	0,55	0,583
							C - LN	-0,24	0,807
**IP10/CXCL10 (48)**	U	2	0,20	0,03	0,02	0,25	U - V	0,42	0,673
	V	2	0,18	0,01	0,01	0,06	U - L	-0,66	0,508
	L	2	0,80	0,03	0,02	0,25	U - N	0,84	0,399
	N	2	0,16	0,01	0,01	0,13	U - LN	-0,90	0,366
	LN	2	0,82	0,00	0,00	0,00	L - V	1,08	0,278
	C	2	0,14	0,00	0,00	0,00	N - V	-0,42	0,673

							LN - V	1,32	0,185
							C - U	-1,39	0,166
							C - N	-0,54	0,588
							C - LN	-2,29	**0,022**
**I-TAC/CXCL11 (25)**	U	2	0,61	0,02	0,02	0,19	U - V	0,90	0,366
	V	2	0,53	0,01	0,01	0,13	U - L	-1,81	0,071
	L	2	1,26	0,06	0,05	0,57	U - N	0,12	0,904
	N	2	0,60	0,04	0,03	0,32	U - LN	-1,08	0,278
	LN	2	1,08	0,06	0,05	0,57	L - V	2,71	**0,007**
	C	2	0,57	0,07	0,05	0,64	N - V	0,78	0,434
							LN - V	1,99	**0,047**
							C - U	-0,42	0,673
							C - N	-0,30	0,763
							C - LN	-1,51	0,132
**MCP-1/CCL2 (53)**	U	2	0,23	0,00	0,00	0,00	U - V	0,73	0,468
	V	2	0,20	0,01	0,01	0,06	U - L	-1,21	0,226
	L	2	0,50	0,00	0,00	0,00	U - N	1,21	0,226
	N	2	0,17	0,01	0,01	0,06	U - LN	0,48	0,628
	LN	2	0,17	0,10	0,07	0,89	L - V	1,94	0,053
	C	2	0,23	0,00	0,00	0,00	N - V	-0,48	0,628
							LN - V	0,24	0,809
							C - U	0,00	1,000
							C - N	1,21	0,226
							C - LN	0,48	0,628
**MCP-2/CCL8 (57)**	U	2	0,14	0,01	0,01	0,06	U - V	-0,48	0,631
	V	2	0,16	0,01	0,01	0,06	U - L	-1,44	0,150
	L	2	0,61	0,04	0,03	0,32	U - N	0,84	0,401
	N	2	0,10	0,01	0,01	0,06	U - LN	-0,96	0,337
	LN	2	0,40	0,03	0,02	0,25	L - V	0,96	0,337
	C	2	0,10	0,00	0,00	0,00	N - V	-1,32	0,187
							LN - V	0,48	0,631
							C - U	-0,60	0,548
							C - N	0,24	0,810
							C - LN	-1,56	0,119
**MCP-3/CCL7 (26)**	U	2	1,02	0,00	0,00	0,00	U - V	0,00	1,000
	V	2	1,02	0,00	0,00	0,00	U - L	-1,15	0,251
	L	2	33,71	2,12	1,50	19,06	U - N	0,00	1,000
	N	2	1,02	0,00	0,00	0,00	U - LN	-2,17	**0,030**
	LN	2	37,76	0,16	0,11	1,40	L - V	1,15	0,251
	C	2	0,86	0,23	0,16	2,03	N - V	0,00	1,000
							LN - V	2,17	**0,030**
							C - U	-0,51	0,610
							C - N	-0,51	0,610
							C - LN	-2,68	**0,007**
**MCP-4/CCL13 (28)**	U	2	0,14	0,01	0,01	0,13	U - V	1,04	0,298
	V	2	0,11	0,00	0,00	0,00	U - L	-1,23	0,220
	L	2	0,46	0,01	0,01	0,13	U - N	0,61	0,540
	N	2	0,12	0,01	0,01	0,13	U - LN	-1,35	0,178
	LN	2	0,48	0,04	0,03	0,32	L - V	2,27	**0,023**
	C	2	0,11	0,00	0,00	0,00	N - V	0,43	0,668
							LN - V	2,39	**0,017**
							C - U	-1,04	0,298
							C - N	-0,43	0,668
							C - LN	-2,39	**0,017**
**MDC/CCL22 (29)**	U	2	1,89	0,00	0,00	0,00	U - V	0,79	0,429
	V	2	1,73	0,07	0,05	0,64	U - L	2,01	**0,045**
	L	2	1,59	0,12	0,09	1,08	U - N	0,91	0,361
	N	2	1,71	0,04	0,03	0,32	U - LN	2,80	**0,005**
	LN	2	1,39	0,00	0,00	0,00	L - V	-1,22	0,224
	C	2	1,68	0,00	0,00	0,00	N - V	-0,12	0,903
							LN - V	-2,01	**0,045**
							C - U	-1,22	0,224
							C - N	-0,30	0,761
							C - LN	1,58	0,114
**MIF (35)**	U	2	404,49	12,20	8,63	109,59	U - V	0,72	0,473
	V	2	356,54	2,53	1,79	22,74	U - L	-0,48	0,633
	L	2	801,48	20,97	14,83	188,43	U - N	1,67	0,094
	N	2	214,67	42,43	30,00	381,19	U - LN	-0,96	0,339
	LN	2	950,50	6,14	4,35	55,21	L - V	1,20	0,232
	C	2	216,39	22,32	15,79	200,57	N - V	-0,96	0,339
							LN - V	1,67	0,094
							C - U	-1,67	0,094
							C - N	0,00	1,000
							C - LN	-2,63	**0,009**
**MIG/CXCL9 (14)**	U	2	2,82	0,00	0,00	0,00	U - V	0,37	0,714
	V	2	2,71	0,16	0,12	1,46	U - L	-1,83	0,067
	L	2	8,60	0,00	0,00	0,00	U - N	0,06	0,951
	N	2	2,82	0,33	0,23	2,92	U - LN	-1,10	0,272
	LN	2	8,10	0,00	0,00	0,00	L - V	2,20	**0,028**
	C	2	2,59	0,33	0,24	2,99	N - V	0,31	0,760
							LN - V	1,47	0,143

							C - U	-0,55	0,583
							C - N	-0,49	0,625
							C - LN	-1,65	0,099
**MIP-1a/CCL3 (55)**	U	2	0,35	0,01	0,01	0,13	U - V	0,60	0,545
	V	2	0,33	0,02	0,02	0,19	U - L	1,27	0,204
	L	2	0,29	0,01	0,01	0,06	U - N	-0,18	0,856
	N	2	0,36	0,02	0,02	0,19	U - LN	2,00	**0,046**
	LN	2	0,24	0,04	0,03	0,38	L - V	-0,67	0,506
	C	2	0,36	0,02	0,02	0,19	N - V	0,79	0,432
							LN - V	-1,39	0,164
							C - U	0,18	0,856
							C - N	0,00	1,000
							C - LN	2,18	**0,029**
**MIP1b/CCL15 (66)**	U	2	1,18	0,00	0,00	0,00	U - V	1,09	0,277
	V	2	1,03	0,00	0,00	0,00	U - L	-1,69	0,091
	L	2	1,82	0,00	0,00	0,00	U - N	0,24	0,809
	N	2	1,16	0,03	0,02	0,25	U - LN	-0,91	0,365
	LN	2	1,65	0,08	0,06	0,70	L - V	2,78	**0,005**
	C	2	1,16	0,13	0,09	1,14	N - V	0,85	0,398
							LN - V	1,99	**0,046**
							C - U	-0,12	0,904
							C - N	0,12	0,904
							C - LN	-1,03	0,304
**MIP-3a/CCL20 (62)**	U	2	0,27	0,04	0,03	0,32	U - V	0,66	0,509
	V	2	0,23	0,01	0,01	0,13	U - L	-1,56	0,118
	L	2	0,74	0,01	0,01	0,13	U - N	0,48	0,631
	N	2	0,24	0,01	0,01	0,06	U - LN	-0,72	0,471
	LN	2	0,56	0,04	0,03	0,32	L - V	2,22	**0,026**
	C	2	0,21	0,01	0,01	0,13	N - V	0,18	0,857
							LN - V	1,38	0,167
							C - U	-1,26	0,207
							C - N	-0,78	0,435
							C - LN	-1,98	**0,047**
**MIP3-b/CCL19 (76)**	U	2	0,68	0,01	0,01	0,06	U - V	0,48	0,632
	V	2	0,51	0,03	0,02	0,25	U - L	-2,21	**0,027**
	L	2	3,67	0,30	0,22	2,73	U - N	-0,72	0,473
	N	2	0,77	0,10	0,07	0,89	U - LN	-1,44	0,151
	LN	2	3,10	0,13	0,09	1,14	L - V	2,69	**0,007**
	C	2	0,80	0,01	0,01	0,13	N - V	1,20	0,231
							LN - V	1,91	0,056
							C - U	0,72	0,473
							C - N	0,00	1,000
							C - LN	-0,72	0,473
**MPIF-1/CCL23 (37)**	U	2	0,41	0,00	0,00	0,00	U - V	0,60	0,545
	V	2	0,23	0,00	0,00	0,00	U - L	-1,03	0,304
	L	2	0,58	0,11	0,08	0,95	U - N	0,36	0,717
	N	2	0,30	0,28	0,20	2,54	U - LN	-1,33	0,183
	LN	2	0,63	0,04	0,03	0,32	L - V	1,63	0,102
	C	2	0,17	0,09	0,07	0,83	N - V	0,24	0,809
							LN - V	1,94	0,053
							C - U	-0,91	0,364
							C - N	-0,54	0,586
							C - LN	-2,24	**0,025**
**SCYB16/CXCL16 (64)**	U	2	0,33	0,01	0,01	0,13	U - V	1,45	0,148
	V	2	0,23	0,01	0,01	0,06	U - L	-1,33	0,185
	L	2	0,49	0,03	0,02	0,25	U - N	0,84	0,399
	N	2	0,25	0,00	0,00	0,00	U - LN	-0,72	0,469
	LN	2	0,44	0,05	0,04	0,44	L - V	2,77	**0,006**
	C	2	0,27	0,03	0,02	0,25	N - V	0,60	0,547
							LN - V	2,17	**0,030**
							C - U	-0,60	0,547
							C - N	0,24	0,809
							C - LN	-1,33	0,185
**SDF-1ab/CXCL12 (22)**	U	2	0,73	0,04	0,03	0,32	U - V	0,84	0,399
	V	2	0,59	0,13	0,09	1,14	U - L	-0,96	0,335
	L	2	12,96	0,63	0,45	5,65	U - N	1,21	0,228
	N	2	0,49	0,04	0,03	0,38	U - LN	-0,60	0,547
	LN	2	12,51	0,00	0,00	0,00	L - V	1,81	0,070
	C	2	0,56	0,06	0,05	0,57	N - V	-0,36	0,718
							LN - V	1,45	0,148
							C - U	-0,84	0,399
							C - N	0,36	0,718
							C - LN	-1,45	0,148
**TARC/CCL17 (67)**	U	2	0,22	0,01	0,01	0,13	U - V	1,02	0,308
	V	2	0,15	0,01	0,01	0,13	U - L	-1,44	0,150
	L	2	2,39	0,00	0,00	0,00	U - N	0,72	0,472
	N	2	0,18	0,04	0,03	0,32	U - LN	-0,48	0,631
	LN	2	1,19	0,76	0,54	6,80	L - V	2,46	**0,014**
	C	2	0,13	0,04	0,03	0,38	N - V	0,30	0,764
							LN - V	1,50	0,134
							C - U	-1,14	0,255

							C - N	-0,42	0,675
							C - LN	-1,62	0,105
**TERC/CCL25 (46)**	U	2	6,15	0,52	0,37	4,64	U - V	0,00	1,000
	V	2	6,15	0,52	0,37	4,64	U - L	-1,80	0,072
	L	2	15,95	1,76	1,25	15,82	U - N	0,36	0,719
	N	2	5,88	0,39	0,28	3,49	U - LN	-1,32	0,188
	LN	2	10,68	0,44	0,31	3,94	L - V	1,80	0,072
	C	2	5,24	0,26	0,19	2,35	N - V	-0,36	0,719
							LN - V	1,32	0,188
							C - U	-1,08	0,281
							C - N	-0,72	0,472
							C - LN	-2,40	**0,017**
**TNFa (36)**	U	2	0,63	0,04	0,03	0,32	U - V	1,06	0,287
	V	2	0,54	0,04	0,03	0,32	U - L	2,25	**0,024**
	L	2	0,02	0,00	0,00	0,00	U - N	0,38	0,707
	N	2	0,60	0,00	0,00	0,00	U - LN	2,25	**0,024**
	LN	2	0,02	0,00	0,00	0,00	L - V	-1,19	0,234
	C	2	0,57	0,08	0,06	0,76	N - V	0,69	0,491
							LN - V	-1,19	0,234
							C - U	-0,56	0,573
							C - N	-0,19	0,851
							C - LN	1,69	0,091

SD: Standart deviation**, SE:** Standard Error**, CI:** Confidence Interval**, U:** Untreated**, V:** VX765**, L:** LPS**, N:** Nigericin**, LN:** LPS&Nigericin**, C:** Champtothesin
P value calculated using Kruskal-Wallis multiple comparison test

Supplemantary Table 4E: Cytokine secretion levels in U138MG cells after VX765, LPS, Nigericin and LPS&Nigericin treatments

	Type	N	Mean	SD	SE	CI 95 %	**Kruskal-Wallis multiple comparison**	Z	P
**6Cine/CCL21 (12)**	U	2	20,16	0,78	0,55	6,99	U - V	0,84	0,402
	V	2	11,83	0,41	0,29	3,68	U - L	-0,96	0,338
	L	2	71,30	2,14	1,52	19,25	U - N	1,44	0,151
	N	2	11,24	0,00	0,00	0,00	U - LN	-0,48	0,632
	LN	2	60,44	2,08	1,47	18,68	L - V	1,79	0,073
	C	2	12,26	0,60	0,43	5,40	N - V	-0,60	0,550
							LN - V	1,32	0,188
							C - U	-0,60	0,550
							C - N	0,84	0,402
							C - LN	-1,08	0,282
**BCA-1/CXCL13 (74)**	U	2	0,58	0,01	0,01	0,13	U - V	1,44	0,151
	V	2	0,41	0,00	0,00	0,00	U - L	-0,48	0,632
	L	2	1,21	0,08	0,06	0,76	U - N	0,48	0,632
	N	2	0,50	0,01	0,01	0,06	U - LN	-0,96	0,338
	LN	2	1,44	0,08	0,06	0,70	L - V	1,91	0,056
	C	2	0,45	0,03	0,02	0,25	N - V	0,96	0,338
							LN - V	2,39	**0,017**
							C - U	-0,96	0,338
							C - N	-0,48	0,632
							C - LN	-1,91	0,056
**CTACK/CCL27 (72)**	U	2	1,31	0,08	0,06	0,76	U - V	1,26	0,209
	V	2	0,85	0,01	0,01	0,13	U - L	-0,48	0,632
	L	2	1,99	0,13	0,10	1,21	U - N	0,48	0,632
	N	2	1,06	0,04	0,03	0,32	U - LN	-0,96	0,338
	LN	2	2,27	0,13	0,09	1,14	L - V	1,73	0,083
	C	2	0,89	0,06	0,05	0,57	N - V	0,78	0,437
							LN - V	2,21	**0,027**
							C - U	-1,14	0,256
							C - N	-0,66	0,510
							C - LN	-2,09	**0,036**
**ENA-78/CXCL5 (73)**	U	2	485,92	22,03	15,58	197,96	U - V	0,48	0,633
	V	2	232,64	1,03	0,73	9,28	U - L	-0,96	0,339
	L	2	830,71	7,88	5,57	70,77	U - N	0,96	0,339
	N	2	185,70	3,13	2,21	28,08	U - LN	-0,48	0,633
	LN	2	756,92	45,57	32,22	409,39	L - V	1,43	0,151
	C	2	133,01	8,46	5,99	76,05	N - V	-0,48	0,633
							LN - V	0,96	0,339
							C - U	-1,43	0,151
							C - N	-0,48	0,633
							C - LN	-1,91	0,056
**Eotaxin/CCL11 (43)**	U	2	12,30	0,54	0,39	4,89	U - V	1,32	0,187
	V	2	8,50	0,37	0,27	3,37	U - L	-0,96	0,337
	L	2	22,46	0,37	0,27	3,37	U - N	1,08	0,280
	N	2	8,76	0,00	0,00	0,00	U - LN	-0,48	0,631
	LN	2	19,75	1,07	0,76	9,59	L - V	2,28	**0,023**
	C	2	10,54	0,13	0,10	1,21	N - V	0,24	0,810
							LN - V	1,80	0,072

							C - U	-0,48	0,631
							C - N	0,60	0,548
							C - LN	-0,96	0,337
**Eotaxin2/CCL24 (30)**	U	2	2,81	0,25	0,18	2,29	U - V	1,31	0,189
	V	2	1,50	0,09	0,07	0,83	U - L	0,72	0,473
	L	2	2,48	0,19	0,14	1,72	U - N	-1,08	0,282
	N	2	3,29	0,08	0,06	0,76	U - LN	-0,60	0,550
	LN	2	3,18	0,01	0,01	0,06	L - V	0,60	0,550
	C	2	2,61	0,11	0,08	0,95	N - V	2,39	**0,017**
							LN - V	1,91	0,056
							C - U	-0,36	0,720
							C - N	-1,43	0,151
							C - LN	-0,96	0,339
**Eotaxin-3/CCL26 (65)**	U	2	4,24	0,28	0,20	2,54	U - V	1,43	0,151
	V	2	1,64	0,01	0,01	0,13	U - L	-0,96	0,339
	L	2	9,23	1,99	1,41	17,92	U - N	0,96	0,339
	N	2	1,93	0,18	0,13	1,65	U - LN	-1,08	0,282
	LN	2	8,46	0,40	0,28	3,56	L - V	2,39	**0,017**
	C	2	2,69	0,12	0,09	1,08	N - V	0,48	0,633
							LN - V	2,51	**0,012**
							C - U	-0,48	0,633
							C - N	0,48	0,633
							C - LN	-1,55	0,120
**Fractalkine?CX3CL1 (77)**	U	2	16,33	0,54	0,38	4,83	U - V	0,48	0,633
	V	2	10,75	0,91	0,65	8,20	U - L	-0,96	0,339
	L	2	244,93	3,63	2,57	32,65	U - N	0,96	0,339
	N	2	6,31	0,08	0,06	0,76	U - LN	-1,43	0,151
	LN	2	282,00	6,01	4,25	54,00	L - V	1,43	0,151
	C	2	20,52	0,32	0,23	2,86	N - V	-0,48	0,633
							LN - V	1,91	0,056
							C - U	0,48	0,633
							C - N	1,43	0,151
							C - LN	-0,96	0,339
**GCP/CXCL6 (15)**	U	2	3,04	0,16	0,12	1,46	U - V	1,44	0,149
	V	2	1,41	0,00	0,00	0,00	U - L	-0,48	0,631
	L	2	16,16	0,86	0,61	7,69	U - N	0,60	0,548
	N	2	1,74	0,00	0,00	0,00	U - LN	-0,96	0,336
	LN	2	19,78	1,35	0,96	12,13	L - V	1,92	0,054
	C	2	1,70	0,06	0,04	0,51	N - V	0,84	0,400
							LN - V	2,40	**0,016**
							C - U	-0,84	0,400
							C - N	-0,24	0,810
							C - LN	-1,80	0,071
**GMCSF (34)**	U	2	16,33	0,37	0,27	3,37	U - V	1,14	0,256
	V	2	8,27	0,52	0,37	4,70	U - L	-0,48	0,632
	L	2	51,89	5,01	3,55	45,04	U - N	0,48	0,632
	N	2	15,28	0,92	0,65	8,26	U - LN	-0,96	0,338
	LN	2	57,58	1,89	1,34	16,96	L - V	1,62	0,106
	C	2	8,09	0,78	0,56	7,05	N - V	0,66	0,510
							LN - V	2,09	**0,036**
							C - U	-1,26	0,209
							C - N	-0,78	0,437
							C - LN	-2,21	**0,027**
**Gro-a/CXCL1 (61)**	U	2	39,68	2,28	1,62	20,52	U - V	0,48	0,633
	V	2	21,89	0,09	0,07	0,83	U - L	-0,72	0,473
	L	2	76,84	1,83	1,30	16,45	U - N	0,96	0,339
	N	2	17,77	0,19	0,14	1,72	U - LN	-0,72	0,473
	LN	2	79,06	6,10	4,32	54,83	L - V	1,20	0,232
	C	2	15,66	0,40	0,29	3,62	N - V	-0,48	0,633
							LN - V	1,20	0,232
							C - U	-1,43	0,151
							C - N	-0,48	0,633
							C - LN	-2,15	**0,031**
**Gro-b/CXCL2 (78)**	U	2	4,11	0,00	0,00	0,00	U - V	1,08	0,279
	V	2	3,20	0,09	0,07	0,83	U - L	-1,02	0,307
	L	2	6,96	0,96	0,68	8,64	U - N	-0,24	0,810
	N	2	4,34	0,32	0,23	2,86	U - LN	-1,14	0,254
	LN	2	7,20	0,63	0,45	5,65	L - V	2,10	**0,035**
	C	2	3,70	0,08	0,06	0,76	N - V	1,32	0,186
							LN - V	2,22	**0,026**
							C - U	-0,60	0,548
							C - N	-0,84	0,400
							C - LN	-1,74	0,081
**I-309/CCL1 (20)**	U	2	8,85	0,06	0,04	0,51	U - V	1,44	0,151
	V	2	6,16	0,00	0,00	0,00	U - L	-0,48	0,632
	L	2	13,54	0,09	0,07	0,83	U - N	0,72	0,473
	N	2	6,59	0,07	0,05	0,64	U - LN	-0,96	0,338
	LN	2	14,91	0,29	0,21	2,60	L - V	1,91	0,056
	C	2	6,68	0,33	0,23	2,92	N - V	0,72	0,473
							LN - V	2,39	**0,017**
							C - U	-0,72	0,473

							C - N	0,00	1,000
							C - LN	-1,68	0,094
**IFNg (21)**	U	2	0,34	0,05	0,04	0,44	U - V	1,44	0,150
	V	2	0,16	0,01	0,01	0,06	U - L	-0,48	0,631
	L	2	2,03	0,06	0,05	0,57	U - N	0,84	0,401
	N	2	0,21	0,01	0,01	0,06	U - LN	-0,96	0,337
	LN	2	2,43	0,18	0,13	1,59	L - V	1,92	0,055
	C	2	0,21	0,00	0,00	0,00	N - V	0,60	0,548
							LN - V	2,40	**0,016**
							C - U	-0,60	0,548
							C - N	0,24	0,810
							C - LN	-1,56	0,119
**IL1b (39)**	U	2	2,01	0,08	0,06	0,70	U - V	1,44	0,151
	V	2	1,04	0,01	0,01	0,13	U - L	-0,48	0,632
	L	2	73,60	2,12	1,50	19,06	U - N	0,66	0,510
	N	2	1,31	0,11	0,08	0,95	U - LN	-0,96	0,338
	LN	2	76,60	0,71	0,50	6,35	L - V	1,91	0,056
	C	2	1,26	0,04	0,03	0,32	N - V	0,78	0,437
							LN - V	2,39	**0,017**
							C - U	-0,78	0,437
							C - N	-0,12	0,905
							C - LN	-1,73	0,083
**IL2 (38)**	U	2	1,07	0,03	0,02	0,25	U - V	1,44	0,151
	V	2	0,57	0,03	0,02	0,25	U - L	-0,48	0,632
	L	2	3,13	0,19	0,14	1,72	U - N	0,78	0,437
	N	2	0,75	0,06	0,04	0,51	U - LN	-0,96	0,338
	LN	2	4,02	0,31	0,22	2,80	L - V	1,91	0,056
	C	2	0,77	0,03	0,02	0,25	N - V	0,66	0,510
							LN - V	2,39	**0,017**
							C - U	-0,66	0,510
							C - N	0,12	0,905
							C - LN	-1,62	0,106
**IL4 (52)**	U	2	1,72	0,13	0,10	1,21	U - V	1,44	0,151
	V	2	0,62	0,08	0,06	0,76	U - L	-0,54	0,590
	L	2	4,29	0,21	0,15	1,84	U - N	0,54	0,590
	N	2	0,90	0,05	0,04	0,44	U - LN	-0,90	0,369
	LN	2	4,47	0,05	0,04	0,44	L - V	1,98	**0,048**
	C	2	0,79	0,10	0,07	0,89	N - V	0,90	0,369
							LN - V	2,34	**0,019**
							C - U	-0,90	0,369
							C - N	-0,36	0,719
							C - LN	-1,80	0,072
**IL6 (19)**	U	2	3098,91	65,12	46,05	585,12	U - V	1,43	0,151
	V	2	1267,65	61,02	43,15	548,27	U - L	-0,72	0,473
	L	2	11363,23	1104,30	780,86	9921,77	U - N	0,72	0,473
	N	2	2830,89	91,53	64,72	822,35	U - LN	-0,72	0,473
	LN	2	15287,12	7112,38	5029,21	63902,17	L - V	2,15	**0,031**
	C	2	2874,00	12,64	8,94	113,59	N - V	0,72	0,473
							LN - V	2,15	**0,031**
							C - U	-0,72	0,473
							C - N	0,00	1,000
							C - LN	-1,43	0,151
**IL8/CXCL8 (54)**	U	2	6914,06	1178,89	833,60	10591,89	U - V	0,48	0,633
	V	2	1226,25	98,80	69,86	887,66	U - L	-0,72	0,473
	L	2	9611,76	103,28	73,03	927,93	U - N	0,96	0,339
	N	2	629,97	21,76	15,39	195,49	U - LN	-0,72	0,473
	LN	2	10104,84	932,72	659,54	8380,19	L - V	1,20	0,232
	C	2	535,77	22,08	15,61	198,34	N - V	-0,48	0,633
							LN - V	1,20	0,232
							C - U	-1,43	0,151
							C - N	-0,48	0,633
							C - LN	-2,15	**0,031**
**IL10 (56)**	U	2	15,25	0,21	0,15	1,91	U - V	0,48	0,632
	V	2	7,78	0,00	0,00	0,00	U - L	-0,96	0,338
	L	2	27,15	0,42	0,30	3,81	U - N	1,08	0,282
	N	2	5,65	0,37	0,27	3,37	U - LN	-0,48	0,632
	LN	2	25,18	1,68	1,19	15,12	L - V	1,44	0,151
	C	2	5,36	0,16	0,11	1,40	N - V	-0,60	0,550
							LN - V	0,96	0,338
							C - U	-1,32	0,188
							C - N	-0,24	0,811
							C - LN	-1,79	0,073
**IL16 (27)**	U	2	13,62	0,27	0,19	2,41	U - V	1,43	0,151
	V	2	5,55	0,12	0,09	1,08	U - L	-0,48	0,633
	L	2	51,10	0,87	0,62	7,81	U - N	0,96	0,339
	N	2	6,32	0,24	0,17	2,16	U - LN	-0,96	0,339
	LN	2	52,94	0,87	0,62	7,81	L - V	1,91	0,056
	C	2	7,11	0,12	0,09	1,08	N - V	0,48	0,633
							LN - V	2,39	**0,017**
							C - U	-0,48	0,633
							C - N	0,48	0,633

							C - LN	-1,43	0,151
**IP10/CXCL10 (48)**	U	2	1,76	0,02	0,02	0,19	U - V	1,20	0,232
	V	2	1,06	0,04	0,03	0,32	U - L	-0,48	0,633
	L	2	3,56	0,13	0,10	1,21	U - N	1,20	0,232
	N	2	1,02	0,14	0,10	1,27	U - LN	-0,96	0,339
	LN	2	4,08	0,02	0,02	0,19	L - V	1,67	0,094
	C	2	1,31	0,06	0,05	0,57	N - V	0,00	1,000
							LN - V	2,15	**0,031**
							C - U	-0,48	0,633
							C - N	0,72	0,473
							C - LN	-1,43	0,151
**I-TAC/CXCL11 (25)**	U	2	2,82	0,00	0,00	0,00	U - V	0,96	0,338
	V	2	1,75	0,04	0,03	0,32	U - L	-1,44	0,151
	L	2	43,59	7,09	5,01	63,66	U - N	1,44	0,151
	N	2	1,36	0,06	0,04	0,51	U - LN	-0,48	0,632
	LN	2	7,09	0,43	0,31	3,88	L - V	2,39	**0,017**
	C	2	2,41	0,06	0,04	0,51	N - V	-0,48	0,632
							LN - V	1,44	0,151
							C - U	-0,48	0,632
							C - N	0,96	0,338
							C - LN	-0,96	0,338
**MCP-1/CCL2 (53)**	U	2	384,49	8,65	6,12	77,76	U - V	0,96	0,339
	V	2	166,97	4,24	3,00	38,12	U - L	-1,43	0,151
	L	2	1820,43	1,95	1,38	17,53	U - N	1,43	0,151
	N	2	55,56	1,55	1,10	13,91	U - LN	-0,48	0,633
	LN	2	825,41	88,76	62,76	797,44	L - V	2,39	**0,017**
	C	2	263,46	7,84	5,55	70,46	N - V	-0,48	0,633
							LN - V	1,43	0,151
							C - U	-0,48	0,633
							C - N	0,96	0,339
							C - LN	-0,96	0,339
**MCP-2/CCL8 (57)**	U	2	0,38	0,01	0,01	0,13	U - V	0,66	0,510
	V	2	0,24	0,02	0,02	0,19	U - L	-0,96	0,338
	L	2	0,74	0,01	0,01	0,06	U - N	0,96	0,338
	N	2	0,22	0,01	0,01	0,13	U - LN	-0,48	0,632
	LN	2	0,69	0,01	0,01	0,06	L - V	1,62	0,106
	C	2	0,21	0,01	0,01	0,13	N - V	-0,30	0,765
							LN - V	1,14	0,256
							C - U	-1,26	0,209
							C - N	-0,30	0,765
							C - LN	-1,73	0,083
**MCP-3/CCL7 (26)**	U	2	7,74	0,24	0,17	2,16	U - V	1,44	0,151
	V	2	3,52	0,11	0,08	1,02	U - L	-0,48	0,632
	L	2	131,66	3,82	2,70	34,31	U - N	0,78	0,437
	N	2	4,43	0,23	0,17	2,10	U - LN	-0,96	0,338
	LN	2	139,80	4,99	3,53	44,85	L - V	1,91	0,056
	C	2	4,59	0,47	0,33	4,19	N - V	0,66	0,510
							LN - V	2,39	**0,017**
							C - U	-0,66	0,510
							C - N	0,12	0,905
							C - LN	-1,62	0,106
**MCP-4/CCL13 (28)**	U	2	11,86	0,74	0,53	6,67	U - V	0,48	0,633
	V	2	4,66	0,19	0,14	1,72	U - L	-0,96	0,339
	L	2	22,22	0,22	0,16	1,97	U - N	0,96	0,339
	N	2	3,18	0,11	0,08	0,95	U - LN	-0,48	0,633
	LN	2	18,93	3,08	2,18	27,70	L - V	1,43	0,151
	C	2	2,45	0,01	0,01	0,13	N - V	-0,48	0,633
							LN - V	0,96	0,339
							C - U	-1,43	0,151
							C - N	-0,48	0,633
							C - LN	-1,91	0,056
**MDC/CCL22 (29)**	U	2	2,84	0,12	0,09	1,08	U - V	1,02	0,309
	V	2	2,36	0,11	0,08	0,95	U - L	-0,72	0,472
	L	2	3,59	0,07	0,05	0,64	U - N	0,66	0,510
	N	2	2,43	0,06	0,05	0,57	U - LN	-0,72	0,472
	LN	2	3,66	0,37	0,27	3,37	L - V	1,74	0,082
	C	2	2,33	0,07	0,05	0,64	N - V	0,36	0,719
							LN - V	1,74	0,082
							C - U	-1,20	0,231
							C - N	-0,54	0,590
							C - LN	-1,92	0,055
**MIF (35)**	U	2	437,57	53,88	38,10	484,11	U - V	0,48	0,633
	V	2	326,56	11,20	7,92	100,63	U - L	-0,96	0,339
	L	2	597,92	38,57	27,27	346,50	U - N	-1,91	0,056
	N	2	1236,77	68,04	48,12	611,36	U - LN	-2,39	**0,017**
	LN	2	1754,37	14,30	10,12	128,52	L - V	1,43	0,151
	C	2	529,05	9,84	6,96	88,44	N - V	2,39	**0,017**
							LN - V	2,87	**0,004**
							C - U	0,48	0,633
							C - N	-1,43	0,151
							C - LN	-1,91	0,056

**MIG/CXCL9 (14)**	U	2	9,31	0,62	0,44	5,59	U - V	1,43	0,151
	V	2	6,17	0,20	0,14	1,78	U - L	-0,48	0,633
	L	2	21,52	0,56	0,40	5,02	U - N	0,48	0,633
	N	2	7,53	0,13	0,10	1,21	U - LN	-0,96	0,339
	LN	2	23,49	0,62	0,44	5,53	L - V	1,91	0,056
	C	2	6,97	0,13	0,09	1,14	N - V	0,96	0,339
							LN - V	2,39	**0,017**
							C - U	-0,96	0,339
							C - N	-0,48	0,633
							C - LN	-1,91	0,056
**MIP-1a/CCL3 (55)**	U	2	1,04	0,02	0,02	0,19	U - V	0,78	0,437
	V	2	0,66	0,03	0,02	0,25	U - L	-0,48	0,632
	L	2	1,51	0,01	0,01	0,13	U - N	1,08	0,282
	N	2	0,64	0,04	0,03	0,38	U - LN	-0,96	0,338
	LN	2	1,81	0,03	0,02	0,25	L - V	1,26	0,209
	C	2	0,65	0,01	0,01	0,13	N - V	-0,30	0,765
							LN - V	1,73	0,083
							C - U	-1,02	0,309
							C - N	0,06	0,952
							C - LN	-1,97	**0,048**
**MIP1b/CCL15 (66)**	U	2	4,24	0,31	0,22	2,80	U - V	0,96	0,337
	V	2	2,33	0,13	0,10	1,21	U - L	-1,20	0,230
	L	2	10,81	0,23	0,16	2,03	U - N	0,84	0,401
	N	2	2,31	0,05	0,04	0,44	U - LN	-0,48	0,631
	LN	2	6,99	0,04	0,03	0,38	L - V	2,16	**0,031**
	C	2	2,27	0,00	0,00	0,00	N - V	0,12	0,904
							LN - V	1,44	0,150
							C - U	-1,08	0,280
							C - N	-0,24	0,810
							C - LN	-1,56	0,119
**MIP-3a/CCL20 (62)**	U	2	0,73	0,00	0,00	0,00	U - V	1,44	0,151
	V	2	0,39	0,01	0,01	0,06	U - L	-0,48	0,632
	L	2	2,30	0,11	0,08	1,02	U - N	0,96	0,338
	N	2	0,45	0,03	0,02	0,25	U - LN	-0,96	0,338
	LN	2	13,78	1,09	0,77	9,78	L - V	1,91	0,056
	C	2	0,70	0,01	0,01	0,13	N - V	0,48	0,632
							LN - V	2,39	**0,017**
							C - U	-0,48	0,632
							C - N	0,48	0,632
							C - LN	-1,44	0,151
**MIP3-b/CCL19 (76)**	U	2	5,89	0,38	0,27	3,43	U - V	0,84	0,401
	V	2	2,59	0,00	0,00	0,00	U - L	-0,84	0,401
	L	2	35,11	1,50	1,06	13,47	U - N	1,44	0,150
	N	2	2,31	0,06	0,04	0,51	U - LN	-0,84	0,401
	LN	2	35,40	3,61	2,56	32,46	L - V	1,68	0,093
	C	2	2,74	0,21	0,15	1,84	N - V	-0,60	0,548
							LN - V	1,68	0,093
							C - U	-0,60	0,548
							C - N	0,84	0,401
							C - LN	-1,44	0,150
**MPIF-1/CCL23 (37)**	U	2	3,13	0,08	0,06	0,76	U - V	0,66	0,510
	V	2	1,92	0,08	0,06	0,76	U - L	-0,60	0,550
	L	2	8,63	0,40	0,29	3,62	U - N	0,78	0,437
	N	2	1,89	0,13	0,10	1,21	U - LN	-0,84	0,402
	LN	2	9,07	0,54	0,39	4,89	L - V	1,26	0,209
	C	2	1,53	0,10	0,07	0,89	N - V	-0,12	0,905
							LN - V	1,50	0,135
							C - U	-1,44	0,151
							C - N	-0,66	0,510
							C - LN	-2,27	**0,023**
**SCYB16/CXCL16 (64)**	U	2	5,77	0,27	0,19	2,41	U - V	0,96	0,338
	V	2	3,65	0,04	0,03	0,38	U - L	-1,44	0,151
	L	2	22,65	1,75	1,24	15,76	U - N	1,44	0,151
	N	2	1,24	0,00	0,00	0,00	U - LN	0,48	0,632
	LN	2	4,73	0,09	0,07	0,83	L - V	2,39	**0,017**
	C	2	13,99	0,44	0,31	3,94	N - V	-0,48	0,632
							LN - V	0,48	0,632
							C - U	0,96	0,338
							C - N	2,39	**0,017**
							C - LN	1,44	0,151
**SDF-1ab/CXCL12 (22)**	U	2	84,67	0,18	0,13	1,65	U - V	0,48	0,633
	V	2	57,04	0,86	0,61	7,75	U - L	-1,43	0,151
	L	2	296,78	9,48	6,70	85,13	U - N	1,43	0,151
	N	2	34,22	0,45	0,32	4,00	U - LN	-0,60	0,550
	LN	2	114,97	3,44	2,43	30,88	L - V	1,91	0,056
	C	2	49,25	0,24	0,17	2,16	N - V	-0,96	0,339
							LN - V	1,08	0,282
							C - U	-0,96	0,339
							C - N	0,48	0,633
							C - LN	-1,55	0,120
**TARC/CCL17 (67)**	U	2	5,24	0,23	0,17	2,10	U - V	1,43	0,151

	V	2	3,02	0,04	0,03	0,38	U - L	-0,48	0,633
	L	2	17,38	1,44	1,02	12,96	U - N	0,48	0,633
	N	2	3,77	0,04	0,03	0,38	U - LN	-0,96	0,339
	LN	2	19,61	1,39	0,99	12,52	L - V	1,91	0,056
	C	2	3,49	0,08	0,06	0,76	N - V	0,96	0,339
							LN - V	2,39	**0,017**
							C - U	-0,96	0,339
							C - N	-0,48	0,633
							C - LN	-1,91	0,056
**TERC/CCL25 (46)**	U	2	49,51	0,30	0,22	2,73	U - V	1,43	0,151
	V	2	22,63	1,75	1,24	15,69	U - L	-0,48	0,633
	L	2	82,09	2,46	1,74	22,11	U - N	0,72	0,473
	N	2	29,67	1,67	1,18	14,99	U - LN	-0,96	0,339
	LN	2	109,38	11,02	7,79	98,98	L - V	1,91	0,056
	C	2	29,57	0,42	0,30	3,75	N - V	0,72	0,473
							LN - V	2,39	**0,017**
							C - U	-0,72	0,473
							C - N	0,00	1,000
							C - LN	-1,67	0,094
**TNFa (36)**	U	2	2,63	0,09	0,07	0,83	U - V	1,20	0,231
	V	2	1,34	0,00	0,00	0,00	U - L	-0,48	0,632
	L	2	4,99	0,34	0,24	3,05	U - N	0,48	0,632
	N	2	1,72	0,04	0,03	0,32	U - LN	-0,96	0,338
	LN	2	11,34	0,47	0,33	4,19	L - V	1,68	0,094
	C	2	1,43	0,16	0,11	1,40	N - V	0,72	0,473
							LN - V	2,15	**0,031**
							C - U	-1,20	0,231
							C - N	-0,72	0,473
							C - LN	-2,15	**0,031**

SD: Standart deviation**, SE:** Standard Error**, CI:** Confidence Interval**, U:** Untreated**, V:** VX765**, L:** LPS**, N:** Nigericin**, LN:** LPS&Nigericin**, C:** Champtothesin

P value calculated using Kruskal-Wallis multiple comparison test

Supplemantary Table 4F: Cytokine secretion levels in fibroblasts after VX765, LPS, Nigericin and LPS&Nigericin treatments

	Type	N	Mean	SD	SE	CI 95 %	**Kruskal-Wallis multiple comparison**	Z	P
**6Cine/CCL21 (12)**	U	2	4,95	0,07	0,05	0,64	U - V	-1,91	0,056
	V	2	33,87	0,69	0,49	6,23	U - L	-2,39	**0,017**
	L	2	65,17	2,03	1,44	18,23	U - N	-1,32	0,188
	N	2	20,35	6,96	4,92	62,51	U - LN	-1,08	0,282
	LN	2	15,05	4,59	3,25	41,23	L - V	0,48	0,632
	C	2	4,06	0,04	0,03	0,38	N - V	-0,60	0,550
							LN - V	-0,84	0,402
							C - U	-0,48	0,632
							C - N	-1,79	0,073
							C - LN	-1,56	0,120
**BCA-1/CXCL13 (74)**	U	2	0,39	0,00	0,00	0,00	U - V	-1,80	0,071
	V	2	0,70	0,02	0,02	0,19	U - L	-2,28	**0,022**
	L	2	1,32	0,07	0,05	0,64	U - N	-1,32	0,186
	N	2	0,54	0,06	0,05	0,57	U - LN	-0,30	0,764
	LN	2	0,41	0,03	0,02	0,25	L - V	0,48	0,631
	C	2	0,06	0,01	0,01	0,13	N - V	-0,48	0,631
							LN - V	-1,50	0,133
							C - U	-0,60	0,548
							C - N	-1,92	0,054
							C - LN	-0,90	0,367
**CTACK/CCL27 (72)**	U	2	0,50	0,00	0,00	0,00	U - V	-1,92	0,055
	V	2	1,37	0,04	0,03	0,32	U - L	-2,40	**0,017**
	L	2	2,82	0,07	0,05	0,64	U - N	-1,26	0,208
	N	2	0,85	0,14	0,10	1,27	U - LN	-0,84	0,402
	LN	2	0,75	0,14	0,10	1,27	L - V	0,48	0,632
	C	2	0,49	0,01	0,01	0,06	N - V	-0,66	0,510
							LN - V	-1,08	0,281
							C - U	-0,48	0,632
							C - N	-1,74	0,082
							C - LN	-1,32	0,188
**ENA-78/CXCL5 (73)**	U	2	74,73	5,88	4,16	52,86	U - V	-2,03	**0,042**
	V	2	714,08	122,15	86,38	1097,50	U - L	-2,27	**0,023**
	L	2	775,26	45,18	31,95	405,96	U - N	-1,43	0,151
	N	2	388,19	210,02	148,51	1886,93	U - LN	-0,96	0,339
	LN	2	211,70	1,31	0,93	11,75	L - V	0,24	0,811
	C	2	65,34	0,18	0,13	1,59	N - V	-0,60	0,550
							LN - V	-1,08	0,282
							C - U	-0,48	0,633
							C - N	-1,91	0,056
							C - LN	-1,43	0,151

**Eotaxin/CCL11 (43)**	U	2	2,84	0,32	0,23	2,86	U - V	-1,91	0,056
	V	2	9,68	0,06	0,04	0,51	U - L	-2,39	**0,017**
	L	2	19,72	0,14	0,10	1,27	U - N	-1,43	0,151
	N	2	7,84	1,73	1,22	15,50	U - LN	-0,60	0,550
	LN	2	5,02	0,41	0,29	3,68	L - V	0,48	0,633
	C	2	2,13	0,11	0,08	1,02	N - V	-0,48	0,633
							LN - V	-1,31	0,189
							C - U	-0,48	0,633
							C - N	-1,91	0,056
							C - LN	-1,08	0,282
**Eotaxin2/CCL24 (30)**	U	2	0,42	0,03	0,02	0,25	U - V	-1,68	0,094
	V	2	2,56	0,00	0,00	0,00	U - L	-2,39	**0,017**
	L	2	3,38	0,21	0,15	1,84	U - N	-1,56	0,120
	N	2	2,33	0,69	0,49	6,23	U - LN	-0,84	0,402
	LN	2	1,72	0,29	0,21	2,60	L - V	0,72	0,473
	C	2	0,22	0,01	0,01	0,13	N - V	-0,12	0,905
							LN - V	-0,84	0,402
							C - U	-0,48	0,632
							C - N	-2,03	**0,042**
							C - LN	-1,32	0,188
**Eotaxin-3/CCL26 (65)**	U	2	0,95	0,00	0,00	0,00	U - V	-1,91	0,056
	V	2	2,83	0,04	0,03	0,38	U - L	-2,39	**0,017**
	L	2	5,93	0,04	0,03	0,32	U - N	-1,32	0,188
	N	2	1,77	0,41	0,29	3,68	U - LN	-0,84	0,402
	LN	2	1,37	0,33	0,24	2,99	L - V	0,48	0,632
	C	2	0,36	0,30	0,22	2,73	N - V	-0,60	0,550
							LN - V	-1,08	0,282
							C - U	-0,48	0,632
							C - N	-1,79	0,073
							C - LN	-1,32	0,188
**Fractalkine?CX3CL1 (77)**	U	2	61,95	0,40	0,28	3,56	U - V	1,79	0,073
	V	2	50,15	0,87	0,62	7,81	U - L	-0,48	0,633
	L	2	374,58	10,02	7,09	90,02	U - N	1,43	0,151
	N	2	51,31	0,80	0,57	7,18	U - LN	0,84	0,403
	LN	2	53,10	2,22	1,57	19,95	L - V	2,27	**0,023**
	C	2	41,08	0,17	0,12	1,52	N - V	0,36	0,720
							LN - V	0,96	0,339
							C - U	-2,39	**0,017**
							C - N	-0,96	0,339
							C - LN	-1,55	0,120
**GCP/CXCL6 (15)**	U	2	61,27	2,71	1,92	24,33	U - V	-1,20	0,232
	V	2	519,26	34,63	24,49	311,11	U - L	-1,20	0,232
	L	2	523,07	44,68	31,60	401,45	U - N	-0,48	0,633
	N	2	107,58	40,22	28,44	361,36	U - LN	0,60	0,550
	LN	2	27,51	1,81	1,28	16,26	L - V	0,00	1,000
	C	2	2,78	0,12	0,09	1,08	N - V	-0,72	0,473
							LN - V	-1,79	0,073
							C - U	-1,43	0,151
							C - N	-1,91	0,056
							C - LN	-0,84	0,403
**GMCSF (34)**	U	2	3,66	0,07	0,05	0,64	U - V	-1,32	0,188
	V	2	19,01	3,86	2,73	34,69	U - L	-2,39	**0,017**
	L	2	39,80	6,14	4,35	55,21	U - N	-1,56	0,120
	N	2	22,74	7,65	5,41	68,74	U - LN	-0,90	0,370
	LN	2	11,75	10,33	7,31	92,82	L - V	1,08	0,282
	C	2	1,52	0,21	0,15	1,84	N - V	0,24	0,811
							LN - V	-0,42	0,675
							C - U	-0,48	0,632
							C - N	-2,03	**0,042**
							C - LN	-1,38	0,169
**Gro-a/CXCL1 (61)**	U	2	106,08	2,99	2,12	26,87	U - V	-2,63	**0,009**
	V	2	1909,18	318,42	225,16	2860,87	U - L	-2,15	**0,031**
	L	2	1529,69	157,00	111,02	1410,58	U - N	-1,43	0,151
	N	2	482,61	336,71	238,09	3025,22	U - LN	-1,43	0,151
	LN	2	323,74	23,39	16,54	210,16	L - V	-0,48	0,633
	C	2	104,66	0,08	0,06	0,70	N - V	-1,20	0,232
							LN - V	-1,20	0,232
							C - U	0,00	1,000
							C - N	-1,43	0,151
							C - LN	-1,43	0,151
**Gro-b/CXCL2 (78)**	U	2	0,82	0,17	0,12	1,52	U - V	-0,60	0,550
	V	2	11,08	1,07	0,76	9,59	U - L	-2,15	**0,031**
	L	2	39,88	4,26	3,02	38,31	U - N	-2,39	**0,017**
	N	2	41,30	4,67	3,31	41,99	U - LN	-1,08	0,282
	LN	2	14,54	0,21	0,15	1,84	L - V	1,55	0,120
	C	2	0,61	0,24	0,17	2,16	N - V	1,79	0,073
							LN - V	0,48	0,633
							C - U	-0,24	0,811
							C - N	-2,63	**0,009**
							C - LN	-1,31	0,189
**I-309/CCL1 (20)**	U	2	3,84	0,00	0,00	0,00	U - V	-1,92	0,055

	V	2	7,92	0,00	0,00	0,00	U - L	-2,40	**0,017**
	L	2	15,05	1,31	0,93	11,75	U - N	-1,44	0,151
	N	2	6,49	1,05	0,75	9,47	U - LN	-0,96	0,338
	LN	2	5,20	0,40	0,29	3,62	L - V	0,48	0,632
	C	2	3,20	0,01	0,01	0,13	N - V	-0,48	0,632
							LN - V	-0,96	0,338
							C - U	-0,48	0,632
							C - N	-1,92	0,055
							C - LN	-1,44	0,151
**IFNg (21)**	U	2	0,25	0,01	0,01	0,06	U - V	-1,20	0,231
	V	2	1,14	0,13	0,10	1,21	U - L	-1,68	0,094
	L	2	2,57	0,14	0,10	1,27	U - N	-0,60	0,549
	N	2	0,45	0,16	0,11	1,40	U - LN	0,12	0,905
	LN	2	0,31	0,20	0,14	1,78	L - V	0,48	0,632
	C	2	0,10	0,00	0,00	0,00	N - V	-0,60	0,549
							LN - V	-1,32	0,188
							C - U	-1,20	0,231
							C - N	-1,80	0,072
							C - LN	-1,08	0,281
**IL1b (39)**	U	2	64,75	0,81	0,57	7,24	U - V	0,84	0,403
	V	2	55,79	2,23	1,58	20,01	U - L	-1,43	0,151
	L	2	88,08	0,07	0,05	0,64	U - N	0,60	0,550
	N	2	60,49	4,98	3,52	44,73	U - LN	-0,48	0,633
	LN	2	75,60	0,60	0,43	5,40	L - V	2,27	**0,023**
	C	2	4,72	0,42	0,30	3,75	N - V	0,24	0,811
							LN - V	1,31	0,189
							C - U	-1,43	0,151
							C - N	-0,84	0,403
							C - LN	-1,91	0,056
**IL2 (38)**	U	2	0,09	0,02	0,02	0,19	U - V	-1,74	0,082
	V	2	1,11	0,31	0,22	2,80	U - L	-2,22	**0,027**
	L	2	5,21	1,13	0,80	10,16	U - N	-1,20	0,230
	N	2	0,27	0,21	0,15	1,84	U - LN	0,00	1,000
	LN	2	0,09	0,01	0,01	0,06	L - V	0,48	0,631
	C	2	0,07	0,01	0,01	0,13	N - V	-0,54	0,589
							LN - V	-1,74	0,082
							C - U	-0,48	0,631
							C - N	-1,68	0,093
							C - LN	-0,48	0,631
**IL4 (52)**	U	2	0,91	0,00	0,00	0,00	U - V	-1,74	0,082
	V	2	2,60	0,14	0,10	1,27	U - L	-2,40	**0,017**
	L	2	4,15	0,21	0,15	1,84	U - N	-1,50	0,134
	N	2	2,37	0,47	0,33	4,19	U - LN	-0,96	0,338
	LN	2	2,01	0,21	0,15	1,91	L - V	0,66	0,510
	C	2	0,81	0,04	0,03	0,38	N - V	-0,24	0,811
							LN - V	-0,78	0,436
							C - U	-0,48	0,632
							C - N	-1,98	**0,048**
							C - LN	-1,44	0,151
**IL6 (19)**	U	2	25,34	0,31	0,22	2,80	U - V	-1,67	0,094
	V	2	693,62	14,53	10,28	130,56	U - L	-2,39	**0,017**
	L	2	3594,15	116,76	82,57	1049,09	U - N	-1,67	0,094
	N	2	729,15	124,30	87,89	1116,75	U - LN	-0,72	0,473
	LN	2	345,78	64,97	45,94	583,72	L - V	0,72	0,473
	C	2	23,96	1,09	0,77	9,78	N - V	0,00	1,000
							LN - V	-0,96	0,339
							C - U	-0,48	0,633
							C - N	-2,15	**0,031**
							C - LN	-1,20	0,232
**IL8/CXCL8 (54)**	U	2	138,48	9,55	6,76	85,83	U - V	-1,91	0,056
	V	2	3928,80	750,65	530,79	6744,33	U - L	-2,39	**0,017**
	L	2	7854,08	267,83	189,39	2406,36	U - N	-1,43	0,151
	N	2	1780,53	852,68	602,94	7661,02	U - LN	-0,96	0,339
	LN	2	635,01	28,73	20,32	258,13	L - V	0,48	0,633
	C	2	105,11	4,27	3,02	38,37	N - V	-0,48	0,633
							LN - V	-0,96	0,339
							C - U	-0,48	0,633
							C - N	-1,91	0,056
							C - LN	-1,43	0,151
**IL10 (56)**	U	2	5,20	0,66	0,47	5,97	U - V	-1,79	0,073
	V	2	20,16	1,12	0,79	10,04	U - L	-2,39	**0,017**
	L	2	25,21	2,50	1,77	22,43	U - N	-1,43	0,151
	N	2	15,19	6,51	4,60	58,45	U - LN	-1,08	0,282
	LN	2	9,46	2,68	1,90	24,08	L - V	0,60	0,550
	C	2	2,30	0,28	0,20	2,54	N - V	-0,36	0,720
							LN - V	-0,72	0,473
							C - U	-0,48	0,633
							C - N	-1,91	0,056
							C - LN	-1,55	0,120
**IL16 (27)**	U	2	5,15	2,93	2,08	26,37	U - V	-1,56	0,120
	V	2	21,99	0,46	0,33	4,13	U - L	-2,03	**0,042**

	L	2	61,49	6,89	4,88	61,94	U - N	-0,12	0,905
	N	2	6,90	6,14	4,34	55,14	U - LN	-0,24	0,811
	LN	2	7,19	3,85	2,72	34,56	L - V	0,48	0,632
	C	2	5,63	0,21	0,15	1,84	N - V	-1,44	0,151
							LN - V	-1,32	0,188
							C - U	-0,12	0,905
							C - N	-0,24	0,811
							C - LN	-0,36	0,720
**IP10/CXCL10 (48)**	U	2	0,67	0,01	0,01	0,13	U - V	-1,92	0,055
	V	2	8,70	0,11	0,08	0,95	U - L	-2,40	**0,017**
	L	2	23,50	2,75	1,95	24,71	U - N	-1,44	0,151
	N	2	2,61	0,47	0,34	4,26	U - LN	-0,78	0,436
	LN	2	0,88	0,03	0,02	0,25	L - V	0,48	0,632
	C	2	0,49	0,00	0,00	0,00	N - V	-0,48	0,632
							LN - V	-1,14	0,255
							C - U	-0,48	0,632
							C - N	-1,92	0,055
							C - LN	-1,26	0,208
**I-TAC/CXCL11 (25)**	U	2	1,21	0,00	0,00	0,00	U - V	-1,69	0,091
	V	2	5,40	0,10	0,07	0,89	U - L	-2,18	**0,030**
	L	2	11,99	1,31	0,93	11,75	U - N	-1,21	0,227
	N	2	3,87	0,91	0,64	8,13	U - LN	-0,42	0,672
	LN	2	1,38	0,24	0,17	2,16	L - V	0,48	0,629
	C	2	0,52	0,49	0,35	4,45	N - V	-0,48	0,629
							LN - V	-1,27	0,204
							C - U	-0,73	0,468
							C - N	-1,93	0,053
							C - LN	-1,15	0,251
**MCP-1/CCL2 (53)**	U	2	10,42	2,03	1,44	18,23	U - V	-1,91	0,056
	V	2	273,70	1,89	1,34	16,96	U - L	-2,39	**0,017**
	L	2	898,66	16,60	11,74	149,11	U - N	-0,48	0,633
	N	2	40,90	2,94	2,08	26,43	U - LN	-0,96	0,339
	LN	2	72,08	6,46	4,57	58,07	L - V	0,48	0,633
	C	2	6,17	1,37	0,97	12,33	N - V	-1,43	0,151
							LN - V	-0,96	0,339
							C - U	-0,48	0,633
							C - N	-0,96	0,339
							C - LN	-1,43	0,151
**MCP-2/CCL8 (57)**	U	2	0,26	0,06	0,05	0,57	U - V	-1,56	0,120
	V	2	4,28	0,12	0,09	1,08	U - L	-2,03	**0,042**
	L	2	4,70	0,16	0,12	1,46	U - N	-1,08	0,282
	N	2	1,36	0,21	0,15	1,91	U - LN	0,36	0,720
	LN	2	0,22	0,04	0,03	0,32	L - V	0,48	0,632
	C	2	0,26	0,08	0,06	0,70	N - V	-0,48	0,632
							LN - V	-1,91	0,056
							C - U	0,00	1,000
							C - N	-1,08	0,282
							C - LN	0,36	0,720
**MCP-3/CCL7 (26)**	U	2	40,80	0,58	0,41	5,21	U - V	-1,68	0,093
	V	2	115,98	5,22	3,69	46,89	U - L	-2,16	**0,031**
	L	2	213,75	0,37	0,27	3,37	U - N	-1,08	0,280
	N	2	66,46	7,09	5,01	63,66	U - LN	-0,24	0,810
	LN	2	50,25	15,85	11,21	142,37	L - V	0,48	0,631
	C	2	5,95	1,36	0,96	12,20	N - V	-0,60	0,548
							LN - V	-1,44	0,150
							C - U	-0,72	0,471
							C - N	-1,80	0,072
							C - LN	-0,96	0,337
**MCP-4/CCL13 (28)**	U	2	2,29	0,02	0,02	0,19	U - V	-2,39	**0,017**
	V	2	27,26	0,08	0,06	0,70	U - L	-1,91	0,056
	L	2	23,77	3,92	2,78	35,26	U - N	-1,43	0,151
	N	2	12,01	6,99	4,94	62,77	U - LN	-0,96	0,339
	LN	2	5,77	0,30	0,21	2,67	L - V	-0,48	0,633
	C	2	0,50	0,28	0,20	2,54	N - V	-0,96	0,339
							LN - V	-1,43	0,151
							C - U	-0,48	0,633
							C - N	-1,91	0,056
							C - LN	-1,43	0,151
**MDC/CCL22 (29)**	U	2	1,20	0,12	0,09	1,08	U - V	-1,91	0,056
	V	2	2,53	0,04	0,03	0,32	U - L	-2,39	**0,017**
	L	2	3,52	0,04	0,03	0,32	U - N	-1,44	0,151
	N	2	2,14	0,30	0,21	2,67	U - LN	-0,78	0,437
	LN	2	1,78	0,08	0,06	0,70	L - V	0,48	0,632
	C	2	1,09	0,01	0,01	0,06	N - V	-0,48	0,632
							LN - V	-1,14	0,256
							C - U	-0,48	0,632
							C - N	-1,91	0,056
							C - LN	-1,26	0,209
**MIF (35)**	U	2	10,16	0,08	0,06	0,70	U - V	-0,48	0,633
	V	2	164,33	0,27	0,19	2,41	U - L	-0,96	0,339
	L	2	203,21	5,04	3,57	45,30	U - N	-1,43	0,151

	N	2	355,98	107,87	76,28	969,17	U - LN	-2,15	**0,031**
	LN	2	631,51	35,60	25,17	319,82	L - V	0,48	0,633
	C	2	7,62	0,15	0,11	1,33	N - V	0,96	0,339
							LN - V	1,67	0,094
							C - U	-0,48	0,633
							C - N	-1,91	0,056
							C - LN	-2,63	**0,009**
**MIG/CXCL9 (14)**	U	2	7,56	0,77	0,55	6,92	U - V	-2,16	**0,031**
	V	2	14,42	0,24	0,17	2,16	U - L	-2,64	**0,008**
	L	2	21,97	0,83	0,59	7,43	U - N	-1,68	0,094
	N	2	11,67	0,56	0,40	5,02	U - LN	-1,14	0,255
	LN	2	9,54	0,65	0,46	5,84	L - V	0,48	0,632
	C	2	7,98	0,00	0,00	0,00	N - V	-0,48	0,632
							LN - V	-1,02	0,309
							C - U	0,00	1,000
							C - N	-1,68	0,094
							C - LN	-1,14	0,255
**MIP-1a/CCL3 (55)**	U	2	0,30	0,01	0,01	0,13	U - V	-1,38	0,167
	V	2	0,46	0,03	0,02	0,25	U - L	-1,86	0,062
	L	2	2,47	0,22	0,16	1,97	U - N	-0,66	0,509
	N	2	0,32	0,01	0,01	0,13	U - LN	-0,54	0,589
	LN	2	0,32	0,01	0,01	0,06	L - V	0,48	0,631
	C	2	0,29	0,01	0,01	0,06	N - V	-0,72	0,471
							LN - V	-0,84	0,400
							C - U	-0,48	0,631
							C - N	-1,14	0,254
							C - LN	-1,02	0,307
**MIP1b/CCL15 (66)**	U	2	1,59	0,00	0,00	0,00	U - V	-1,92	0,055
	V	2	5,60	0,04	0,03	0,38	U - L	-2,40	**0,017**
	L	2	8,34	0,47	0,33	4,19	U - N	-1,44	0,151
	N	2	3,23	0,13	0,09	1,14	U - LN	-0,90	0,369
	LN	2	2,52	0,16	0,12	1,46	L - V	0,48	0,632
	C	2	1,18	0,01	0,01	0,06	N - V	-0,48	0,632
							LN - V	-1,02	0,309
							C - U	-0,48	0,632
							C - N	-1,92	0,055
							C - LN	-1,38	0,168
**MIP-3a/CCL20 (62)**	U	2	0,32	0,00	0,00	0,00	U - V	-0,96	0,338
	V	2	1,03	0,04	0,03	0,38	U - L	-2,39	**0,017**
	L	2	54,61	4,62	3,27	41,55	U - N	-0,48	0,632
	N	2	0,79	0,13	0,09	1,14	U - LN	-1,79	0,073
	LN	2	1,24	0,07	0,05	0,64	L - V	1,44	0,151
	C	2	0,15	0,04	0,03	0,38	N - V	-0,48	0,632
							LN - V	0,84	0,402
							C - U	-0,48	0,632
							C - N	-0,96	0,338
							C - LN	-2,27	**0,023**
**MIP3-b/CCL19 (76)**	U	2	5,99	0,73	0,52	6,54	U - V	-1,91	0,056
	V	2	24,49	0,28	0,20	2,54	U - L	-2,39	**0,017**
	L	2	31,33	4,90	3,47	44,03	U - N	-1,31	0,189
	N	2	11,62	2,44	1,73	21,92	U - LN	-1,08	0,282
	LN	2	10,02	1,00	0,71	8,96	L - V	0,48	0,633
	C	2	5,25	0,01	0,01	0,13	N - V	-0,60	0,550
							LN - V	-0,84	0,403
							C - U	-0,48	0,633
							C - N	-1,79	0,073
							C - LN	-1,55	0,120
**MPIF-1/CCL23 (37)**	U	2	0,59	0,02	0,02	0,19	U - V	-1,92	0,055
	V	2	5,74	0,00	0,00	0,00	U - L	-2,40	**0,017**
	L	2	10,23	0,50	0,36	4,51	U - N	-1,44	0,151
	N	2	3,86	0,83	0,59	7,50	U - LN	-0,72	0,472
	LN	2	2,21	0,83	0,59	7,50	L - V	0,48	0,632
	C	2	0,30	0,00	0,00	0,00	N - V	-0,48	0,632
							LN - V	-1,20	0,231
							C - U	-0,48	0,632
							C - N	-1,92	0,055
							C - LN	-1,20	0,231
**SCYB16/CXCL16 (64)**	U	2	0,42	0,02	0,02	0,19	U - V	-1,92	0,055
	V	2	1,85	0,04	0,03	0,32	U - L	-2,40	**0,016**
	L	2	3,92	0,47	0,33	4,19	U - N	-1,44	0,150
	N	2	1,27	0,50	0,36	4,51	U - LN	-0,84	0,401
	LN	2	0,81	0,11	0,08	1,02	L - V	0,48	0,631
	C	2	0,20	0,05	0,04	0,44	N - V	-0,48	0,631
							LN - V	-1,08	0,280
							C - U	-0,48	0,631
							C - N	-1,92	0,055
							C - LN	-1,32	0,187
**SDF-1ab/CXCL12 (22)**	U	2	41,76	1,87	1,32	16,77	U - V	-1,91	0,056
	V	2	417,70	13,70	9,69	123,12	U - L	-2,39	**0,017**
	L	2	725,05	6,53	4,62	58,70	U - N	-0,48	0,633
	N	2	161,25	15,44	10,92	138,69	U - LN	-0,96	0,339

	LN	2	187,19	21,04	14,88	189,00	L - V	0,48	0,633
	C	2	18,33	3,08	2,18	27,70	N - V	-1,43	0,151
							LN - V	-0,96	0,339
							C - U	-0,48	0,633
							C - N	-0,96	0,339
							C - LN	-1,43	0,151
**TARC/CCL17 (67)**	U	2	0,65	0,00	0,00	0,00	U - V	-1,92	0,055
	V	2	8,66	0,60	0,43	5,40	U - L	-2,40	**0,016**
	L	2	19,39	1,09	0,77	9,78	U - N	-1,38	0,168
	N	2	5,62	1,61	1,14	14,42	U - LN	-0,78	0,436
	LN	2	3,44	1,48	1,05	13,28	L - V	0,48	0,631
	C	2	0,43	0,00	0,00	0,00	N - V	-0,54	0,589
							LN - V	-1,14	0,255
							C - U	-0,48	0,631
							C - N	-1,86	0,063
							C - LN	-1,26	0,208
**TERC/CCL25 (46)**	U	2	7,29	0,00	0,00	0,00	U - V	-1,79	0,073
	V	2	29,35	5,75	4,07	51,65	U - L	-2,39	**0,017**
	L	2	81,62	4,93	3,49	44,28	U - N	-1,44	0,151
	N	2	21,55	7,93	5,61	71,22	U - LN	-0,84	0,402
	LN	2	15,48	1,98	1,40	17,79	L - V	0,60	0,550
	C	2	2,78	0,29	0,21	2,60	N - V	-0,36	0,720
							LN - V	-0,96	0,338
							C - U	-0,48	0,632
							C - N	-1,91	0,056
							C - LN	-1,32	0,188
**TNFa (36)**	U	2	0,03	0,00	0,00	0,00	U - V	-0,73	0,466
	V	2	0,71	0,00	0,00	0,00	U - L	-1,70	0,089
	L	2	5,23	0,00	0,00	0,00	U - N	0,00	1,000
	N	2	0,03	0,00	0,00	0,00	U - LN	-1,21	0,225
	LN	2	1,82	0,58	0,41	5,21	L - V	0,97	0,331
	C	2	0,02	0,00	0,00	0,00	N - V	-0,73	0,466
							LN - V	0,49	0,627
							C - U	-0,73	0,466
							C - N	-0,73	0,466
							C - LN	-1,94	0,052

SD: Standart deviation**, SE:** Standard Error**, CI:** Confidence Interval**, U:** Untreated**, V:** VX765**, L:** LPS**, N:** Nigericin**, LN:** LPS&Nigericin**, C:** Champtothesin

P value calculated using Kruskal-Wallis multiple comparison test

Supplemantary Table 5: The effect of stimulation and suppression of NLRP3 inflammasome activation on Δψm.

	Group		Mean Difference	SE	P	95% CI Lower Bound	95% CI Upper Bound
A549	U	V	0,17	0,24	0,948	-0,79	1,13
		L	0,04	0,24	1,000	-0,93	1,00
		N	-0,98	0,24	0,048	-1,94	-0,01
		LN	-1,24	0,24	0,018	-2,21	-0,28
MCF7	U	V	0,33	0,04	0,003	0,16	0,50
		L	0,40	0,04	0,001	0,23	0,57
		N	-2,34	0,04	0,000	-2,51	-2,17
		LN	-2,47	0,04	0,000	-2,64	-2,30
PC3	U	V	0,63	0,12	0,017	0,15	1,11
		L	0,60	0,12	0,021	0,12	1,08
		N	-3,42	0,12	0,000	-3,90	-2,94
		LN	-1,78	0,12	0,000	-2,26	-1,29
U138MG	U	V	-0,40	0,08	0,019	-0,71	-0,09
		L	-0,17	0,08	0,309	-0,48	0,14
		N	-1,46	0,08	0,000	-1,77	-1,15
		LN	-1,08	0,08	0,000	-1,39	-0,77
SH-SY5Y	U	V	-0,95	0,10	0,001	-1,36	-0,54
		L	0,04	0,10	0,990	-0,37	0,46
		N	-1,23	0,10	0,000	-1,64	-0,82
		LN	-0,16	0,10	0,588	-0,57	0,25
Fibroblasts	U	V	0,18	0,09	0,402	-0,19	0,55
		L	-1,12	0,09	0,000	-1,49	-0,75
		N	0,95	0,09	0,001	0,58	1,32
		LN	0,95	0,09	0,001	0,58	1,32

SE: Standard Error**, CI:** Confidence Interval**, U:** Untreated**, V:** VX765**, L:** LPS**, N:** Nigericin**, LN:** LPS&Nigericin**, C:** Champtothesin
P value calculated using Oneway Anova, Post Hoc Tests, Tukey HSD.

Supplemantary Table 6: The effect of VX765, LPS, Nigericin and LPS&Nigericin treatments on VEGF secretion

	Comparison	Z	P		Comparison	Z	P
A549	U - V	0	1	SH-SY5Y	U - V	-1,65145	0,098648
	L - U	-0,99087	0,32175		L - U	-0,16514	0,86883
	N - U	-2,31202	**0,020776**		N - U	-0,33029	0,741182
	LN - U	-1,65145	0,098648		LN - U	0,495434	0,620294
	L - V	-0,99087	0,32175		L - V	-1,81659	0,06928
	N - V	-2,31202	**0,020776**		N - V	-1,98173	**0,047509**
	LN - V	-1,65145	0,098648		LN - V	-1,15601	0,247676
MCF7	U - V	-0,99087	0,32175	U138MG	U - V	-1,32116	0,186449
	L - U	0	1		L - U	0,660578	0,508883
	N - U	-1,15601	0,247676		N - U	-1,32116	0,186449
	LN - U	-1,4863	0,1372		LN - U	-0,66058	0,508883
	L - V	-0,99087	0,32175		L - V	-0,66058	0,508883
	N - V	-2,14688	**0,031803**		N - V	-2,64231	**0,008234**
	LN - V	-2,47717	**0,013243**		LN - V	-1,98173	**0,047509**
PC3	U - V	-0,82572	0,408961	Fibroblasts	U - V	-1,32116	0,186449
	L - U	-0,33029	0,741182		L - U	0,660578	0,508883
	N - U	-1,81659	0,06928		N - U	-1,15601	0,247676
	LN - U	-1,15601	0,247676		LN - U	-0,82572	0,408961
	L - V	-1,15601	0,247676		L - V	-0,66058	0,508883
	N - V	-2,64231	**0,008234**		N - V	-2,47717	**0,013243**
	LN - V	-1,98173	**0,047509**		LN - V	-2,14688	**0,031803**

U: Untreated**, V:** VX765**, L:** LPS**, N:** Nigericin**, LN:** LPS&Nigericin
P value calculated using Kruskal-Wallis multiple comparison test

Supplemantary Table 7: The effect of VX765, LPS, Nigericin and LPS&Nigericin treatments on the secretion of MMP proteins

		A549		MCF7		PC3		SH-SY5Y		U138MG		Fibroblasts	
	Comparison	Z	P	Z	P	Z	P	Z	P	Z	P	Z	P
MMP1													
	U-V	1,58	0,114	ND	ND	1,04	0,301	ND	ND	-1,28	0,200	1,19	0,236
	L-U	-1,58	0,114	ND	ND	-2,07	0,038	ND	ND	0,51	0,608	0,00	1,000
	N-U	-1,58	0,114	ND	ND	-2,07	0,038	ND	ND	-1,11	0,267	-1,19	0,236
	LN-U	-1,58	0,114	ND	ND	-2,07	0,038	ND	ND	-1,11	0,267	-1,19	0,236
	L-V	0,00	1,000	ND	ND	-1,04	0,301	ND	ND	-0,77	0,442	1,19	0,236
	N-V	0,00	1,000	ND	ND	-1,04	0,301	ND	ND	-2,39	0,017	0,00	1,000
	LN-V	0,00	1,000	ND	ND	-1,04	0,301	ND	ND	-2,39	0,017	0,00	1,000
MMP2	U-V	-1,29	0,198	ND	ND	0,00	1,000	0,00	1,000	-1,16	0,246	0,00	1,000
	L-U	0,77	0,440	ND	ND	-1,94	0,053	0,00	1,000	0,33	0,740	-0,50	0,620
	N-U	-1,03	0,303	ND	ND	-1,94	0,053	1,06	0,289	-1,41	0,159	-1,65	0,099
	LN-U	-1,03	0,303	ND	ND	-1,94	0,053	1,30	0,195	-0,91	0,362	-1,98	0,048
	L-V	-0,51	0,607	ND	ND	-1,94	0,053	0,00	1,000	-0,83	0,408	-0,50	0,620
	N-V	-2,32	**0,021**	ND	ND	-1,94	0,053	1,06	0,289	-2,57	**0,010**	-1,65	0,099
	LN-V	-2,32	**0,021**	ND	ND	-1,94	0,053	1,30	0,195	-2,07	**0,038**	-1,98	**0,048**
MMP3	U-V	ND	ND	ND	ND	ND	ND	ND	ND	-1,11	0,267	0,00	1,000
	L-U	ND	ND	ND	ND	ND	ND	ND	ND	0,17	0,864	-1,00	0,319
	N-U	ND	ND	ND	ND	ND	ND	ND	ND	-1,28	0,200	-1,91	0,056
	LN-U	ND	ND	ND	ND	ND	ND	ND	ND	-1,28	0,200	-2,08	0,038
	L-V	ND	ND	ND	ND	ND	ND	ND	ND	-0,94	0,347	-1,00	0,319
	N-V	ND	ND	ND	ND	ND	ND	ND	ND	-2,39	**0,017**	-1,91	0,056
	LN-V	ND	ND	ND	ND	ND	ND	ND	ND	-2,39	**0,017**	-2,08	**0,038**
MMP7	U-V	-1,32	0,186	ND	ND	-0,66	0,509	ND	ND	-0,99	0,322	ND	ND
	L-U	0,66	0,509	ND	ND	1,32	0,186	ND	ND	0,00	1,000	ND	ND
	N-U	-0,66	0,509	ND	ND	-2,48	**0,013**	ND	ND	-1,65	0,099	ND	ND
	LN-U	-1,32	0,186	ND	ND	-2,15	**0,032**	ND	ND	-0,99	0,322	ND	ND
	L-V	-0,66	0,509	ND	ND	0,66	0,509	ND	ND	-0,99	0,322	ND	ND
	N-V	-1,98	**0,048**	ND	ND	-1,16	0,248	ND	ND	-2,64	**0,008**	ND	ND
	LN-V	-2,64	**0,008**	ND	ND	-0,83	0,409	ND	ND	-1,98	**0,048**	ND	ND
MMP8	U-V	ND	ND	ND	ND	ND	ND	ND	ND	ND	ND	ND	ND
	L-U	ND	ND	ND	ND	ND	ND	ND	ND	ND	ND	ND	ND
	N-U	ND	ND	ND	ND	ND	ND	ND	ND	ND	ND	ND	ND
	LN-U	ND	ND	ND	ND	ND	ND	ND	ND	ND	ND	ND	ND
	L-V	ND	ND	ND	ND	ND	ND	ND	ND	ND	ND	ND	ND
	N-V	ND	ND	ND	ND	ND	ND	ND	ND	ND	ND	ND	ND
	LN-V	ND	ND	ND	ND	ND	ND	ND	ND	ND	ND	ND	ND
MMP9	U-V	ND	ND	ND	ND	ND	ND	ND	ND	ND	ND	ND	ND
	L-U	ND	ND	ND	ND	ND	ND	ND	ND	ND	ND	ND	ND
	N-U	ND	ND	ND	ND	ND	ND	ND	ND	ND	ND	ND	ND
	LN-U	ND	ND	ND	ND	ND	ND	ND	ND	ND	ND	ND	ND
	L-V	ND	ND	ND	ND	ND	ND	ND	ND	ND	ND	ND	ND
	N-V	ND	ND	ND	ND	ND	ND	ND	ND	ND	ND	ND	ND
	LN-V	ND	ND	ND	ND	ND	ND	ND	ND	ND	ND	ND	ND
MMP10	U-V	-0,67	0,504	ND	ND	0,00	1,000	ND	ND	-0,99	0,320	-0,58	0,561
	L-U	-0,67	0,504	ND	ND	-0,99	0,322	ND	ND	0,00	1,000	-0,33	0,740
	N-U	-1,50	0,132	ND	ND	-1,65	0,099	ND	ND	-1,41	0,159	-1,25	0,213
	LN-U	-1,84	0,066	ND	ND	-2,31	0,021	ND	ND	-1,24	0,214	-1,91	0,056
	L-V	-1,34	0,181	ND	ND	-0,99	0,322	ND	ND	-0,99	0,320	-0,91	0,361
	N-V	-2,17	**0,030**	ND	ND	-1,65	0,099	ND	ND	-2,40	**0,016**	-1,83	0,068
	LN-V	-2,51	**0,012**	ND	ND	-2,31	**0,021**	ND	ND	-2,24	**0,025**	-2,49	**0,013**
MMP13	U-V	-2,24	**0,025**	0,68	0,494	-0,66	0,509	-0,99	0,322	-0,83	0,409	ND	ND
	L-U	0,81	0,416	-1,03	0,305	-0,66	0,509	0,99	0,322	-0,33	0,741	ND	ND
	N-U	0,00	1,000	-1,03	0,305	-1,98	**0,048**	-2,31	**0,021**	-1,49	0,137	ND	ND
	LN-U	0,00	1,000	-0,68	0,494	-1,32	0,186	-2,31	**0,021**	-1,49	0,137	ND	ND
	L-V	-1,42	0,155	-2,39	**0,017**	-1,32	0,186	0,00	1,000	-1,16	0,248	ND	ND
	N-V	-2,24	**0,025**	-2,39	**0,017**	-2,64	**0,008**	-1,32	0,186	-2,31	**0,021**	ND	ND
	LN-V	-2,24	**0,025**	-1,37	0,171	-1,98	**0,048**	-1,32	0,186	-2,31	**0,021**	ND	ND
MMP12	U-V	ND	ND	ND	ND	-0,70	0,481	ND	ND	0,00	1,000	0,00	1,000
	L-U	ND	ND	ND	ND	-1,06	0,291	ND	ND	-0,99	0,322	-0,99	0,322
	N-U	ND	ND	ND	ND	-1,59	0,113	ND	ND	-2,31	0,021	-1,65	0,099
	LN-U	ND	ND	ND	ND	-1,59	0,113	ND	ND	-1,65	0,099	-2,31	0,021
	L-V	ND	ND	ND	ND	-1,76	0,078	ND	ND	-0,99	0,322	-0,99	0,322
	N-V	ND	ND	ND	ND	-2,29	**0,022**	ND	ND	-2,31	**0,021**	-1,65	0,099
	LN-V	ND	ND	ND	ND	-2,29	**0,022**	ND	ND	-1,65	0,099	-2,31	**0,021**

U: Untreated**, V:** VX765**, L:** LPS**, N:** Nigericin**, LN:** LPS&Nigericin**,**
P value calculated using Kruskal-Wallis multiple comparison test

Supplemantary Table 8: The effect of cytokine secretion from cell lines after VX765, LPS, Nigericin and LPS&Nigericin treatments on the endothelial tube formation assay.

		Type	N	Mean	SD	SE	CI 95 %	**Kruskal-Wallis multiple comparison**	Z	P.unadj
A549	Holes	U	2	6	1,41	1	12,71	U - V	-2,64	**0,008**
		V	2	32	1,41	1	12,71	L - U	1,98	**0,048**
		L	2	28	1,41	1	12,71	N - U	1,32	0,186
		N	2	20	1,41	1	12,71	LN - U	0,66	0,509
		LN	2	11	1,41	1	12,71	L - V	-0,66	0,509
								N - V	-1,32	0,186
								LN - V	-1,98	**0,048**
	Branches	U	2	104	2,83	2	25,41	U - V	-2,64	**0,008**
		V	2	152,5	2,12	1,5	19,06	L - U	1,82	0,069
		L	2	143	1,41	1	12,71	N - U	0,66	0,509
		N	2	130	1,41	1	12,71	LN - U	1,49	0,137
		LN	2	141	2,83	2	25,41	L - V	-0,83	0,409
								N - V	-1,98	**0,048**
								LN - V	-1,16	0,248
MCF7	Holes	U	2	2	0	0	0	U - V	-2,39	**0,017**
		V	2	25,5	2,12	1,5	19,06	L - U	1,7	0,088
		L	2	9,5	0,71	0,5	6,35	N - U	0,43	0,67
		N	2	2,5	0,71	0,5	6,35	LN - U	0,6	0,551
		LN	2	3	1,41	1	12,71	L - V	-0,68	0,496
								N- V	-1,96	0,05
								LN - V	-1,79	0,074
	Branches	U	2	79,5	0,71	0,5	6,35	U - V	-1,32	0,186
		V	2	101,5	2,12	1,5	19,06	L - U	-0,66	0,509
		L	2	68	1,41	1	12,71	N - U	1,98	**0,048**
		N	2	113	2,83	2	25,41	LN - U	0,66	0,509
		LN	2	85,5	3,54	2,5	31,77	L - V	-1,98	**0,048**
								N- V	0,66	0,509
								LN - V	-0,66	0,509
PC3	Holes	U	2	3,5	2,12	1,5	19,06	U - V	-2,58	**0,01**
		V	2	81	1,41	1	12,71	L - U	1,16	0,245
		L	2	8	1,41	1	12,71	N - U	0,58	0,561
		N	2	6	1,41	1	12,71	LN - U	1,91	0,056
		LN	2	13,5	2,12	1,5	19,06	L - V	-1,41	0,158
								N - V	-1,99	**0,046**
								LN - V	-0,66	0,506
	Branches	U	2	118,5	0,71	0,5	6,35	U - V	-2,32	**0,02**
		V	2	225,5	2,12	1,5	19,06	L - U	0,25	0,804
		L	2	119,5	3,54	2,5	31,77	N - U	1,66	0,098
		N	2	132	1,41	1	12,71	LN - U	0,75	0,456
		LN	2	121,5	0,71	0,5	6,35	L - V	-2,07	**0,038**
								N - V	-0,66	0,508
								LN - V	-1,57	0,116
SHSY5Y	Holes	U	2	3	0	0	0	U - V	-2,66	**0,008**
		V	2	39,5	0,71	0,5	6,35	L - U	1,99	**0,046**
		L	2	13	1,41	1	12,71	N - U	0,75	0,455
		N	2	7	1,41	1	12,71	LN - U	1,25	0,213
		LN	2	9	1,41	1	12,71	L - V	-0,66	0,506
								N - V	-1,91	0,056
								LN - V	-1,41	0,158
	Branches	U	2	91	2,83	2	25,41	U - V	-2,64	**0,008**
		V	2	169	2,83	2	25,41	L - U	1,98	**0,048**
		L	2	122,5	3,54	2,5	31,77	N - U	0,83	0,409
		N	2	101	2,83	2	25,41	LN - U	1,16	0,248
		LN	2	105	4,24	3	38,12	L - V	-0,66	0,509
								N - V	-1,82	0,069
								LN - V	-1,49	0,137
U138MG	Holes	U	2	5	0	0	0	U - V	-2,66	**0,008**
		V	2	61,5	2,12	1,5	19,06	L - U	1,99	**0,046**
		L	2	31,5	2,12	1,5	19,06	N - U	0,66	0,506
		N	2	12	1,41	1	12,71	LN - U	1,33	0,184
		LN	2	18	0	0	0	L - V	-0,66	0,506
								N - V	-1,99	**0,046**
								LN - V	-1,33	0,184
	Branches	U	2	97	1,41	1	12,71	U - V	-2,64	**0,008**
		V	2	209	1,41	1	12,71	L - U	1,98	**0,048**
		L	2	161,5	0,71	0,5	6,35	N - U	1,32	0,186
		N	2	141,5	2,12	1,5	19,06	LN - U	0,66	0,509
		LN	2	106,5	0,71	0,5	6,35	L - V	-0,66	0,509
								N - V	-1,32	0,186
								LN - V	-1,98	**0,048**
Fibroblasts	Holes	U	2	108	1,41	1	12,71	U - V	0	1
		V	2	108	1,41	1	12,71	L - U	-1	0,319
		L	2	79	1,41	1	12,71	N - U	-2,33	**0,02**
		N	2	26,5	2,12	1,5	19,06	LN - U	-1,66	0,097
		LN	2	34,5	0,71	0,5	6,35	L - V	-1	0,319
								N - V	-2,33	**0,02**
								LN - V	-1,66	0,097
	Branches	U	2	140,5	2,12	1,5	19,06	U - V	-0,66	0,508

V	2	164	0	0	0	L - U	-0,66	0,508
L	2	137,5	0,71	0,5	6,35	N - U	-1,99	**0,047**
N	2	54,5	2,12	1,5	19,06	LN - U	-1,33	0,185
LN	2	66	1,41	1	12,71	L - V	-1,33	0,185
						N - V	-2,65	**0,008**
						LN - V	-1,99	**0,047**

SD: Standart deviation**, SE:** Standard Error**, CI:** Confidence Interval**, U:** Untreated**, V:** VX765**, L:** LPS**, N:** Nigericin**, LN:** LPS&Nigericin**,**
P value calculated using Kruskal-Wallis multiple comparison test
